# Multi-Strategy-Assisted Hybrid Crayfish-Inspired Optimization Algorithm for Solving Real-World Problems

**DOI:** 10.3390/biomimetics10050343

**Published:** 2025-05-21

**Authors:** Wenzhou Lin, Yinghao He, Gang Hu, Chunqiang Zhang

**Affiliations:** 1 School of Art and Design, Xi’an University of Technology, Xi’an 710054, China; lin2004@xaut.edu.cn (W.L.); cqsay@xaut.edu.cn (C.Z.); 2 Department of Applied Mathematics, Xi’an University of Technology, Xi’an 710054, China; heyinghao@xaut.edu.cn

**Keywords:** crawfish optimization algorithm, elite chaos difference strategy, differential evolution, levy flight, dimension-by-dimension variation, adaptive weighting

## Abstract

In order to solve problems with the original crayfish optimization algorithm (COA), such as reduced diversity, local optimization, and insufficient convergence accuracy, a multi-strategy optimization algorithm for crayfish based on differential evolution, named the ICOA, is proposed. First, the elite chaotic difference strategy is used for population initialization to generate a more uniform crayfish population and increase the quality and diversity of the population. Secondly, the differential evolution strategy and the dimensional variation strategy are introduced to improve the quality of the crayfish population before its iteration and to improve the accuracy of the optimal solution and the local search ability for crayfish at the same time. To enhance the updating approach to crayfish exploration, the Levy flight strategy is adopted. This strategy aims to improve the algorithm’s search range and local search capability, prevent premature convergence, and enhance population stability. Finally, the adaptive parameter strategy is introduced to improve the development stage of crayfish, so as to better balance the global search and local mining ability of the algorithm, and to further enhance the optimization ability of the algorithm, and the ability to jump out of the local optimal. In addition, a comparison with the original COA and two sets of optimization algorithms on the CEC2019, CEC2020, and CEC2022 test sets was verified by Wilcoxon rank sum test. The results show that the proposed ICOA has strong competition. At the same time, the performance of ICOA is tested against different high-performance algorithms on 6 engineering optimization examples, 30 high–low-dimension constraint problems and 2 large-scale NP problems. Numerical experiments results show that ICOA has superior performance on a range of engineering problems and exhibits excellent performance in solving complex optimization problems.

## 1. Introduction

In recent years, optimization techniques have been increasingly employed to tackle complex problems in various disciplines, including science, engineering, and other relevant fields [1,2,3]. The dimensions and sophistication of these optimization problems have grown exponentially [4]. The traditional gradient descent method and Newton method have been proved to be insufficient to meet evolving engineering needs, and still have many defects in dealing with multi-extremum problems. Therefore, a meta-heuristic algorithm with many advantages, such as randomness, flexibility, ease of implementation, and no need for gradient information [5], comes into being. When it comes to optimizing a given problem, meta-heuristic algorithms can strike a balance between designing efficiently from a local optimum to converging at a point. As a result, they increase the likelihood of finding a global optimum. Meta-heuristic algorithms are extensively utilized, owing to their capacity to efficiently determine the global optimum solution for various optimization problems. These algorithms exhibit several desirable features, including independence from initial conditions and solution domains, robustness, and other exceptional qualities [6,7]. Its practical utility has also been evidenced and employed in various fields, including the multiple traveling salesman problem [8], multilevel threshold image segmentation [9], ship routing and scheduling problems [10], feature selection [11], and multilevel image segmentation [12]. Currently, there are four broad categories.

Group intelligence algorithms are inspired by the effects of animal behavior. Take the PSO [13] algorithm for instance, which draws inspiration from the foraging behavior of birds. In this algorithm, the search space is represented by a collection of particles, each of which embodies a potential solution. To improve efficiency, every particle explores both local and global optima during the search process, aiming to achieve enhanced outcomes. Finally, the velocity and weights of the particles are added to obtain the solution. The Artificial Bee Colony Algorithm (ABC) [14], Ant Colony Optimization (ACO) [15], and Cuckoo Search Optimization (CSO) [16] are the classical algorithms proposed after the PSO algorithm. In recent years, there has been a surge of novel algorithms introduced as a result of extensive research. Consider, for instance, the Snake Optimizer (SO) [17], which finds inspiration from the feeding and reproduction behaviors of snakes. Similarly, the White Shark Optimizer (WSO) [18] draws inspiration from the distinctive navigation and foraging characteristics of white sharks. Alongside these, we also have the Harris Hawk Optimizer (HHO) [19], Artificial Rabbit Optimization (ARO) [20], and Artificial Hummingbird Optimization Algorithm (AHA) [21], and various other algorithms with diverse inspirations and applications.

Algorithms that draw inspiration from various physical phenomena are known as physics-based algorithms. Most famously, Rashedi et al. proposed a physics-based gravitational search algorithm (GSA) [22]. Another renowned physics-based algorithm is the Simulated Annealing Algorithm (SA) [23], which emulates the annealing process of solid matter in the field of physics. These notable examples highlight the application of principles from physics to optimize algorithm design. Evolutionary algorithms are meta-heuristic algorithms that mimic natural evolutionary mechanisms. A prominent example of a physics-based algorithm is the Genetic Algorithm (GA) [24], which simulates the process of biological evolution through natural selection and the hereditary mechanism described in Darwin’s theory. GA uses binary encoding to facilitate interbreeding and mutation in a population of organisms, with each individual representing a candidate solution. Additionally, several new evolutionary algorithms have emerged, including Evolutionary Programming (EP) [25], Differential Evolution (DE) [26], and the Virulence Optimization Algorithm (VOA) [27]. These algorithms draw inspiration from biological and evolutionary principles to optimize their design.

Meta-heuristic algorithms that draw inspiration from human behavioral habits are categorized as human-inspired algorithms [28]. One prominent example in this category is the Harmony Search (HS) algorithm [29]. Other algorithms that share similar principles include Teaching–Learning-Based Optimization (TLBO) [30], Social Group Optimization (SGO) [31], the Group Teaching Optimization Algorithm (GTOA) [32], the Brain Storming Optimization Algorithm (BSO) [33], and the Imperialist Competition Algorithm (ICA) [34]. These algorithms leverage human behavioral patterns to optimize their problem-solving approaches.

In summary, by comparing different features and advantages of meta-heuristic algorithms, the following conclusions can be drawn:

- They are usually inspired by some natural law or mathematical theory [35].
- No theoretical derivation is required to transform the problem into a model that is less dependent on mathematical conditions [36].
- The complexity of the algorithm determines its search rate for finding approximate and suitable solutions [37].

There are two main ways to solve the problem in the meta-heuristic algorithm: (1) a single solution; and (2) A total-based solution. A single-solution-based algorithm that starts its process with a candidate solution and improves on it during iteration. A population-based meta-heuristic algorithm initializes the random solution so that the population tends to the most promising region. Compared to algorithms based on a single solution, population-based meta-heuristics have two advantages: exploration and exploitation [38,39]. The two progress each other, making the algorithm converge faster and improving the global search capability.

According to the No Free Lunch Theory (NFL) [40], there is no perfect algorithm, and there must be a disadvantage to this in some aspect. Based on crawfish’s foraging behavior and summer resort behavior, scholars proposed a new meta-heuristic optimization algorithm, namely the Crawfish Optimization Algorithm (COA) [41]. The COA is a novel way to model the foraging, summer resort, and competitive behaviors of crayfish. By adjusting the temperature, the exploration and development ability of the algorithm are balanced. This algorithm focuses on modeling crayfish behavior and balancing the algorithm by adjusting the temperature. Through a comparative analysis of experimental results, it is observed that the COA demonstrates favorable optimization performance in CEC2014 benchmark functions, as well as in 23 standard benchmark functions and engineering application problems.

However, the COA has its limitations, for instance, low population diversity, poor convergence speed, and low global accuracy of solutions. To address these limitations and improve its optimization capabilities, the paper proposes an enhanced version of the algorithm called the ICOA. The proposed approach includes several improvements. Firstly, an elite chaotic difference strategy is introduced in the initialization stage to promote a more even distribution of crayfish and to obtain an initial population of higher quality. Additionally, to augment the population’s diversity and quality, a differential evolution strategy is integrated before the summer vacation. Moreover, during crayfish summer resort behavior, a Levy flight strategy is employed to amplify their search capability, broaden their exploration range, and enhance their capacity to evade local optima. Finally, an adaptive strategy is introduced during the competition phase to bolster the crayfish’s global search capability, resulting in enhanced solution accuracy and faster convergence in successive iterations.

The efficacy and high competitiveness of the proposed ICOA is demonstrated through comprehensive experimentation on diverse problem sets. Specifically, we evaluate the performance of the ICOA on the CEC2017, CEC2020, and CEC2022 test sets, six engineering applications, 30 high- and low-dimensional constrained problems, and two NP problems. In addition, we evaluated its ability to solve hypersonic missile trajectory planning problems. To assess the effectiveness of the ICOA, a comprehensive comparison is conducted between its solutions and those generated by classical and advanced optimization algorithms. The results obtained were rigorously evaluated by statistical analysis using Wilcoxon rank sum tests to ensure that any significant differences were identified. The key contributions of this research can be summarized as follows:

(i) The ICOA is proposed by incorporating four strategies, namely, the elite chaotic differential strategy, differential variation strategy, Levy flight strategy, dimensional variation strategy, and adaptive parameter strategy.
(ii) The effectiveness and potential of the ICOA in addressing complex optimization problems are validated through experimental results obtained from benchmark test sets such as CEC2017, CEC2019, and CEC2020. These results are compared with other state-of-the-art swarm intelligence optimization algorithms, revealing the superior performance of the ICOA. This comparative analysis highlights the algorithm’s effectiveness and reinforces its capability to tackle challenging optimization problems.
(iii) The ICOA is applied to various real-world industrial design problems, including six specific cases. In addition, thirty high–low-dimension constraint problems, two NP problems, and one hypersonic missile trajectory planning problem are evaluated. The performance of the ICOA is methodically compared to that of classical or state-of-the-art optimization algorithms, providing insights into its efficacy and applicability across diverse problem domains.

The following section of this study will be structured as outlined: Section 2 introduces the inspiration and mathematical model of COA. Section 3 proposes the ICOA, introduces the elite chaotic difference strategy, the difference variance strategy, and the Levy flight strategy, as well as the dimensional variation strategy and the adaptive parameter strategy, and analyzes the algorithmic complexity of ICOA. In Section 4, we conduct numerical experiments and analyze the results of ICOA in comparison to other optimization algorithms using the CEC2017, CEC2020, and CEC2022 test sets. In Section 5, the proposed ICOA is used to solve 6 practical industrial design problems, 30 high–low-dimension constraint problems and two NP problems. In Section 6, a reasonable discussion is presented on the work of this paper. Finally, in Section 7, this paper provides concluding remarks and suggestions for future research directions.

## 2. Overview of the Crawfish Optimization Algorithm

**Figure 1:**
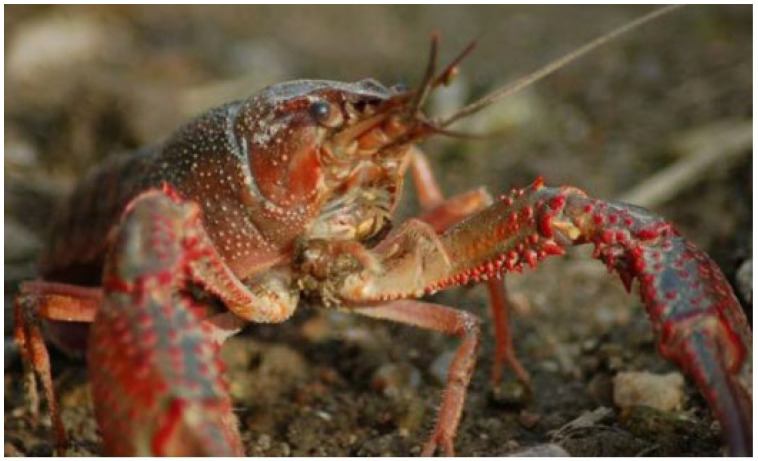
Schematic diagram of crayfish.

In 2023, Heming Jia et al. [41] introduced the Crayfish Optimization Algorithm (COA), drawing inspiration from crayfish foraging, summer vacation, and competitive behaviors. The algorithm simulates scenarios where crayfish are on summer vacation, competing, or foraging, and identifies the best location based on varying temperatures. Figure 1 is a schematic diagram of crayfish derived from the literature [41].

The COA is initialized as shown in Equation (1):

(1) $$AX=[AX_{1},AX_{2},\ldots ,AX_{N}]=\left[\begin{array}{l} AX_{1,1}\;\;\ldots \;AX_{1,j}\;\ldots AX_{1,dim}\\ \;\;\;\vdots \;\;\;\;\;\;\;\ldots \;\;\;\;\vdots \;\;\;\ldots \;\;\vdots \\ AX_{i,1}\;\;\ldots \;AX_{i,j}\;\;\ldots AX_{i,dim}\\ \;\;\;\vdots \;\;\;\;\;\;\;\ldots \;\;\;\;\vdots \;\;\;\ldots \;\;\vdots \\ AX_{N,1}\ldots \;AX_{N,j}\ldots AX_{N,dim} \end{array}\right],$$

where ***AX*** is the initial population position, *N* is the number of populations, *dim* is the number of population dimensions, $AX_{i,j}$ is the position of individual *i* in dimension *j*, and the value is obtained from Equation (2).

(2) $$AX_{i,j}=l_{j}+(u_{j}-l_{j})\times r,$$

where $l_{j}$ denotes the lower bound of the *j*-th dimension, $u_{j}$ denotes the upper bound of the *j*-th dimension, and *r* denotes a random number.

Changes in temperature affect the behavior of crayfish at different stages, which are defined by Equation (3).

(3) t=r×15+20,

where *t* denotes the ambient temperature and *r* is a random number.

(4) $$p=C_{1}\times \left(\frac{1}{\sqrt{2\times \pi }\times \sigma }\times \exp\left(-\frac{{\left(t-\mu \right)}^{2}}{2\sigma^{2}}\right)\right),$$

where *µ* is the temperature most suitable for crayfish, *p* is the foraging intake, and *σ* and *C*_1_ are used to control the intake of crayfish at different temperatures.

### 2.1. Summer Vacation

When the temperature reaches a critical point, the crayfish will go into their burrows and start vacationing. The burrows $AX_{sh}$ are ventilated as follows:

(5) $$AX_{sh}=(AX_{g}+AX_{l})/2,$$

where ***AX_g_*** denotes the final optimal position and ***AX_l_*** denotes the current optimal position.

**Figure 2:**
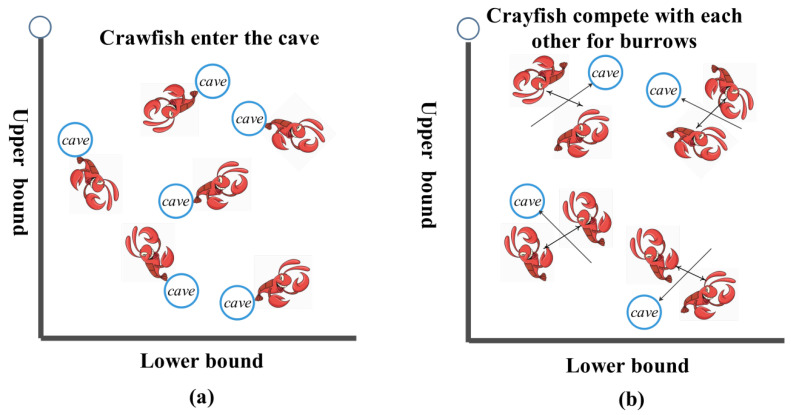
Crayfish summer vacation chart. (**a**) Enter the burrow. (**b**) Get into the cave fight.

When *rand* < 0.5, the crayfish will avoid the heat by Equation (6), as shown in Figure 2a.

(6) $$AX_{i,j}^{t+1}=AX_{i,j}^{t}+C_{2}\times r\times (AX_{sh}-AX_{i,j}^{t}),$$

where *t* is the current iteration number, *t* + 1 then indicates the next iteration, and *C*_2_ is the decreasing curve, as shown in Equation (7).

(7) $$C_{2}=2-(t/T),$$

where *T* is the maximum value of the number of iterations.

### 2.2. Competition Stage

As shown in Figure 2b, when the *t* > 30C and *r* ≥ 0.5, crayfish behave according to Equation (8) for burrowing competition.

(8) $$AX_{i,j}^{t+1}=AX_{i,j}^{t}-AX_{z,j}^{t}+AX_{sh},$$

where *t* is the temperature and *r* is the random probability. *Z* is a random individual, as defined in Equation (9).

(9) Z=round(r×(N−1))+1.

### 2.3. Formalization Stage

When the temperature is 30 °C, this is the right time for crayfish to forage for food. At this point, the food position ***AX****_food_* is as in Equation (10):

(10) $$AX_{food}=AX_{G},$$

The size of the food, denoted as *AQ*, is defined as follows:

(11) $$AQ=C_{3}\times rand\times (fitness_{i}/fitness_{food}),$$

where *C*_3_ is defined as the largest food item with a constant value of 3, $fitness_{i}$ is the fitness value of the *i*-th individual, and $fitness_{food}$ is the fitness value at the time of foraging for food.

When *AQ* > (*C*_3_ + 1)/2, crayfish forage for food through Equation (12).

(12) $$AX_{food}=\exp\left(-\frac{1}{AQ}\right)\times AX_{food},$$

When the food item is small, foraging at this time is shown in Equation (13).

(13) $$AX_{i,j}^{t+1}=AX_{i,j}^{t}+AX_{food}\times p\times (\cos(2\times \pi \times r)-\sin(2\times \pi \times r)),$$

When *Q ≤* (*C*_3_ + 1)/2, crayfish feed as shown in Equation (14).

(14) $$AX_{i,j}^{t+1}=\left(AX_{i,j}^{t}-AX_{food}\right)\times p+p\times rand\times AX_{i,j}^{t}.$$

At this point, the crayfish has completed all of its behavioral manifestations, that is, it has completed the theoretical process of the COA.

Algorithm 1 is given as pseudo-code for the COA.
**Algorithm 1:** Crayfish optimization algorithmBegin     Step 1: Initialization. Set the parameters of the crayfish population.     Step 2: Fitness calculation. By calculating the fitness value of the initialized population to get $X_{g},X_{l}$.     Step 3: while termination criteria are not met do                  Defining temperature temp by Equation (3)                      if *temp* > 30 do                      Define cave $X_{sh}$ according by Equation (5)                        if *rand* < 0.5 do                          Crayfish conducts the summer resort stage according to equation (6)                        else                            Crayfish compete for caves through Equation (8)                      end                  else                          *P* and *Q* can be found by Equation (4) and Equation (11), respectively.                          if *Q* ≥ 2 do                              Crayfish shreds food by Equation (12)                              Crayfish foraging according to Equation (13)                          else                                 Crayfish foraging according to Equation (14)                          end                  end                  Update fitness value and output $X_{g},X_{l}$.          end while

## 3. Improved Crayfish Optimization Algorithm with Mixed Strategies

Although the COA has advantages, such as strong optimization ability and fast convergence speed, it is inevitable that it is prone to fall into the local optimum, resulting in low calculation accuracy and affecting optimization ability. In order to further improve the performance of the COA, this section combines the following four strategies to improve the COA and propose the ICOA.

### 3.1. Elite Chaos Difference Strategies

Chaotic mapping is a stochastic method in nonlinear dynamical systems [42] that initializes populations by using chaotic variables instead of random variables [43]. Therefore, it can perform a thorough search of the solution space more efficiently than a random search that relies primarily on probability [44]. Therefore, this paper considers a well-known type of chaotic mapping: logistic chaotic mapping.

The initial population is considered to be divided into three parts, which are as follows:

(1) Elite learning selects the population according to a certain ratio as the first part of the initialization decomposition.

(15) $$EX_{i}^{d}\;=e\times (UB-LB)+LB,i=1,2,\ldots ,\left[\left.N/10\right]\right.,$$

where *N* denotes the number of populations, $EX_{i}^{d}$ refers to the *i*-th crayfish, *UB* and *LB* are the upper and lower bounds of the *d*-th dimension of the population, and *e* is the elite probability, which is taken as 0.1 in this paper.

(2) Logistic chaotic mapping is carried out on the remaining population according to the ratio column as the second part of the initialization solution.

(16) $$\left\{\begin{array}{l} x_{t+1}=\mu x_{t}(1-x_{t}),\;t=1,2,\ldots ,\left[\left.N/5\right]\right.\;\\ CX_{i}^{d}=x_{t+1}\times (ub-lb)+lb, \end{array}\right.$$

where *ub* and *lb* are the upper and lower bounds of the *d*-th dimension of the population, μ∈[0,4], x∈ [0,1]. In this paper, *μ* = 3.9, *x* = 0.5, and *x_t_*_+1_ is denoted as the newly generated chaotic mapping values, and $CX_{i}^{d}$ is the second part of the candidate solution.

(3) Differential learning, which is performed on the remaining populations, randomizes the elite populations as well as the chaotically mapped populations to be updated by the differential operation to obtain the final differential population, which is the last part of the initial solution.

(17) $$\left\{\begin{array}{l} t_{j+1}=t_{j}+0.5\times (EX_{j}-t_{j}),\\ DX_{i}^{d}=lb+t_{j+1}\times (ub-lb), \end{array}\right.$$

where *t_j_* is some solution of the chaotic mapping population, *ub* and *lb* are the upper and lower bounds of the *d*-th dimension of the population, ***EX****_j_* is some candidate solution of the elite learning population, and *t_j_*_+1_ denotes the new solution after differentiation, and $DX_{i}^{d}$ is the last part of the initialization.

The candidate solutions of the above three components are the final initialized crayfish population, expressed as Equation (18).

(18) $$X_{i}^{d}=[EX_{i}^{d},CX_{i}^{d},DX_{i}^{d}],$$

Performing elite chaotic difference initialization prior to crayfish summer vacation improves the quality of the initial population, allowing for faster access to higher-quality solutions.

### 3.2. Differential Variation Strategy

By employing the differential mutation strategy, the algorithm effectively enhances the quality of the population. This strategy plays a crucial role in expanding the search range of the population, preventing it from getting trapped in local optima. As a result, the search ability of the algorithm is significantly improved. Therefore, this section considers two operations of differential evolutionary algorithms: the variation operation and the selection operation.

(1) Mutation operation

To determine the mutated individual, we calculate the vector difference between two randomly chosen individuals from the population, following a scaling process. We also consider the vector synthesis of the individual that is being mutated. This approach ensures a diverse set of genetic information is incorporated into the mutated individual. The mathematical representation for the mutation operation is as follows:

(19) $$U_{t+1}=x_{r1}(t)+F\times (x_{r2}(t)-x_{r3}(t)),\;i\neq r1\neq r2\neq r3,$$

where the scaling factor F=0.9 and $x_{ri}(t)$ denotes the *ri*-th individual in the *t*-th generation population.

(2) Selection operation

After the mutation operation is completed, Levy flight operation is performed on the mutant individual $U_{t+1}$. The greedy choice is made between the subsequent individual $U_{t+1}$ and the original target individual $LU_{t+1}$, and the individual with good fitness value is selected to enter the next iteration. The greedy choice model is as follows:

(20) $$U_{t+1}=\left\{\begin{array}{l} LU_{t+1}\;\;\;\;f(LU_{t+1})<f(U_{t+1})\\ U_{t+1}\;\;\;\;\;\;f(LU_{t+1})\geq f(U_{t+1}) \end{array}\right.,$$

**Figure 3:**
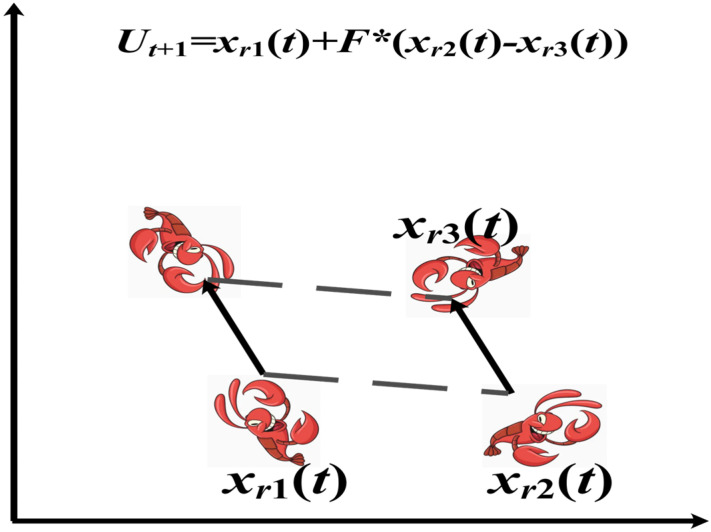
Mutation operation.

By introducing the differential evolution strategy before the crayfish summer vacation, it is beneficial to consider other individuals in this iteration to update the population information of the current iteration, effectively avoiding the situation where the previous solution is a local optimal solution. It also ensures that better individuals will be generated after the initialization of crayfish individuals and randomly learn from the previous generation of individuals, which increases the diversity of the population, expands the search range of the population, effectively avoids premature stagnation of the algorithm, and enhances its ability to jump out of the local optimum. Figure 3 shows the crayfish undergoing the mutation operation.

### 3.3. Levy Flight Strategy

Levy flight refers to the use of Levy flight distribution to simulate the random process of flight paths [45,46], which can describe complex random motion trajectories and phenomena with remote correlation and multi-scale properties. The crawfish burrowing process based on this strategy is shown in Equation (21).

(21) $$X_{new}=X_{i}+\gamma ⊗Levy⊗(Xf-X_{i}),$$

where ***X****_new_* is the new position obtained after the Levy-based flight, ***X****_i_* is the *i*-th crayfish, ***Xf*** is the cave that the crayfish is going to, γ is the step scaling factor, which is generally taken as 0.01, and Levy is the Levy randomized path defined as shown in Equation (22), and ⊗ is the dot product operation.

(22) $$Levy(\beta )\sim u=t^{-\beta },\;1\leq \beta \leq 3,$$

**Figure 4:**
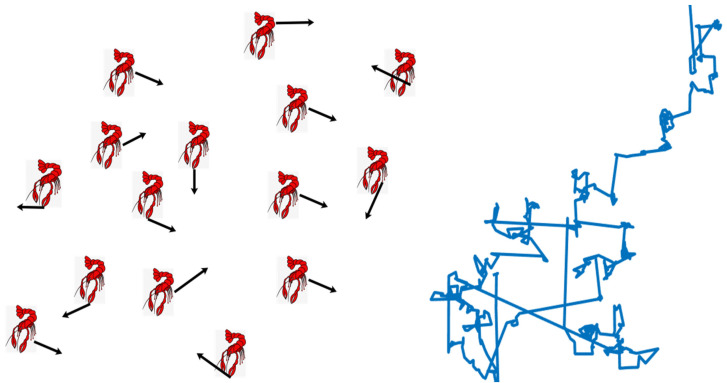
Simulated trajectory plot of Levy flight.

For ease of computation, define the random numbers as follows,

(23) $$s=\frac{u}{{\left|\left.v\right|\right.}^{1/\beta }},$$

(24) $$\;\;\;u\sim N(0,\sigma^{2}),v\sim N(0,1),$$

(25) $$\sigma ={\left\{\left.\frac{\Gamma \left(1+\beta \right)\sin\left(\frac{\pi \beta }{2}\right)}{\beta \Gamma \left(\frac{1+\beta }{2}\right)2^{\frac{\beta -1}{2}}}\right\}\right.}^{1/\beta },$$

where *u* obeys a Gaussian distribution, *v* obeys a normal distribution in Equation (24). *σ* is defined as Equation (25), and *Γ*(*x*) is a gamma function with *β* taking the value of 1.5. Figure 4 shows a plot of the simulated trajectory of the Levy flight.

The Levy flight strategy greatly expanded the range of the crayfish’s search when they explored their burrows during the summer vacation, increasing the variety of the search process. The search ability of the algorithm is improved, and the ability to jump out of the local optimal is enhanced.

### 3.4. Dimensional Variation and Adaptive Parameter Strategy

#### 3.4.1. Dimensional Variation Strategy

For the new population generated after the differential evolution strategy, the *t*-distribution variational operator is introduced to perturb the optimal individual position [47]. The freedom parameters of the *t*-distribution variational operator vary with the number of iterations, and the dimensionally variational strategy is specifically defined as follows: assuming dimension equals *d* and the current optimal solution $g_{best}=(g_{best}^{1},g_{best}^{2},\ldots g_{best}^{d})$, then the new solution, $g_{new}=(g_{new}^{1},g_{new}^{2},\ldots ,g_{best}^{d})$ obtained by mutating the current optimal solution dimension by dimension, is computed as follows:

(26) $$g_{new}^{d}=g_{best}^{d}+\mathrm{TD}{\left(t\right)}^{d}\times g_{best}^{d},$$

where *t* is the current number of iterations, TD(t) is the *t*-distribution with a parameter *t* of degrees of freedom, and $\mathrm{TD}{\left(t\right)}^{d}$ is the random number generated by the *t*-distribution in the *d*-th dimension. Since it is impossible to directly judge whether the new position obtained after mutation is better than the original position, this paper uses the principle of greed to judge whether to accept the new position instead of the original optimal position. The greedy principle is used to guide the population to better evolve to the optimal individual position and improve the convergence speed of the algorithm.

#### 3.4.2. Adaptive Parameter Strategy

Inspired by the crayfish entering the burrow during summer vacation, a variable *C*_1_ is set to control the crayfish competition for the burrow, and the decreasing curve *C* is transformed and applied to the competition phase. For the two random individuals ***X****_i_* and ***X****_j_* in the competition stage, vector difference is carried out through Equation (27), and adaptive parameter changes are carried out, and then the original ***XF****_j_* is added to finally obtain the new position ***XN****_i,j_*.

(27) $$XN_{i,j}=C\times (X_{i}-X_{j})+XF_{j},$$

(28) $$C_{1}=F\times 2^{lamuda},$$

(29) $$lamuda=\exp{\left(1\right)}^{\frac{1-T}{T+1-t}},$$

where *C* represents the adaptive parameter, *F* is set to 0.4, and *t* and *T* represent the current and maximum number of iterations, respectively.

**Figure 5:**
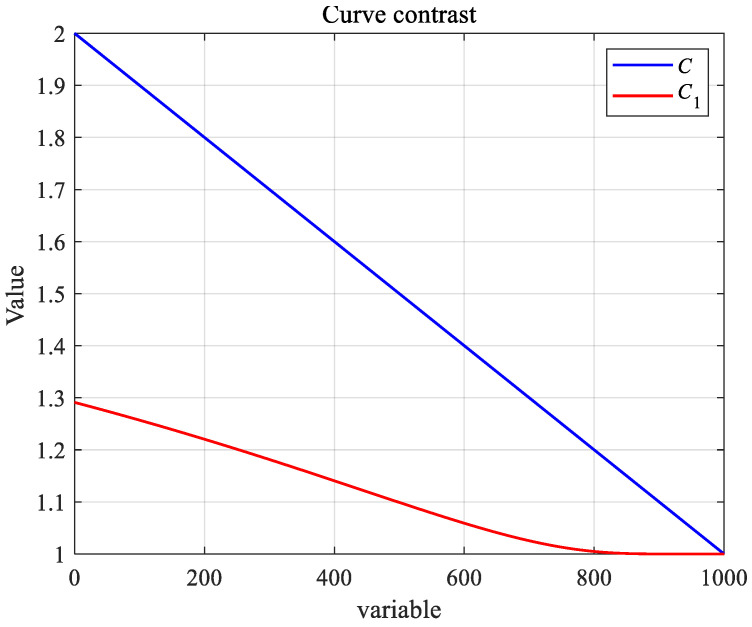
Curve diagrams of *C* and *C*_1_.

The adaptive parameter strategy greatly improves the global search capability of the algorithm during the crayfish competition phase and drastically improves the convergence ability of the algorithm. Figure 5 shows the variation in the *C* and *C*_1_ curves.

### 3.5. ICOA Pseudo-Code

**Figure 6:**
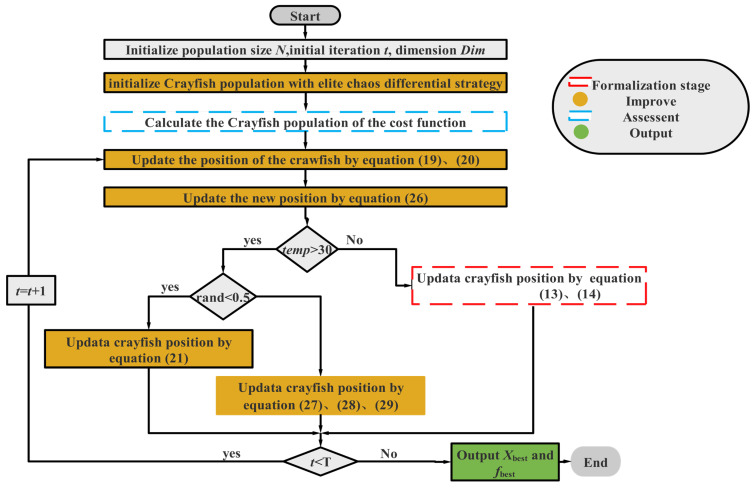
Flowchart of the ICOA.

Algorithm 2 gives the pseudo-code of the ICOA, and the flowchart is shown in Figure 6.
**Algorithm 2:** The proposed ICOABegin     Step1: Initialization. Crayfish populations were initialized using the elite chaos differential strategy (i.e., Equation (18)).     Step2: Fitness calculation. By calculating the population fitness value *fitness*, the optimal fitness $f_{best}$ value as well as the corresponding individuals $X_{best}$ were recorded;     Step3: While (*t* < *T*) do                     Defining temperature temp by Equation (3)                         for *i* = 1 to *N* do                         $U_{t+1}=x_{r1}(t)+F\times (x_{r2}(t)-x_{r3}(t)),\;i\neq r1\neq r2\neq r3,$    //Mutation operation                             $U_{t+1}=\left\{\begin{array}{l} LU_{t+1}\;\;f(LU_{t+1})<f(U_{t+1})\\ U_{t+1}\;\;\;\;\;\;f(LU_{t+1})\geq f(U_{t+1}) \end{array}\right.$    //Select operation                         end for                             $g_{new}^{d}=g_{best}^{d}+TD{\left(t\right)}^{d}\times g_{best}^{d}$ //Dimensional variation                         for *i* = 1 to *N* do                             if *temp* > 30 do        //Summer resort stage and competition stage                                  if *rand* < 0.5 do                                        $X_{new}=X_{i}+\gamma ⊗Levy⊗(Xf-X_{i})$                                  Else                 //Competition stage                                    For *j =* 1 to *Dim* do                                       z=round(rand×(N−1))+1                                       $Xnew_{i,j}=C\times (X_{i}-X_{j})+XF_{j}$                                       C1=F*2^lamuda                                       lamuda=exp(1)^(1−T/(T+1−t))                                 end for                               end                             else             //forging stage                               $p=C_{1}\times \left(\frac{1}{\sqrt{2\times \pi }\times \sigma }\times \exp\left(-\frac{{\left(temp-\mu \right)}^{2}}{2\sigma^{2}}\right)\right)$                             if *P* >2 do                               $X_{food}=\exp\left(-\frac{1}{Q}\right)\times X_{food}$                         else                             $X_{i,j}^{t+1}=\left(X_{i,j}^{t}-X_{food}\right)\times p+p\times rand\times X_{i,j}^{t}$                         end                         end for                         Calculate and rank the fitness values.                         Update $X_{new}$                             *t* = *t*+1        Step4: Return. Return the optimum position $X_{best}$ and fitness value $f(X_{best})$ of Crayfish End

### 3.6. Time Complexity of the ICOA

The time complexity of ICOA is commonly dependent on the population size *N*, the dimension *D*, the objective function value *f* for each iteration, and the maximum number of iterations *T*. The time complexity of ICOA is calculated based on these factors, as shown in Equation (30), Figure 6 shows the flowchart of the ICOA.

(30) $$\begin{array}{l} O(I\mathrm{COA})=O(\mathrm{Norms})+O(\mathrm{initialize})+O(\mathrm{DE})+O(\cos t)+O(\mathrm{update})\\ \;\;\;\;\;\;\;\;\;\;\;\;\;\;\;=O(1)+O(ND)+O(TND)+O(TNf)+O(TND)\\ \;\;\;\;\;\;\;\;\;\;\;\;\;\;\;=O(1+ND+2TND+TNf), \end{array}$$

Since the parameter *T* is generally set very large, the simplified Equation is as follows (31):

(31) O(ICOA)≅O(TN(2D+f)).

## 4. Numerical Experiment and Analysis

In this section, a set of test functions is employed to assess and analyze the performance of the ICOA. The CEC 2017, CEC 2020, and CEC 2022 test sets, consisting of 29, 10, and 12 test functions, respectively, are used to compare the results of the ICOA with those of other classical or recent optimization algorithms. The population size for all algorithms is 50, while the dimension is set at 10 with a maximum number of iterations of 1000. To minimize the influence of randomness on the algorithms, each algorithm is executed independently for 20 trials on the test functions, and the outcomes are compared among the primary algorithm sets.

### 4.1. ICOA Is Compared with the First Group of Optimization Algorithms

#### 4.1.1. Comparison of the Test Set CEC 2020

In this section, a comparative analysis will be conducted between the proposed ICOA and nine other intelligent optimization algorithms. The selected algorithms can be categorized into three groups: (1) classical intelligent optimization algorithms such as the PSO [14] and the DE [27]. (2) Newly proposed intelligent optimization algorithms in the last year include the Fox for Auricular Fox Algorithm (FFA) [48], the Chernobyl Disaster Optimizer (CDO) [49], and the original Crayfish Optimization Algorithm (COA) [41]. (3) Representative intelligent optimization algorithms in recent years include the Gray Wolf Optimization Algorithm (GWO) [50], HHO [20], the Zebra Optimization Algorithm (ZOA) [51], and the Sparrow Optimization Algorithm (SSA) [52]. Table 1 provides the initial parameters of all optimization algorithms.

Table 2 presents the findings from 20 separate runs of the ICOA compared to other algorithms on the 10-dimensional CEC 2020 test set. The results include the mean, standard deviation, and *p*-value obtained from the Wilcoxon rank sum test. The ICOA serves as the benchmark, and the statistical outcomes are reported based on 20 runs at a 95% confidence level (α=0.05). In this case, “+” means that the ICOA performs significantly poorly compared to other algorithms, “=” means that there is no significant difference between the ICOA and the algorithm being compared, and “−” and “+” mean the opposite. The bolded data represent the optimal average values and minimum variances of the nine algorithms on each test function.

According to the results shown in Table 2, the ICOA performs best in all ten tested functions. Its optimization ability proves to be better than the original COA in all cases. This indicates that the four strategies in the original COA positively affect the ICOA by improving its convergence speed and computational accuracy. As far as the test function F1 is concerned, the worst value, the mean value, and the standard deviation of the ICOA are significantly smaller compared to other optimization algorithms. These values are also very close to the optimal values. In addition, based on the average ranking, it can be concluded that the ICOA is ranked first (rank = 1). The last row of Table 2 shows the results of the Wilcoxon rank sum test and the ranking of the algorithms. It can be seen that the results of the COA, DE, PSO, CDO, FFA, SSA, HHO, and ZOA are 0/0/10, while the results of the GWO are 0/3/7. The comprehensive analysis shows that the ICOA outperforms the other comparative algorithms on the CEC 2020 test set, and it has a strong competitive advantage.

**Figure 7:**
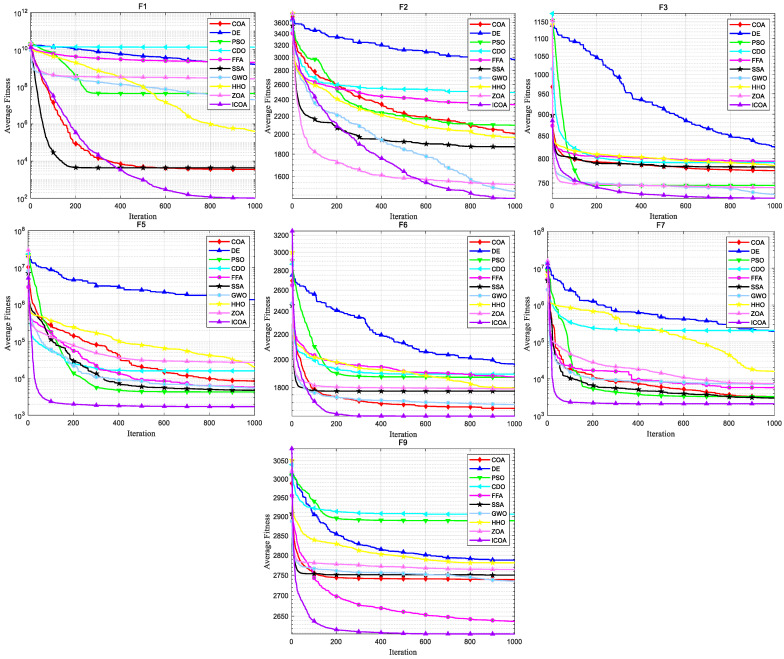
Convergence curve of ICOA and other algorithms for solving 10-dimensional CEC 2020 test set.

Figure 7 shows the convergence curve of each algorithm on the CEC 2020 test set. It can be intuitively seen that the COA falls into local optimality on F1, F2, F3, F5, F7, and F9, leading to premature convergence, while the ICOA’s convergence speed and accuracy are greatly improved compared with the COA. And most of the test functions do not stop in the later iteration stage, but continue converging. Comparing the convergence curves of other algorithms, we can see that the ICOA has faster convergence speed and higher convergence accuracy. Especially in F5, F6, F7, F10, the ICOA’s advantage is more obvious.

**Figure 8:**
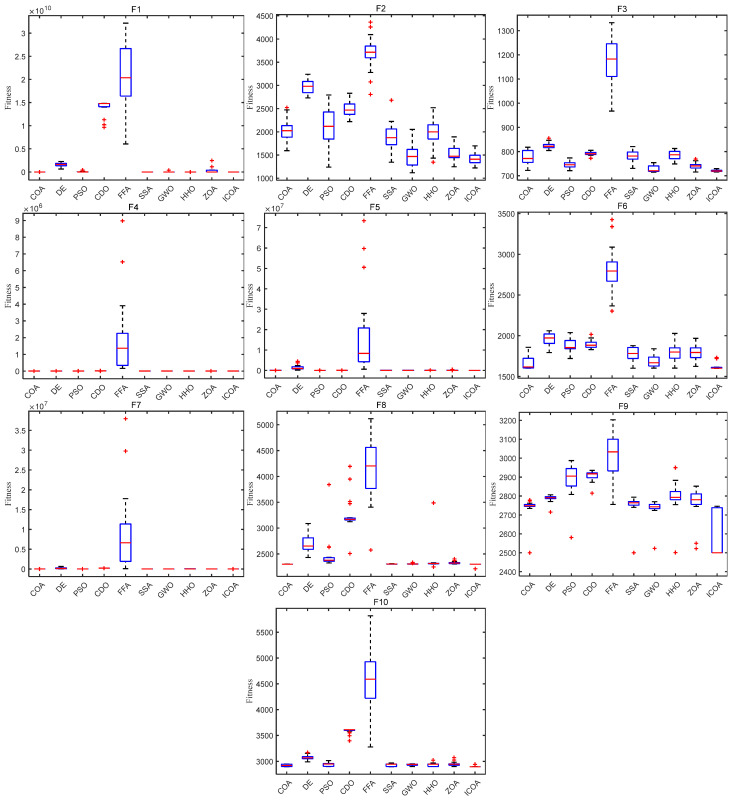
ICOA and other algorithms to solve box diagram of 10-dimensional CEC 2020 test set.

Figure 8 shows the box diagram of each algorithm on the CEC 2020 test function. It can be seen that on most test functions, the ICOA has a narrow box type, low position, and small median, which indicates that the solution obtained by the ICOA has high consistency and high precision. In addition, the ICOA has fewer outliers on the 10 test functions, which indicates that the algorithm is less affected by the randomness of the strategy during operation. It can be seen from the box diagram that the ICOA is superior to other comparison algorithms in terms of stability and accuracy.

#### 4.1.2. Comparison on Test Set CEC 2022

In order to better verify the performance of the proposed ICOA, a new test set from 2022 was selected for testing. The experiment sets the population size *N* of all algorithms as 50, the dimension *dim* as 20, and the maximum number of iterations *T* as 1000.

Table 3 displays the mean, standard deviation, and Wilcoxon rank sum test *p*-values for the ICOA and other optimization algorithms based on 20 independent runs on the CEC 2022 test set. The bolded data represent the optimal average values and minimum variances of each test function. As can be seen from Table 3, the optimization ability of the ICOA on 12 test functions has been significantly improved compared with that of the ICOA. On the test functions F1, F2, F6, F7, F8, F9, and F10, the ICOA achieved the best fitness value while also having a relatively small variance. From the last row of Table 3, the Wilcoxon rank sum test statistics results and the rankings of each algorithm are combined to obtain the Wilcoxon rank sum test results of the nine optimization algorithms as follows: 0/0/12, 0/0/12, 0/0/12, 0/0/12, 0/0/12, 1/0/11, 1/1/10, 0/0/12, and 0/1/11. It can be seen that the ICOA outperforms the COA on all the test functions. In general, the ICOA effectively improves the performance of the original algorithm and shows strong ability in solving the CEC 2022 test set. In addition, in Table 3, rank refers to the ranking of each algorithm for each test function based on the size of the average, mean rank refers to the average ranking of the 12 functions, and result is the final algorithm ranking on the CEC 2022 test function. ICOA ranked first place in 10 functions, mean rank, and result in the comprehensive comparison of the algorithms. This shows that the ICOA has better performance not only on most of the proposed test functions, but also on the whole CEC 2022 test set. In conclusion, the ICOA greatly improves the performance of the original algorithm and demonstrates significant potential in solving the CEC 2022 test set.

To further demonstrate the performance of the proposed ICOA, we chose to use Student’s parametric test (*t*-test) [53,54] for statistical evaluation. T-p in Table 3 is the *p*-value of the *t*-test, and the statistical results are given by running the ICOA 20 times at the 95% significance level (*t* = 0.05), and the experimental results show that ICOA is significant for the original COA on 83.3% of the test functions (10 out of 12 test functions). The other eight comparison algorithms are 100%, 58.3%, 100%, 100%, 100%, 91.7%, 58.3%, 100%, and 91.7%. It can be seen that the ICOA is more than 90% significant for all the other algorithms, and only two algorithms are significant at 58.3% on the CEC 2022 test set. In conclusion, the ICOA improves the performance of the original algorithm in all aspects.

**Figure 9:**
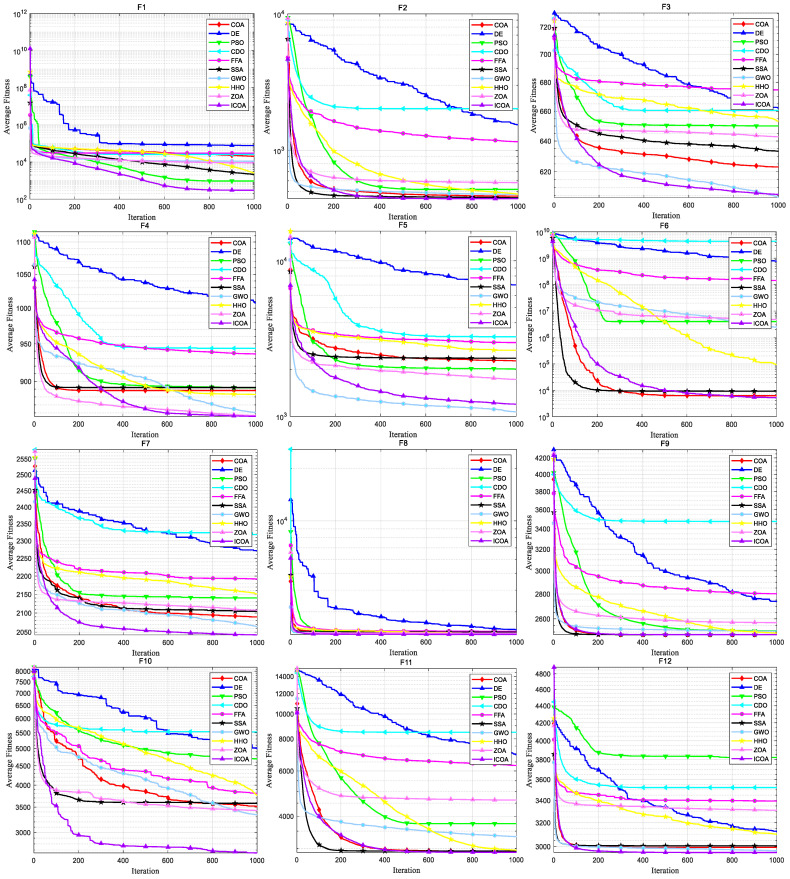
Convergence curves of ICOA and other optimization algorithms for solving CEC 2022 test set.

**Figure 10:**
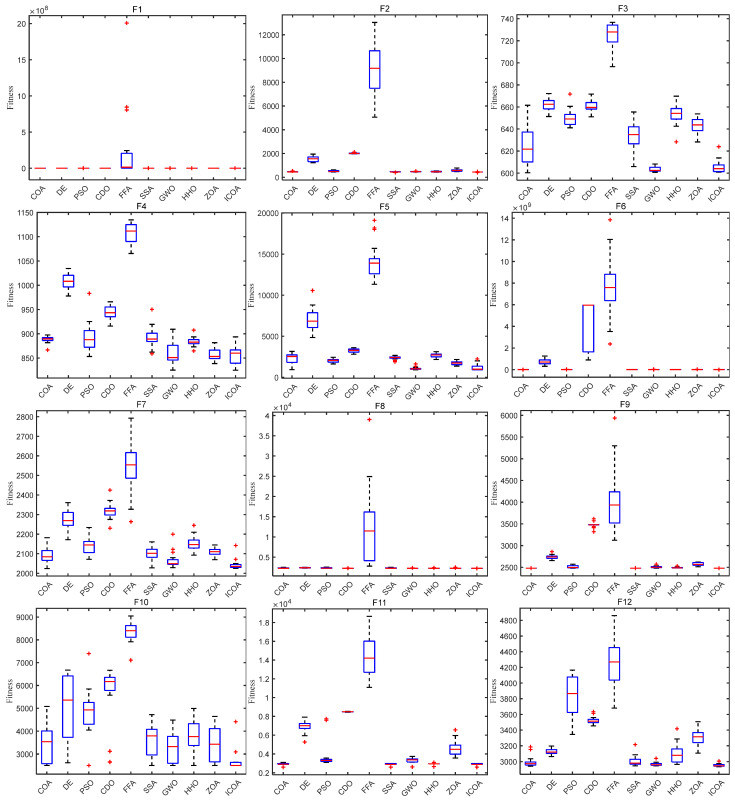
Box plot of ICOA and other optimization algorithms running 20 times for solving CEC 2022 test set.

Figure 9 and Figure 10 show the convergence plot and box plot for the 12 test functions, respectively. As can be seen from Figure 9,the ICOA has a faster convergence speed and higher accuracy on test functions F1, F3, F7, and F10, and converges in the later period. On the test functions F2, F4, F6, F9, F11, and F12, although the convergence speed of the ICOA is not very fast, it is obviously superior to other comparison algorithms in the most accurate calculation. In general, when solving CEC 2022 test functions, the ICOA performs well on 90% of the test functions, and is superior to other comparison algorithms in terms of calculation accuracy and convergence speed. Refer to Figure 10. It can be observed that the abnormal points generated by the ICOA are relatively few. In most of the test functions, the median value of the ICOA is superior. Moreover, on the F3, F5, F7, F11, and F12 test functions, the box area is relatively narrow and the position is lower. In general, the ICOA has high stability and good solution accuracy.

### 4.2. ICOA Compared with the Second Group of Optimization Algorithms

#### 4.2.1. Comparison on CEC 2020 Test Set

In order to further validate the performance of the proposed ICOA, a comparative analysis is conducted with nine other intelligent optimization algorithms on the CEC 2020 test set. (1) Improved classical algorithms include the Gaussian Quantum Particle Swarm Algorithm (GQPSO) [55] and the Adaptive Differential Evolutionary Algorithm (SaDE) [56]. (2) Newly proposed algorithms in the last two years include the Locust Optimization Algorithm (GOA) [57], the Whale Optimization Algorithm (WOA) [58], the Arithmetic Optimization Algorithm (AOA) [59], and the original crayfish algorithm [41]. (3) Improved high-performance algorithms include the Improved Gray Wolf Optimizer (IGWO) [60] and the Improved Sparrow Optimization Algorithm (ISSA) [61]. (4) The Tree Growth Algorithm (TTA) [62] was cited more than 200 times in 2018. The experiments were set for all algorithms with a population size of 50, a dimension of 10, and a maximum number of iterations of 1000. Table 4 shows the initial parameters of all the optimization algorithms used in this section.

Table 5 shows the worst, best, mean, std, and Wilcoxon rank sum test *p*-values obtained by the ICOA and the second group of optimization algorithms after 20 independent runs of the CEC 2020 test set, and the bolded data are the optimal mean and minimum variance of each test function. It can be seen from Table 5 that the ICOA’s optimization ability in ten test functions has been greatly improved compared with the COA. On the test functions F1, F4, F5, F7, and F10, the ICOA has the smallest standard deviation difference when it reaches the minimum fitness average. On F3 and F6, IGWO reaches the optimal fitness value and has a small variance. In terms of average rank and ranking, SaDE, ICOA, IGWO, and WOA perform better because their average rank is less than 5. The ICOA is in the top three on nine test functions with an average rank of 1.6, ranking first. Therefore, the performance order of nine algorithms in solving the CEC2020 test function is ICOA > IGWO > SaDE > WOA > ISSA > AOA > COA > WOA > GOA > GQPSO. According to the statistical results of the Wilcoxon rank sum test in the last row of Table 5 and the ranking of each algorithm, the Wilcoxon rank sum test results of ten optimization algorithms can be obtained as follows: 0/0/10, 1/0/9, 0/0/10, 0/0/10, 0/0/10, 0/0/10, 0/1/9, 0/0/10, and 1/0/9, respectively. It can be seen that the ICOA is superior to the COA in 10 test functions. In summary, the ICOA effectively improves the performance of the original algorithm and shows strong ability in solving the CEC 2020 test set.

**Figure 11:**
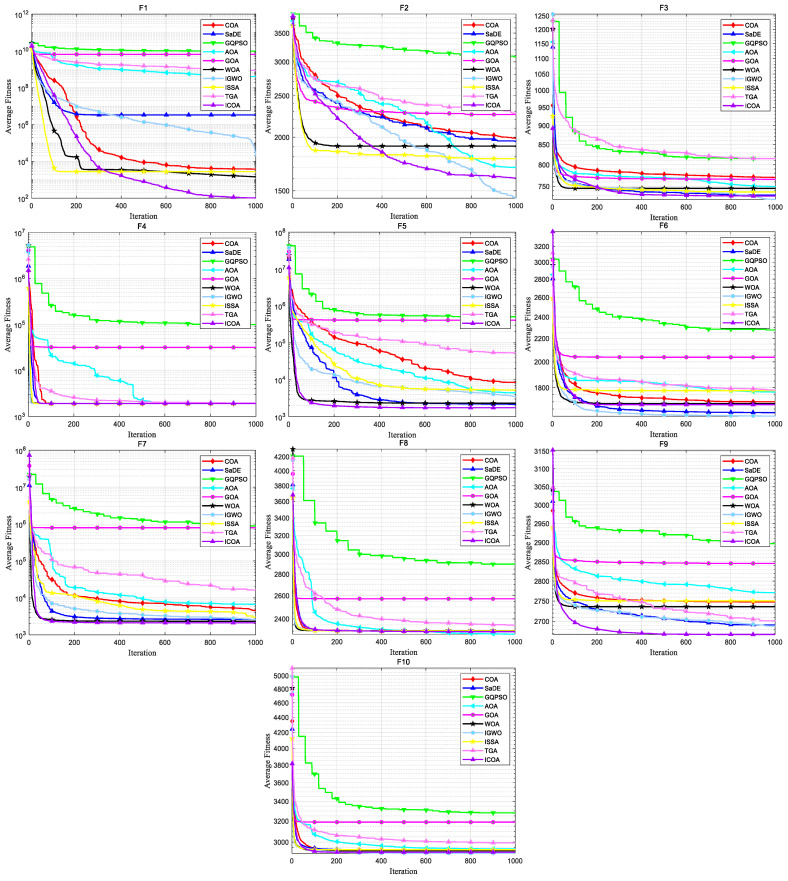
Convergence curves of ICOA and other optimization algorithms for solving CEC 2020 test set.

**Figure 12:**
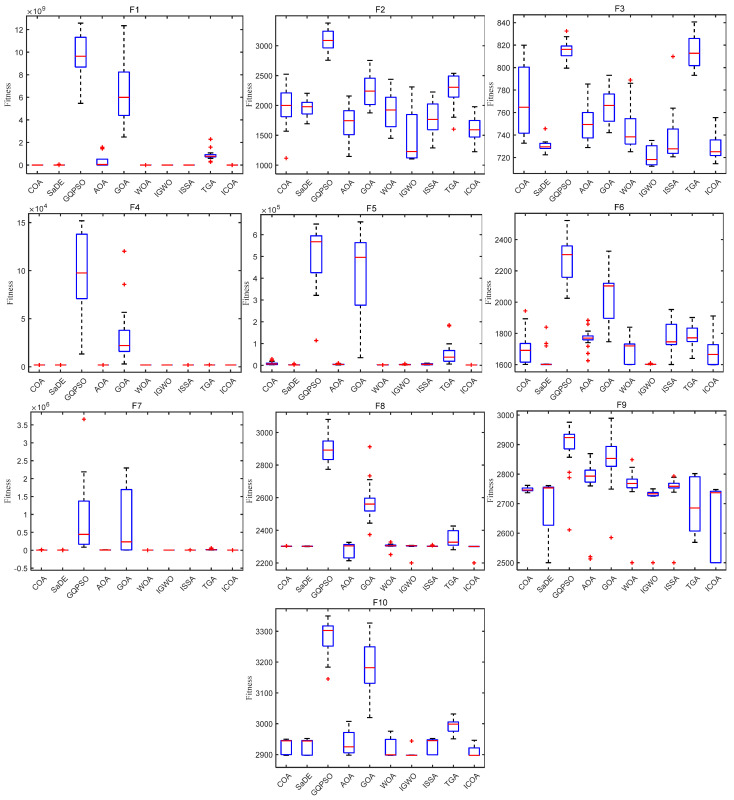
Box plot of ICOA and other optimization algorithms running 20 times for solving CEC 2020 test set.

Figure 11 and Figure 12 show the convergence plot and box plot for the 10 test functions, respectively. As can be seen from Figure 11, on the test functions F4, F5, F7, F8, F9, and F10, the ICOA has a faster convergence speed and higher accuracy, and has been converging in the later stage. When solving CEC 2020 test functions, the ICOA performs well on most test functions, and is superior to other comparison algorithms in calculation accuracy and convergence speed. From Figure 12, it can be seen that the ICOA generates fewer outliers. In most test functions, the median of the ICOA is better, the box area of the F1, F4, F5, F7, and F8 test functions is narrow, and the position is lower. In general, the ICOA has high stability and good solving precision.

#### 4.2.2. Comparison on Test Set CEC 2017

It is more convincing to perform comparison experiments on different test sets, so this section describes comparison experiments on the CEC 2017 test set, and the experiments set the population size of all algorithms to be *pop* = 50, the number of dimensions to be *dim* = 10, and the maximum number of iterations to be iterated *T* = 1000.

Table 6 shows the worst, best, mean, Std, and Wilcoxon rank sum test *p*-values obtained by the ICOA and other optimization algorithms after 20 independent runs of the CEC 2017 test set, and the bolded data are the optimal mean and minimum variance of each test function. As can be seen from Table 6, compared with the COA, the ICOA’s 29 test functions have been greatly improved. On F1, F4, F7, F8, F9, F11, F12, F13, F14, F18, F19, F20, and F29, the ICOA reached the optimal fitness value and the variance was also small. In F15, F17, and F26, although the minimum variance was not reached, the optimal average value was reached. In terms of average rank and ranking, the IGWO, ICOA, SaDE, and WOA perform better because their average rank is less than 5. The ICOA is in the top three on twenty-eight test functions, in the top two on twenty-five test functions, and in first place on fifteen test functions, with an average rank of 1.655, ranking first. According to the statistical results of Wilcoxon rank sum testing in the last row of Table 6, combined with the ranking of each algorithm, the Wilcoxon rank sum test results of nine optimization algorithms can be obtained as follows: 0/0/29, 0/2/27, 0/0/29, 1/1/27, 0/0/29, 1/2/26, 3/2/24, 0/1/28, and 1/1/27. It can be seen that the ICOA is superior to the COA in 29 test functions. In summary, the ICOA effectively improves the performance of the original algorithm and shows strong ability in solving the CEC 2017 test set.

**Figure 13:**
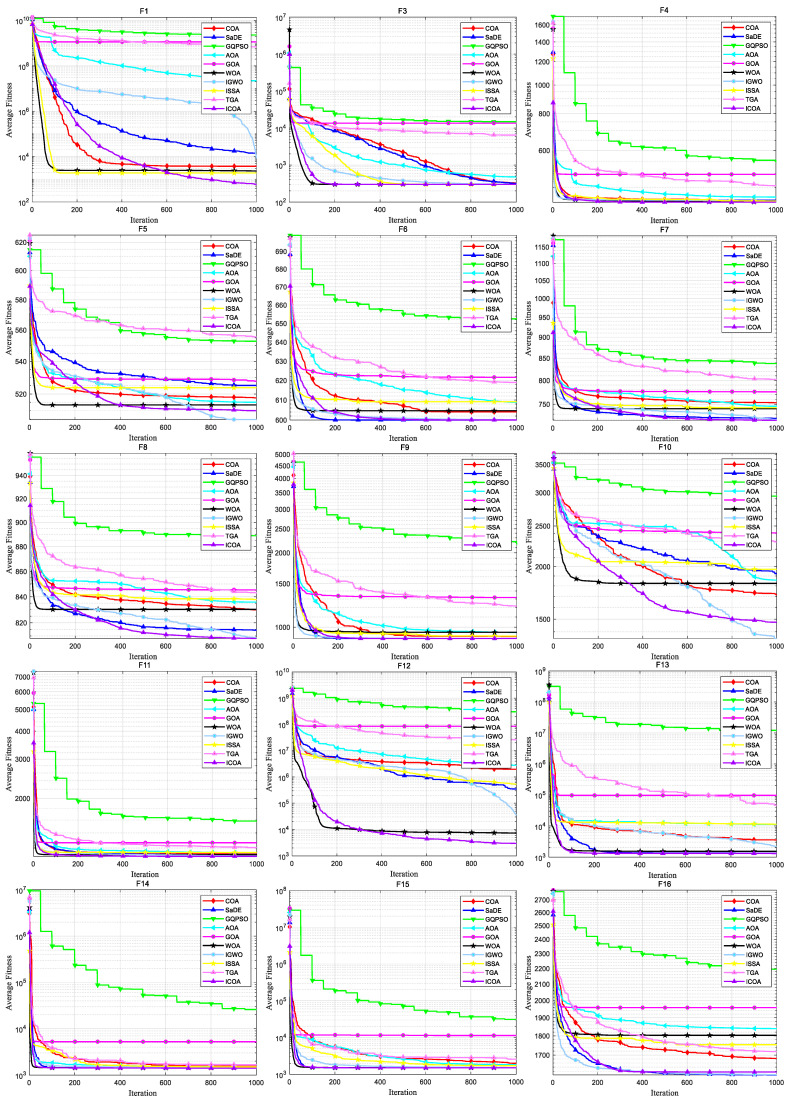

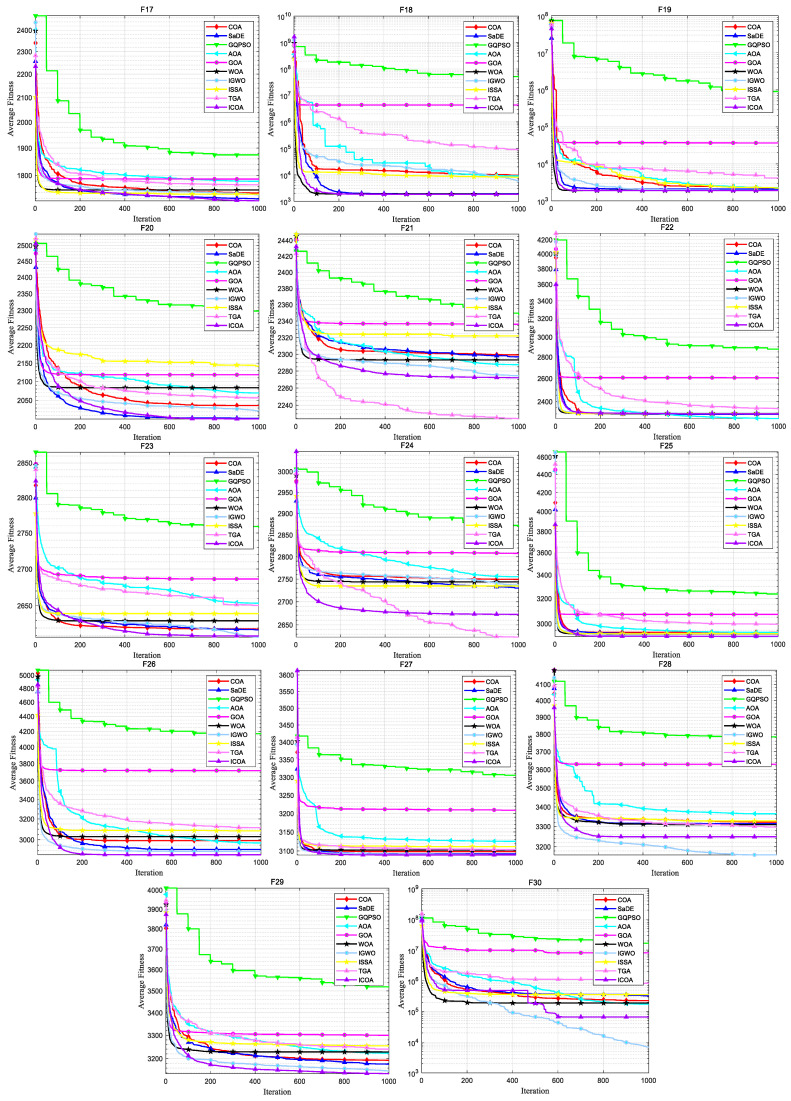
Convergence curve of ICOA and other algorithms for solving 10-dimensional CEC2017 test set.

Figure 13 shows the convergence curve of each algorithm on the CEC2017 test set. It can be visually seen that the COA falls into local optimization and leads to premature convergence on F3, F10, F16, F18, F19, F20, F24, F29, and F30, while the ICOA’s convergence speed and accuracy are greatly improved compared with the COA. And most of the test functions do not stop in the later iteration stage, but continue converging. Comparing the convergence curves of other algorithms, we can see that the ICOA has a faster convergence speed and higher convergence accuracy. Especially in F1, F8, F12, F13, F25, F26, and F29, the ICOA’s advantage is more obvious.

**Figure 14:**
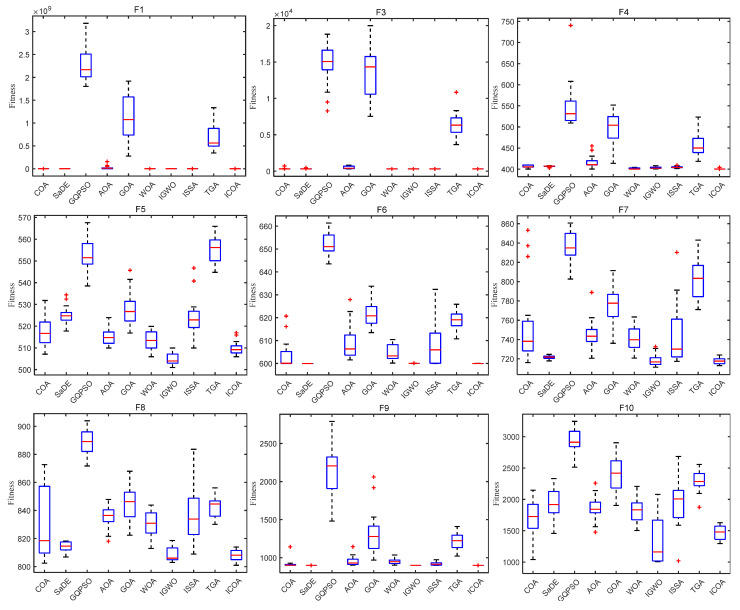

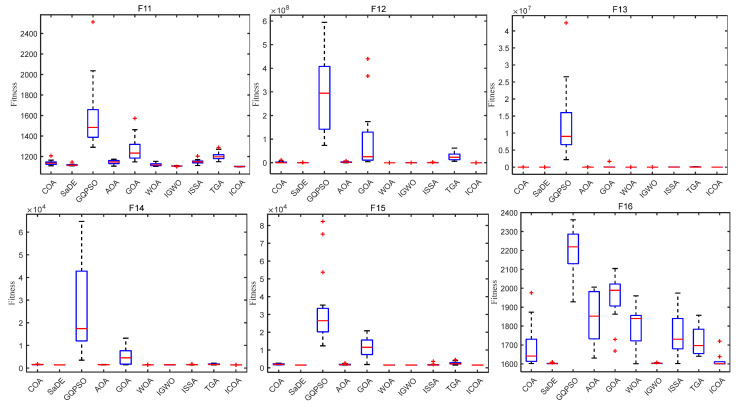

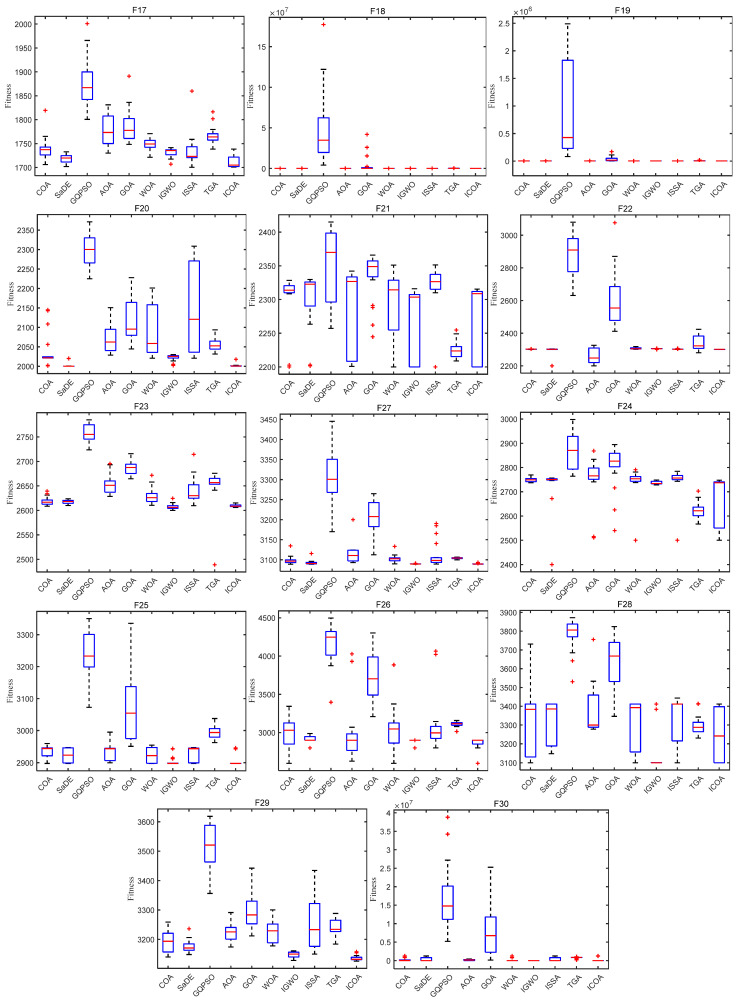
ICOA and other algorithms solving the box diagram of 10-dimensional CEC 2017 test set.

Figure 14 shows the box diagram of each algorithm on the CEC 2017 test function. As can be seen that on most test functions, the ICOA has a narrow box type, low position, and small median, which indicates that the solution obtained by the ICOA has high consistency and high precision. In addition, the ICOA has fewer outliers on 29 test functions, which indicates that the algorithm is less affected by policy randomness during operation. It can be seen from the box diagram that the ICOA is superior to other comparison algorithms in terms of stability and accuracy.

## 5. ICOA Solves All Kinds of Optimization Problems

### 5.1. ICOA Solves Engineering Optimization Problems

This section aims to assess the efficiency of the ICOA in solving real-world engineering optimization problems by comparing it with other high-performance algorithms. For the six engineering problems involved, the penalty function is first used to transform these constrained problems into unconstrained problems before comparing them. We set the population size, maximum number of iterations, and number of independent runs for all optimization algorithms to 40, 1000, and 30, respectively. The data in bold in this section are all the relevant optimal values that the algorithm solves for each problem.

#### 5.1.1. Speed Reducer Design Problem

**Figure 15:**
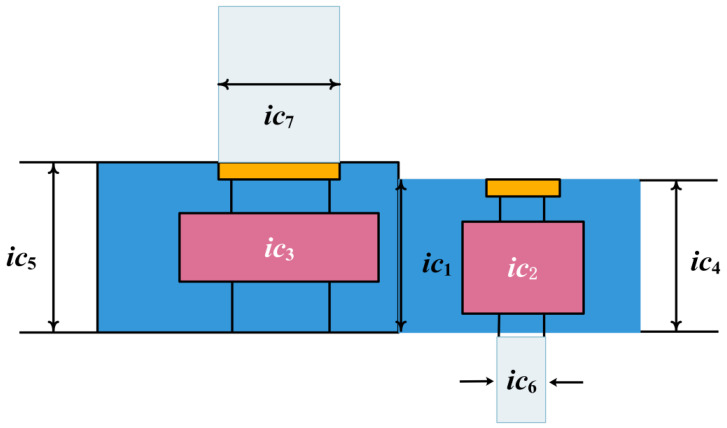
Schematic design of reducer.

The objective of the problem is to obtain the minimum mass under four design constraints; Figure 15 shows a schematic diagram of this problem, and the mathematical models for these four constraints are as follows:

Set the following:

(32) $$IC=[ic_{1}\;ic_{2}\;ic_{3}\;ic_{4}\;ic_{5}\;ic_{6}\;ic_{7}]\;$$

Minimize

(33) $$\begin{array}{l} f(IC)=0.7854ic_{1}ic_{2}^{2}(3.3333ic_{3}^{2}+14.9334ic_{3}-43.0934)-1.508ic_{1}(ic_{6}^{2}+ic_{7}^{2})\\ +7.4777ic_{6}^{3}+ic_{7}^{3}+0.7854ic_{4}ic_{6}^{2}+ic_{5}ic_{7}^{2} \end{array}$$

Subject it to

(34) $$\left\{\begin{array}{l} g_{1}(IC)=\frac{27}{ic_{1}ic_{2}^{2}ic_{3}}-1\leq 0,g_{2}(IC)=\frac{397.5}{ic_{1}ic_{2}^{2}ic_{3}^{2}}-1\leq 0,\\ g_{3}(IC)=\frac{1.93ic_{4}^{3}}{ic_{2}ic_{3}ic_{6}^{4}}-1\leq 0,g_{4}(IC)=\frac{1.93ic_{5}^{3}}{ic_{2}ic_{3}ic_{6}^{4}}-1\leq 0,\\ g_{5}(IC)=\frac{1}{100ic_{6}^{3}}\sqrt{{\left(\frac{745ic_{4}}{ic_{2}ic_{3}}\right)}^{2}+16.9\times 10^{6}}-1\leq 0,\\ g_{6}(IC)=\frac{1}{85ic_{7}^{3}}\sqrt{{\left(\frac{745ic_{5}}{ic_{2}ic_{3}}\right)}^{2}+16.9\times 10^{6}}-1\leq 0,g_{7}(IC)=\frac{ic_{2}ic_{3}}{40}-1\leq 0,\\ g_{8}(IC)=\frac{5ic_{2}}{ic_{1}}-1\leq 0,g_{9}(IC)=\frac{ic_{1}}{12ic_{2}}-1\leq 0,\\ g_{10}(IC)=\frac{1.5ic_{6}+1.9}{ic_{4}}-1\leq 0,g_{11}(IC)=\frac{1.1ic_{7}+1.9}{ic_{5}}-1\leq 0 \end{array}\right.$$

The boundaries are as follows:

(35) $$\left\{\begin{array}{l} 2.6\leq ic_{1}\leq 3.6,\;0.7\leq ic_{2}\leq 0.8,\;17\leq ic_{3}\leq 28,\;7.3\leq ic_{4}\leq 8.3,\\ 7.3\leq ic_{5}\leq 8.3,\;2.9\leq i\;c_{6}\leq 3.9,\;5\leq ic_{7}\leq 5.5. \end{array}\right.$$

Table 7 shows the optimal value and corresponding design variables obtained by the ICOA and other optimization algorithms to solve the reducer design problem. These comparison algorithms are GWO [51], HHO [20], DO [63], WFO [64], GOA, SSA, FFA [49], and AOA. Further, Table 8 gives the statistical results of the design problems of the reducer. It can be seen from the table that the ICOA can obtain an optimal cost of 2996.348165, which indicates that the ICOA can obtain the best result in solving the design problem of the reducer.

#### 5.1.2. Hydrodynamic Thrust Bearing Design Problems

**Figure 16:**
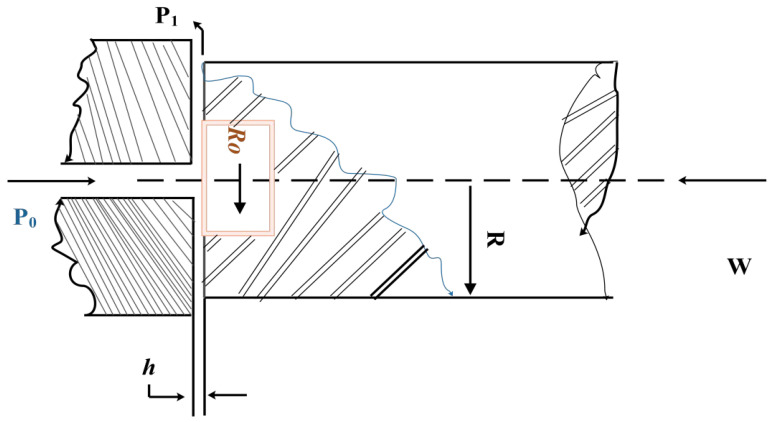
Schematic diagram of hydrostatic thrust bearing.

The primary aim of this design problem is to minimize the power loss in the bearings [65]. Figure 16 illustrates this issue. This objective is mathematically expressed as follows:

The min is the following:

(36) $$f(coa)=\frac{qp_{0}}{0.7}+E_{f}$$

Subject to

(37) $$\left\{\begin{array}{l} c_{1}(coa)=1000-p_{0}\leq 0,c_{2}(coa)=W-101000\leq 0,\\ c_{3}(coa)=5000-\frac{W}{\pi (r^{2}-r_{0}^{2})}\leq 0,c_{4}(coa)=50-p_{0}\leq 0,\\ c_{5}(coa)=0.001-\frac{0.0307}{386.4p_{0}}\left(\frac{q}{2\pi rh}\right)\leq 0,c_{6}(coa)=r-r_{0}\leq 0,\\ c_{7}(coa)=h-0.001\leq 0. \end{array}\right.$$

where

(38) $$\left\{\begin{array}{l} W=\frac{\pi P_{0}}{2}\frac{r^{2}-R_{0}^{2}}{\ln\left(\frac{r}{r_{0}}\right)},P_{0}=\frac{6\mu q}{\pi h^{3}}\ln\left(\frac{r}{r_{0}}\right),\\ E_{f}=9336q\times 0.0307\times 0.5k,\\ k=2(10^{p}-559.7),\\ h={\left(\frac{2\pi \times 750}{60}\right)}^{2}\frac{2\pi \mu }{E_{f}}\left(\frac{r^{4}}{4}-\frac{r_{0}^{4}}{4}\right) \end{array}\right.$$

The boundaries are as follows:

(39) $$\left\{\begin{array}{l} 1\leq r\leq 16,\;1\leq r_{0}\leq 16,\\ 1\times 10^{-6}\leq \mu \leq 16\times 10^{-6},\;1\leq q\leq 16. \end{array}\right.$$

Table 9 presents the comparison of the optimal values and design variables achieved through the ICOA, as well as several other optimization algorithms, in addressing the problem of hydrostatic thrust bearing. These algorithms include GWO [51], HHO [20], DO, WFO, GOA, SSA, FFA [49], and AOA [59]. The results of the mathematical calculations for this problem are further given in Table 10. From the table, we can observe that the ICOA achieves an optimal cost of 2996.348165 for this design problem. This result highlights the superiority of the ICOA over other algorithms in obtaining the optimal solution for this specific problem.

#### 5.1.3. Welded Beam Design Problem

**Figure 17:**
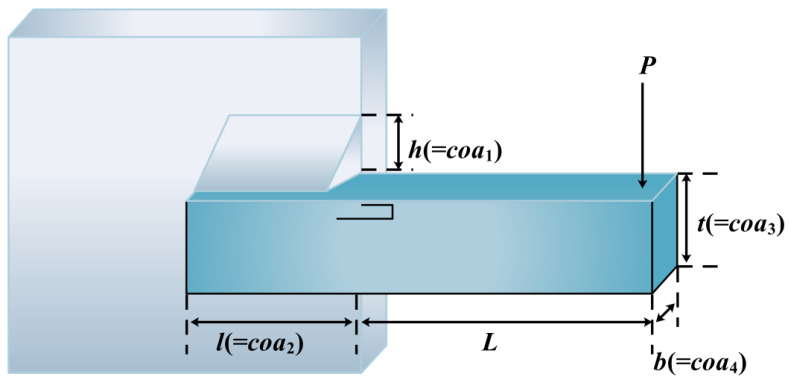
Schematic diagram of welded beam design issues.

The main objective of this specific problem is to formulate a design for a welded beam that minimizes the total cost [66]. Figure 17 is a simulation of this problem. The problem can be mathematically represented as follows:

Minimize

(40) $$f(COA)=0.04811coa_{3}coa_{4}(coa_{2}+14)+1.10471coa_{1}^{2}coa_{2}$$

Subject it to:

(41) $$\left\{\begin{array}{l} c_{1}(COA)=coa_{1}-coa_{4}\leq 0,\;coa_{2}(COA)=\delta (COA)-\delta_{\max}\leq 0,\\ c_{3}(COA)=P\leq P_{c}(COA),\;c_{4}(COA)=\tau_{\max}\geq \tau (COA),\\ \;c_{5}(COA)=\sigma (COA)-\sigma_{\max}\leq 0. \end{array}\right.$$

where

(42) $$\left\{\begin{array}{l} \tau =\sqrt{\tau^{\prime 2}+\tau^{\prime \prime 2}+2\tau^{\prime }\tau^{\prime \prime }\frac{coa_{2}}{2R}},\;\tau^{\prime \prime }=\frac{Rm}{J},\;\tau^{\prime }=\frac{P}{\sqrt{2}coa_{2}coa_{1}},m=P\left(\frac{coa_{2}}{2}+L\right),\\ R=\sqrt{\frac{coa_{2}^{2}}{4}+{\left(\frac{coa_{1}+coa_{3}}{2}\right)}^{2}},\;J=2\left(\left(\frac{coa_{2}^{2}}{4}+{\left(\frac{coa_{1}+coa_{3}}{2}\right)}^{2}\right)\sqrt{2}coa_{1}coa_{2}\right)\\ \sigma (COA)=\frac{6PL}{coa_{4}coa_{3}^{2}},\;\sigma (COA)=\frac{6PL^{3}}{Ecoa_{3}^{2}coa_{4}},\;\\ P_{c}(COA)=\frac{4.013Ecoa_{3}coa_{4}^{3}}{6L^{2}}\left(1-\frac{coa_{3}}{2L}\sqrt{\frac{E}{4G}}\right). \end{array}\right.$$

(43) $$\left\{\begin{array}{l} l=14IN,\;p=6000lb,\;e=30.10^{6}pi,\;s_{\max}=30000pi,\\ q_{\max}=13600psi,\;g=12.10^{6}pi,\;t_{\max}=0.25IN. \end{array}\right.$$

The boundaries are as follows:

(44) $$0.1\leq coa_{3},coa_{2}\leq 10,\;0.1\leq coa_{4}\leq 2,\;0.125\leq coa_{1}\leq 2.$$

As shown in Table 11, the optimal values and the corresponding four design variables solved by the ICOA are compared with the results obtained by COA [41], GWO [51], HHO [20], SO [18], DO [63], WFO [64], GOA, SSA, ISSA, FFA [49], and AOA [59]. The statistical results of each algorithm for solving this problem are given in Table 12. By examining the results, it becomes evident that the ICOA consistently outperforms other algorithms in terms of all metrics, indicating its capability to offer higher-quality and more stable solutions for this problem. This underscores the robust adaptability of the ICOA.

#### 5.1.4. Robot Gripper Design Optimization Problem

**Figure 18:**
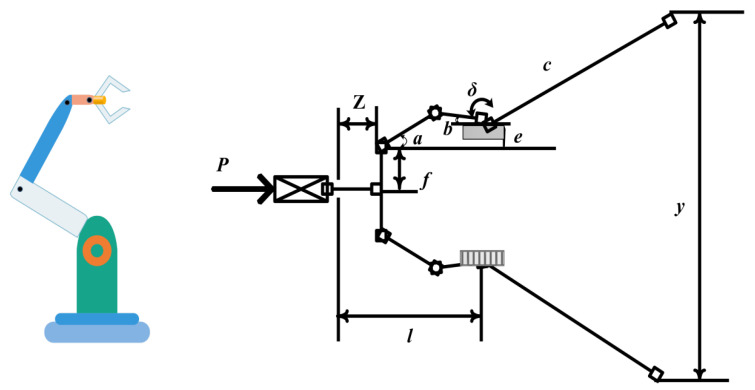
Schematic of the robot gripper arm design problem.

This design problem primarily aims to address the range between the maximum and minimum values generated by the clamping arm of the robot [67]. Figure 18 is a simulation of this problem. The problem can be formulated as follows:

Set

***IC*** = (*ic*_1_,*ic*_2_,*ic*_3_,*ic*_4_,*ic*_5_,*ic*_6_,*ic*_7_) (45)

Minimize

(46) $$f(IC)=-\underset{C}{\min}F_{k}(IC,C)+\max F_{k}(IC,C)$$

Subject it to

(47) $$\left\{\begin{array}{l} c_{1}(IC)=-Y_{\min}+y(IC,C_{\max})\leq 0,\;c_{2}(IC)=-y(IC,C_{\max})\leq 0,\\ c_{3}(IC)=Y_{\max}-y(IC,0)\leq 0,c_{4}(IC)=y(IC,0)-Y_{G}\leq 0,\\ c_{5}(IC)=l^{2}+e^{2}-{\left(a+b\right)}^{2}\leq 0,c_{6}(IC)=b^{2}-{\left(a-e\right)}^{2}-{\left(l-C_{\max}\right)}^{2}\leq 0\\ c_{7}(IC)=C_{\max}-l\leq 0, \end{array}\right.$$

where

(48) $$\left\{\begin{array}{l} \alpha =\cos_{-1}\left(\frac{i{c_{1}}^{2}+g^{2}+i{c_{5}}^{2}}{2ic_{1}g}\right)+\phi ,g=\sqrt{i{c_{4}}^{2}+{\left(C-ic_{6}\right)}^{2}},\;\\ \beta =\cos_{-1}\left(\frac{i{c_{5}}^{2}+g^{2}-i{c_{1}}^{2}}{2ic_{1}g}\right)-\phi ,\phi =\tan_{-1}\left(\frac{ic_{4}}{ic_{6}-C}\right),\\ y(IC,C)=2(ic_{2}+ic_{4}+ic_{3}\sin(\beta +ic_{7})),F_{k}=\frac{Pic_{5}\sin(\alpha +\beta )}{2ic_{3}\cos(\alpha )},\\ Y_{\min}=50,Y_{\max}=100,Y_{G}=150,C_{\max}=100,P=100. \end{array}\right.$$

The boundaries are as follows:

(49) $$\left\{\begin{array}{l} 0\leq ic_{4}\leq 50,\;100\leq ic_{3}\leq 200,\;10\leq ic_{2},ic_{1},\\ ic_{5}\leq 150,\;1\leq ic_{7}\leq 3.14,\;100\leq ic_{6}\leq 300. \end{array}\right.$$

Table 13 shows the optimal value and optimal corresponding variable obtained by the ICOA and other optimization algorithms for solving the robot arm design problem. These comparison algorithms are as follows: COA [41], SCA [68], AO [69], BWO [70], DO [63], WFO [64], GOA, SSA, RSA [71], FFA [49], GRO [72], and AOA [59]. Table 14 further gives the statistical results of the robot clamp arm design problems. As can be seen from the table, the ICOA can obtain the optimal cost of 7.2740693811E−17, which is better than COA.

#### 5.1.5. Cantilever Beam Design Issues

**Figure 19:**
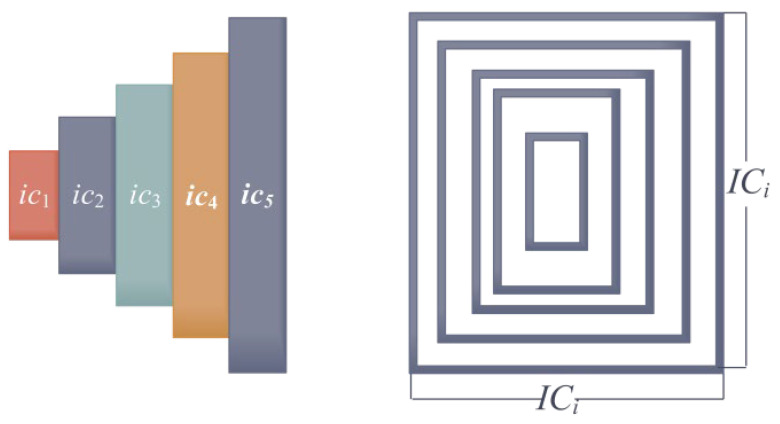
Schematic of cantilever beam design.

The engineering problem at hand is relatively straightforward, aiming to minimize the weight of a cantilever beam by utilizing five variables [73]. Figure 19 is a simulation of this problem.

Minimize

(50) $$IC=[ic_{1}\;ic_{2}\;ic_{3}\;ic_{4}\;ic_{5}]$$

(51) $$f(IC)=0.6224(ic_{1}+ic_{2}+ic_{3}+ic_{4}+ic_{5})$$

Subject it to

(52) $$c(IC)=\frac{60}{ic_{1}^{3}}+\frac{27}{ic_{2}^{3}}+\frac{19}{ic_{3}^{3}}+\frac{7}{ic_{4}^{3}}+\frac{1}{ic_{5}^{3}}-1\leq 0$$

The boundaries are as follows:

(53) $$0.01\leq ic_{1},ic_{2},ic_{3},ic_{4},ic_{5}\leq 100$$

As shown in Table 15, the ICOA was used to solve the cantilever beam design problem, and the optimal values and the corresponding four design variables solved by the ICOA were compared with the results obtained by COA [41], SCA, AO, BWO, WFO, GOA, SSA, FFA [49], and AOA [59]. The numerical results of each algorithm solving this problem alone are given in Table 16. By analyzing the results, it becomes apparent that the ICOA consistently produces smaller values for all the indicators. This outcome indicates that the ICOA is capable of providing higher-quality and more stable solutions for this problem. This highlights the strong performance of the ICOA.

#### 5.1.6. Heat Exchanger Design Issues

This design application is taken from Hawk and Hitkowski [74]. It features a linear objective function whose minimization is bounded by six inequalities (three of which are non-convex). All eight variables are bounded. The parameters are slightly modified, and the mathematical model is as follows [74]:

Minimize

(54) $$f(IC)=ic_{1}+ic_{2}+ic_{3}.$$

Subject it to

(55) $$\left\{\begin{array}{l} c_{1}(IC)=0.0025(ic_{4}+ic_{6})-1\leq 0,\;c_{2}(IC)=0.0025(ic_{5}+ic_{7}-ic_{4})-1\leq 0,\\ c_{3}(IC)=0.01(ic_{8}-ic_{5})-1\leq 0,\\ c_{4}(IC)=-ic_{1}ic_{6}+833.33252ic_{4}+100ic_{1}-63333.333\leq 0,\\ c_{5}(IC)=-ic_{2}ic_{7}+1250ic_{5}+ic_{2}ic_{4}-1250ic_{4},\\ c_{6}(IC)=-ic_{3}ic_{8}+ic_{3}ic_{5}-2500ic_{5}+1250000\leq 0. \end{array}\right.$$

The boundaries are as follows:

(56) $$\left\{\begin{array}{l} 100\leq ic_{1}\leq 10000,\;1000\leq ic_{2}\leq 10000,\;\\ 1000\leq ic_{3}\leq 10000,\;10\leq ic_{4},ic_{5},ic_{6},ic_{7},ic_{8}\leq 1000. \end{array}\right.$$

As shown in Table 17, the ICOA is used to solve heat exchanger design problems. The optimal value of the ICOA solution and the corresponding eight variables were compared with the results obtained by ISSA, GWO [51], AOA [59], AO, COA [41], HHO [20], WFO, GOA, BWO, DO, SO [18], GRO. Table 18 shows the numerical results of each algorithm to solve this problem. It can be seen that the ICOA solves all the indicators relatively quickly. This shows that the ICOA provides more stable and more accurate solutions for heat exchanger design problems.

### 5.2. ICOA Solves Constrained Optimization Problems

This section involves the utilization of a set of mathematical functions, encompassing a total of 30 functions. To narrow down the scope, 20 functions were specifically examined within the context of small-dimensional problems. Additionally, the remaining 10 functions were explored in relation to large-dimensional problems. Notably, these functions encompass six distinct forms. Each section provides detailed and comprehensive information pertaining to the specific functions under examination. Detailed information on the functions considered is available online at https://www.sfu.ca/~ssurjano/ (accessed on 14 April 2025). In this section, thirteen comparison algorithms are selected from COA [41], AO, AOA [59], DO, HHO [20], GWO, SaDE, SO [18], GRO, ISSA, BWO, WFO [64], and GOA.

#### 5.2.1. Low-Dimensional Problems

**Figure 20:**
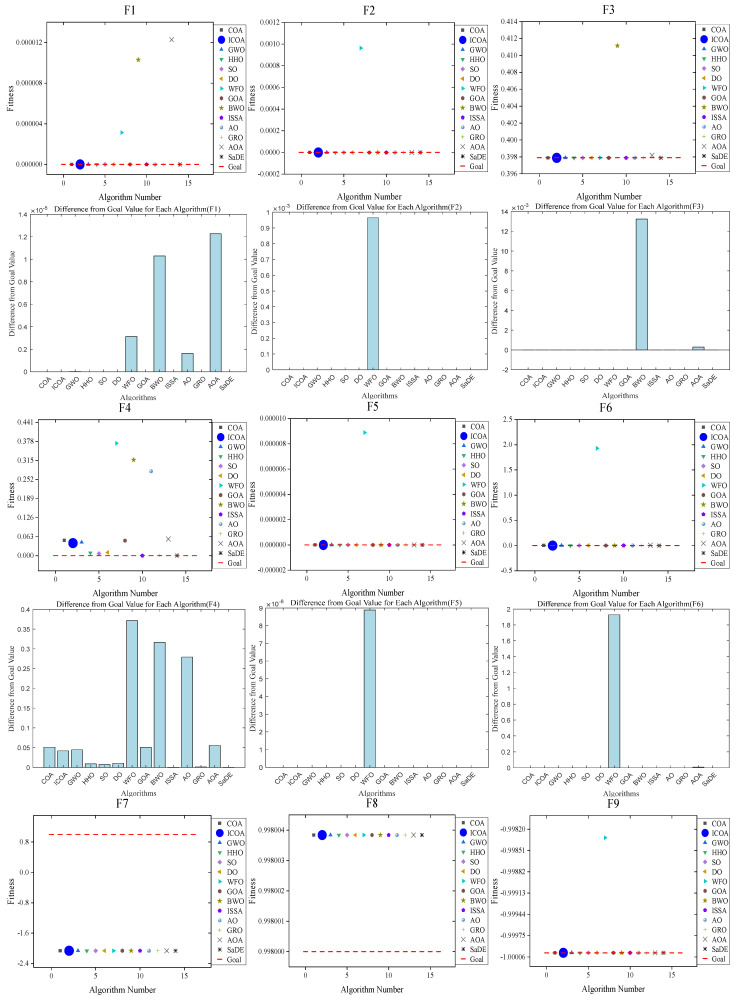

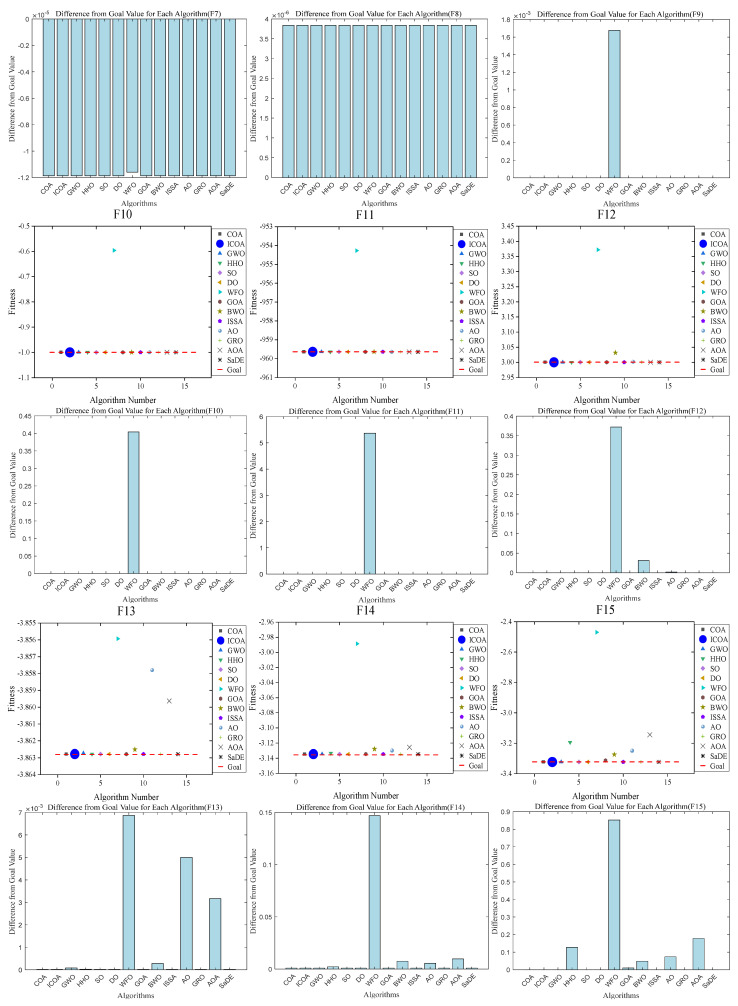

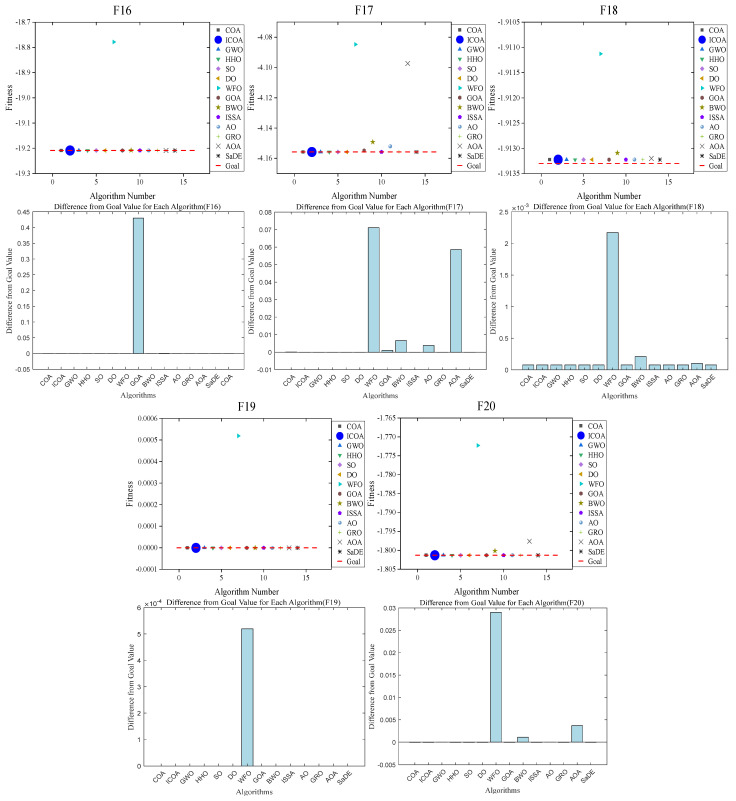
The comparison chart of the optimal values of the ICOA on F1–F20 with other comparative algorithms, as well as the histogram of the differences in the results of each algorithm.

In this section, the ICOA and other comparative algorithms are utilized to evaluate 20 benchmark functions with dimensions ranging from 2 to 6 (F1 to F20). Additionally, the summary of the optimal values obtained by each algorithm and the target values of the benchmark functions is presented in Table 19 and Figure 20. The results show that the proposed ICOA outperforms the original algorithm and most of the comparative algorithms in terms of optimization and providing values close to the optimal response of the target.

Table 19 shows that on low-dimensional problems, the ICOA can achieve optimal results on F7~F14 and F16~F20, which are closer to the required objective values. And on 15 low-dimensional problems, the ICOA solution has better results than the original algorithm solution, thus verifying that the improved crayfish algorithm is more accurate. Figure 20 gives a comparison plot of the optimal fitness values of the ICOA with the other 12 intelligent optimization algorithms on the low-dimensional problem, as well as a histogram of the difference values of each algorithm with respect to the objective value of each function. The comparison chart shows that ICOA works best on F1, F2, F3, F5, F6, and F9~F20. The histogram clearly shows that the ICOA is either the same or closer to the objective value on each function compared to the original algorithm. This shows that the ICOA is more accurate than the original algorithm when dealing with low-dimensional math problems.

#### 5.2.2. Higher Dimensional Problems

In order to evaluate the performance of the algorithms on large-scale problems, 10 benchmark functions (F21 to F30) have been considered. The dimensions studied in this section are 30, 100, 500, and 1000. They were executed 30 times for each problem, algorithm, and dimension. The results show that the proposed algorithm has good performance in estimating the optimal response for large-scale problems. Table 20, Table 21, Table 22, Table 23 and Table 24 provide the results of all the algorithms implemented in this section.

From Table 20, we can see that the ICOA is still suitable for some high-dimensional problems and achieves the best results. Functions F21 to F22 demonstrate cases where the optimal value obtained by the ICOA outperforms the original algorithm, providing evidence that the ICOA is more effective in solving large-scale problems.

Table 21, Table 22 and Table 23 present the optimal solutions obtained by the ICOA, as well as other comparative algorithms, on dimensions of 100, 500, and 1000 for the functions F21 to F30. In particular, it can be seen that for functions F21, F23, F26, and F28, the ICOA produces the best results, while for functions F24, F25, F29, and F30, the ICOA achieves superior optimal values compared to the original algorithm. This underscores the enhanced efficiency and accuracy of the ICOA when solving large-scale problems. Overall, the ICOA offers valuable advantages and increased accuracy in tackling large-scale problem solving.

**Figure 21:**
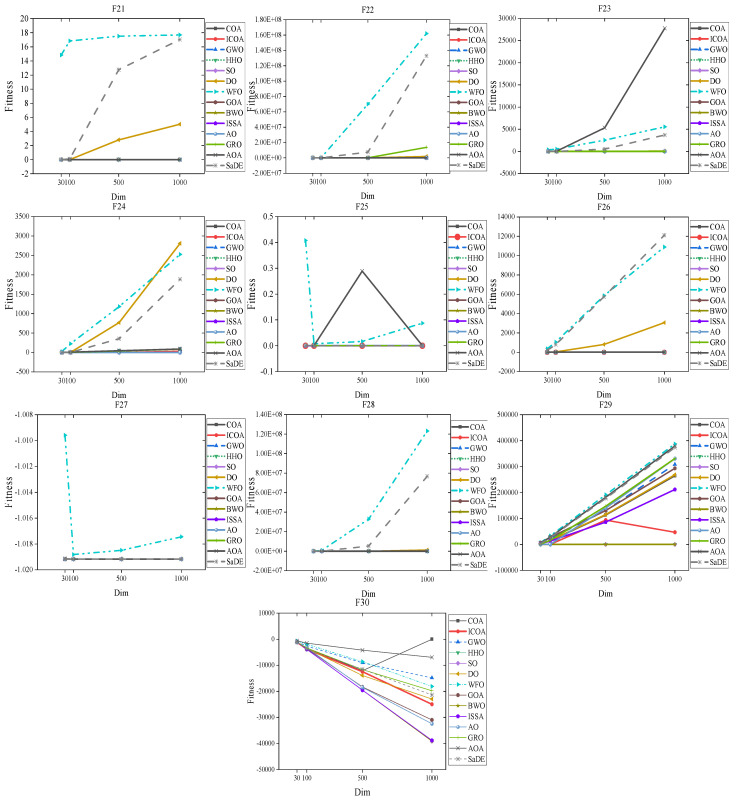
Comparison chart of the optimal fitness values of F21~F30 ICOA with other algorithms in 30, 100, 500, and 1000 dimensions.

Figure 21 gives the optimal fitness value plot of the ICOA with other intelligent optimization algorithms on high-dimensional problems, and 30, 100, 500, and 1000 dimensions are chosen to better illustrate the ability of the ICOA to deal with high-dimensional problems. On F21~F28, the ICOA achieves optimal results on all four dimensions, which shows that the ICOA has a strong advantage on high-dimensional problems. And on F21~F29, the ICOA is better than the original algorithm’s optimization results, which shows that the ICOA is more advantageous in solving high-dimensional problems and has higher accuracy than the original algorithm.

### 5.3. ICOA Solving the NP-Hard Problem

In this subsection, our goal is to examine the extensibility and adaptability of the ICOA to more challenging problem fields, in general, and, in particular,, mixed-integer affine problems. To evaluate the performance of the ICOA on complex problems, we will utilize it in solving two representative mixed-integer nonlinear problems. By comparing the results with other algorithms known for their good performance, we can ascertain the effectiveness of the ICOA in tackling such challenges. These algorithms are SaDE, AO, GOA, AOA, DO, HHO [20], GWO, and COA [41]. Each problem experiment setup parameter was set according to the problem. In our experimental setup, we have established the number of iterations to be *T* = 200, alongside a population size of *N* = 50.

A. NP1. logistics distribution [75]

Logistics distribution has been a significant factor impeding the development of the logistics industry in recent times. Especially for dairy enterprises, cold chain distribution is a very critical link. Coordination and optimization of multiple variables are required in cold storage, transportation, etc., to achieve the purposes of cost reduction and efficiency improvement.

To address this problem, the ICOA is designed for the vehicle optimization of cold chain distribution, which is verified to have the advantages of short solution time, fast convergence, and high solution accuracy by comparing with other algorithms.

**Figure 22:**
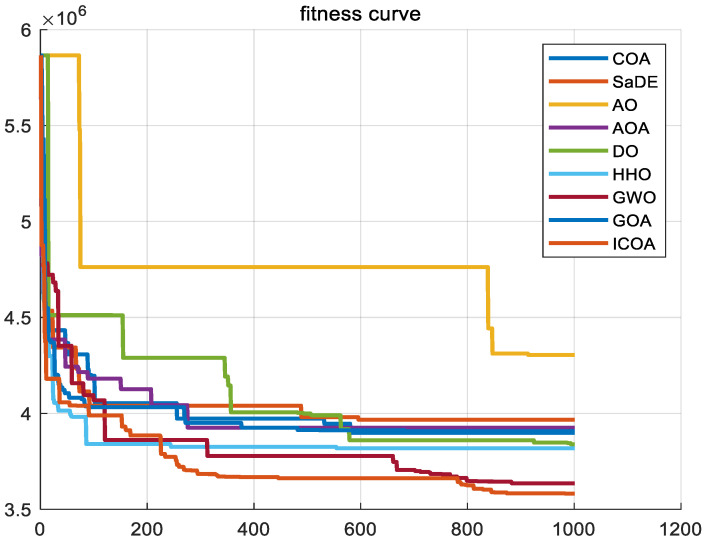
Convergence curves of ICOA and other comparison algorithms in logistics distribution problem.

**Figure 23:**
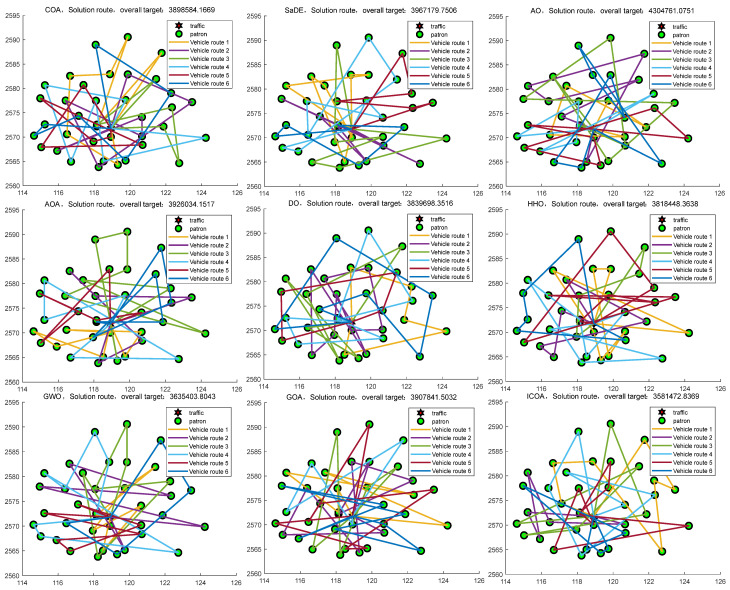
Comparison chart of ICOA and other comparative algorithms for vehicle scheduling optimization.

Figure 22 gives the convergence plot of the algorithms of the ICOA and other algorithms in finding the shortest path to the ball. The ICOA clearly achieves the best results, indicating that ICOA solves this problem with high accuracy. Figure 23 illustrates that the ICOA achieves optimal vehicle scheduling, minimizing the cost to the organization. It shows that the ICOA is more advantageous in solving the problem of vehicle scheduling optimization.

Table 24 gives the optimal values as well as the mean and variance when the ICOA solves NP1. The bolded data are the optimal values for each metric. Taking a look at Table 24, it is evident that the ICOA performs better than other methods in terms of the optimal value, mean, and variance when solving this problem. This observation supports the notion that the ICOA delivers superior quality and stability when solving the problem at hand, indicating its high accuracy and reliability.

B. NP2. TSP issues [75]

Solving the TSP focuses on determining the shortest path by selecting the smallest distance among all available paths. Therefore, we apply the proposed ICOA to this problem to find the shortest path.

**Figure 24:**
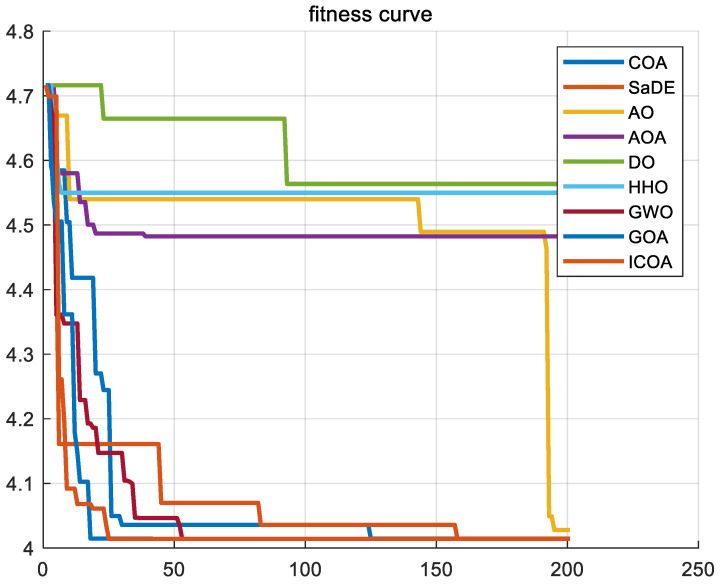
Convergence curves of ICOA and other comparison algorithms in TSP.

**Figure 25:**
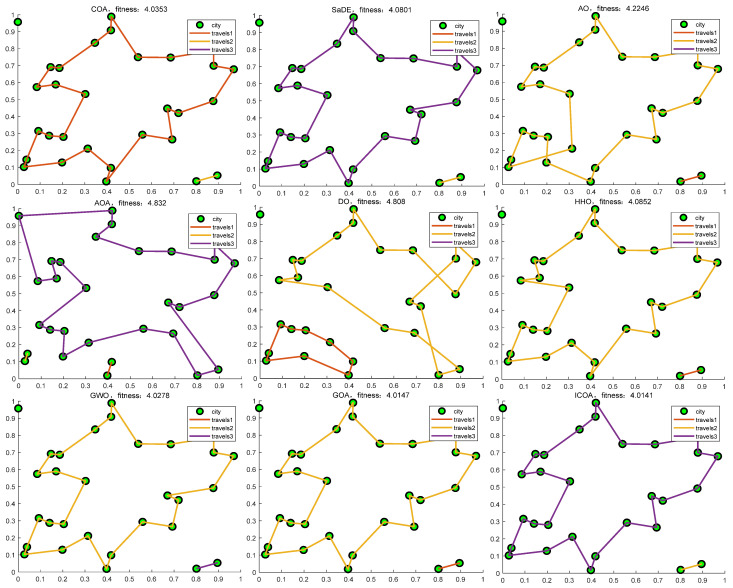
The shortest path graph of the ICOA compared with other contrast algorithms.

By conducting an analysis of Figure 24 and Figure 25, it becomes evident that the ICOA outperforms other algorithms in successfully solving the traveling salesman problem (TSP). It consistently obtains the optimal solution, namely the shortest path, surpassing the performance of alternative algorithms. This shows that the ICOA has a greater advantage than other algorithms in solving the TSP, thus indicating that the ICOA has a good ability to solve the TSP.

Table 25 shows the optimal value, average value, and variance when the ICOA solves the TSP. The bolded data are the optimal values under each index. Table 25 shows that the ICOA has the lowest optimal value and the lowest average value when solving this problem. The ICOA can give a better solution to solve this problem, which shows that the ICOA has a higher precision.

## 6. Discussion

Compared with other classical optimization methods, the ICOA has better optimization performance. Based on three test sets, six engineering problems, high- and low-dimensional mathematical problems, and NP problems, combined with a hypersonic missile path planning verification experiment, the implementation of the ICOA is discussed in detail. On the test set CEC 2020, the ICOA ranked first among all eight optimization algorithms, indicating that the addition of four strategies greatly improved the performance of the COA. On the new test set, CEC 2022, the ICOA ranked second in two test functions, but first in the remaining ten test functions, indicating that the proposed ICOA is equally adaptable and effective in the new problem. To test the powerful performance of the ICOA, we re-selected nine additional optimization algorithms and tested a variety of test functions in the CEC2017 test set. The results show that the ICOA ranks first in fourteen test functions and in the top three in twenty-eight test functions, accounting for 95.6% of the top three, with a ranking value of 1.6. This comprehensively demonstrates that the proposed ICOA represents a substantial improvement to the development and exploration capabilities of the original COA. Finally, the proposed ICOA is optimized for both high- and low-dimensional mathematical functions and NP problems. It is proved that the ICOA has great advantages in high-dimensional problems. These tests verify the effectiveness and reliability of the ICOA in engineering optimization.

## 7. Conclusions and Future Work

The main research focus of this paper is as follows. (1) An improved crayfish algorithm (ICOA) based on the elite chaotic difference strategy, the difference variance strategy, the Levy flight strategy, and the multi-strategy of the dimensional variation strategy and adaptive parameter strategy is proposed. The main purpose of the proposed ICOA is to prevent the premature local convergence of the COA and to solve some problems with low precision, so as to improve the accuracy of the COA and expand the search ability of the COA. (2). Several test sets, engineering examples of different sizes, a set of low- and high-dimensional mathematical functions, two NP problems, and path planning problems were used to verify the performance of the ICOA. This included CEC 2017, CEC 2020, and CEC 2022, and three test sets, as well as six engineering examples, twenty low-dimensional mathematical functions, ten high-dimensional mathematical functions, and, finally, the use of large-scale NP problems, and a hypersonic missile trajectory planning problem.

The following conclusions were obtained using numerical and graphical comparisons of the ICOA with other intelligent optimization algorithms:

(1) The elite chaotic difference strategy improves good initial solutions for the ICOA, prevents blind searches, and ensures a more uniform distribution of populations in space.
(2) The ICOA ranks first in all the CEC 2020 test sets, and tenth out of twelve test functions in the CEC 2022 test set, based on the first set of comparison algorithms. Based on the second set of comparison algorithms on the CEC 2017 and CEC 2020 test sets, respectively, the combined rank is first (rank = 1.6, 1.517). It shows that the Levy flight strategy and dimensional variation strategy and adaptive strategies greatly improve the convergence and search ability of the COA.
(3) The results of six engineering examples and hypersonic factor missile trajectory planning show that the ICOA is more efficient and stable than other algorithms in solving practical engineering problems.
(4) The outcomes obtained from the evaluation of high-dimensional and low-dimensional mathematical problems, along with complex NP problems, demonstrate that the enhanced strategy significantly enhances the optimization capability of COA. Moreover, it also improves the stability in solving large-scale problems. These results imply that the ICOA outperforms the original algorithm in terms of accuracy and the quality of solutions for large-scale problems.

Moving forward, leveraging the superior abilities of the ICOA in handling diverse complex problems, our future work will focus on optimizing even more complex global optimization problems. To validate the performance of the ICOA further, we plan to tackle real-world engineering problems, thus providing practical evidence of its effectiveness. Furthermore, the proposed ICOA has the potential to address practical problems across diverse fields. For instance, it can handle scheduling tasks in fog computing, complex engineering applications, parameter estimation in photovoltaic modeling [76], multi-threshold image segmentation [77], 3D path planning of UAVs, and many more areas.

## Figures and Tables

**Table 1:** Initial parameter settings of all algorithms.

Algorithm	Parameter Name	Parameter Value
COA	adaptive parameters (α, *k*)	[0,1], [0,1]
	C_1_	0.2
	*C*_3_	3
	δ	25
	μ	3
DE	scaling factor (*F*)	[0,1], [0,1]
	crossover rate (*CR*)	0.9
HHO	starting energy (*E*_0_)	[−1,1]
CDO	*S_γ_*	Rand(1,300,000) km/s
	*S_β_*	Rand(1,270,000) km/s
	*S_α_*	Rand(116,000) km/s
	*r*	Rand(0,1)
SSA	*α*	[0,1]
	warning value (*R*_2_)	[0,1]
	safety value (*ST*)	[0.5,1]
	*Q*	Random numbers obeying a normal distribution
ZOA	*r*	[0,1]
	*I*	[1,2]
	*R*	0.01
	*P_s_* (switching probability)	[0,1]
PSO	cognitive and social coefficients	2,2
	inertial constants	[0.2,0.8]
GWO	control parameter (*C*)	[0,2]
ICOA	adaptive parameters (α, *k*)	[0,1], [0,1]
	C_1_	0.2
	C_3_	3
	δ	25
	μ	3
	scaling factor (*F*)	[0.4,0.8]
	C	[1,2]
	*β*	[1,3]
	*beta*	

**Table 2:** Comparison of results between ICOA and other algorithms (10-dimensional CEC 2020 test set).

Fun	Index	COA	DE	PSO	CDO	FFA	SSA	GWO	HHO	ZOA	ICOA
F1	Mean	3838.955208	1,649,311,269	27,581,932.09	13,429,818,435	22,496,879,698	3719.632254	23,520,636.32	331,765.7796	304,236,883.6	101.4910899
	Std	3007.602591	636,805,261.8	50,249,275.9	2,201,887,812	5,949,781,993	3024.707179	77,107,311.27	174,444.6561	629,022,239	1.08284947
	*p*-value	6.796E−08	6.796E−08	6.796E−08	6.796E−08	6.796E−08	6.796E−08	6.796E−08	6.796E−08	6.796E−08	
	Rank	3	8	6	9	10	2	5	4	7	1
F2	Mean	1860.062515	2959.388415	2168.8548	2533.445794	3722.431377	1739.770468	1540.065244	1906.094295	1583.816301	1448.116386
	Std	371.5677643	228.2440872	443.693734	139.4368457	271.604534	297.2513639	239.2009606	268.9545701	195.8931473	165.3887062
	*p*-value	1.235E−07	6.796E−08	5.166E−06	6.796E−08	6.796E−08	7.577E−06	5.250E−01	2.062E−06	1.929E−02	
	Rank	5	9	7	8	10	4	2	6	3	1
F3	Mean	765.9056692	824.7519216	745.4028849	790.2372231	1162.396417	776.1333762	729.6126697	777.9206277	733.8894709	720.5373713
	Std	24.16979102	19.48329086	11.10439105	6.803535685	70.14236489	25.89615022	9.089326895	17.85779598	10.27687566	3.212966537
	*p*-value	1.657E−07	6.796E−08	1.918E−07	6.796E−08	6.796E−08	6.796E−08	3.369E−01	6.796E−08	1.576E−06	
	Rank	5	9	4	8	10	6	2	7	3	1
F4	Mean	1901.438189	2481.410743	2016.755508	12,724.66072	2,852,758.011	1901.891405	1902.100147	1906.276331	2132.995068	1900.926674
	Std	0.941761873	917.4421483	117.983734	1541.024389	3,356,626.586	0.785072281	0.986971043	2.346971549	622.163359	0.275094376
	*p*-value	5.629E−04	6.796E−08	6.796E−08	6.796E−08	6.796E−08	7.898E−08	3.057E−03	6.796E−08	6.796E−08	
	Rank	2	8	6	9	10	3	4	5	7	1
F5	Mean	8779.842918	785,981.119	4674.502969	16,143.39695	25,412,786.18	4657.763874	69,736.58015	24,035.27291	12,421.51888	1710.774526
	Std	6940.834127	458,224.5984	2780.533249	6400.114281	23,626,290.21	2241.418386	156,126.433	21,366.59862	28,288.90637	5.91583468
	*p*-value	6.796E−08	6.796E−08	6.796E−08	6.796E−08	6.796E−08	6.796E−08	6.796E−08	6.796E−08	6.796E−08	
	Rank	4	9	3	6	10	2	8	7	5	1
F6	Mean	1673.010801	1969.695553	1879.7586	1887.077854	2804.447408	1762.075713	1725.944385	1804.850285	1799.678285	1632.171341
	Std	65.81672278	85.90377711	107.7960669	59.2641346	380.7092261	111.049687	91.85497078	111.9965998	99.21741373	66.09978203
	*p*-value	2.745E−04	6.796E−08	1.235E−07	6.796E−08	6.796E−08	1.576E−06	1.415E−05	1.376E−06	2.218E−07	
	Rank	2	9	7	8	10	4	3	6	5	1
F7	Mean	3302.173721	166,506.2818	2650.623114	203,618.0876	10,244,437.84	2960.247247	8129.109758	11,965.88037	6025.036796	2100.672449
	Std	976.9895466	124,776.3342	715.6411572	414.2890983	12,006,509.81	368.9412357	3949.526618	11,200.05409	3260.516645	0.310442338
	*p*-value	6.796E−08	6.796E−08	6.796E−08	6.796E−08	6.796E−08	6.796E−08	6.796E−08	6.796E−08	6.796E−08	
	Rank	4	8	2	9	10	3	6	7	5	1
F8	Mean	2298.249168	2651.362637	2455.892596	3178.842638	4091.028569	2303.541099	2307.123277	2313.534635	2324.233442	2295.819255
	Std	14.16200851	164.2269187	301.5124043	350.8521508	695.1283201	2.622115124	6.105215183	7.086541414	25.59067975	22.55983287
	*p*-value	1.481E−03	6.796E−08	6.796E−08	6.796E−08	6.796E−08	6.917E−07	2.690E−06	1.047E−06	6.796E−08	
	Rank	2	8	7	9	10	3	4	5	6	1
F9	Mean	2747.113428	2794.323809	2824.711465	2910.295441	2992.467684	2724.67449	2733.363408	2778.980095	2687.94217	2655.546401
	Std	7.483765447	10.41871269	116.3742002	20.17641521	55.38320794	97.38811336	55.28598944	105.0872007	135.0390389	117.1652515
	*p*-value	3.987E−06	5.227E−07	2.041E−05	6.796E−08	6.796E−08	3.499E−06	1.199E−01	6.796E−08	5.227E−07	
	Rank	5	7	8	9	10	3	4	6	2	1
F10	Mean	2931.84509	3057.207813	2926.740314	3574.21649	4342.816925	2913.462694	2940.02941	2927.29717	2962.413309	2902.5189
	Std	21.98679946	40.99069672	62.61178719	82.27196576	599.668131	77.36715193	25.20523086	26.01743413	47.41533969	14.40075427
	*p*-value	8.572E−06	6.767E−08	2.553E−07	6.767E−08	6.767E−08	3.488E−06	3.924E−07	1.910E−07	1.910E−07	
	Rank	5	8	3	9	10	2	6	4	7	1
Mean Rank		3.7	8.3	5.4	8.4	10	3.2	4.4	5.7	5	1
Result		3	8	6	9	10	2	4	7	5	1
+/=/−		0/0/10	0/0/10	0/0/10	0/0/10	0/0/10	0/0/10	0/3/7	0/0/10	0/0/10	-

**Table 3:** Comparison results of the ICOA and other algorithms (CEC 2022 test set).

Fun	Index	COA	DE	PSO	CDO	FFA	SSA	GWO	HHO	ZOA	ICOA
F1	Mean	20,199.18258	76,171.53004	944.214098	25,500.33409	228,117,680.1	2142.343332	10,977.10589	2552.37153	9029.770081	300.5671868
	Std	6960.126145	15,952.34818	576.8655192	890.0341877	487,428,142.8	1011.182616	3750.732407	1506.569514	3380.393564	0.569563772
	*p*-value	6.796E−08	6.796E−08	6.796E−08	6.796E−08	6.796E−08	6.796E−08	6.796E−08	6.796E−08	6.796E−08	
	T-p	2.8799E−14	5.7637E−21	0.32526	7.1078E−53	0.024275	2.0347E−07	3.653E−13	2.0027E−05	3.281E−15	
	Rank	7	9	2	8	10	3	6	4	5	1
F2	Mean	462.9261488	1542.444397	518.1279516	2023.324425	9085.491279	450.4501635	476.8470318	478.7412019	581.5630808	443.4221186
	Std	20.65489048	226.519312	55.78232105	37.54926668	2305.804141	18.55134312	15.15469761	27.92909828	82.68006499	20.22370434
	*p*-value	2.041E−05	6.796E−08	1.657E−07	6.796E−08	6.796E−08	3.372E−02	5.166E−06	9.748E−06	6.796E−08	
	T-p	0.0016544	1.2315E−19	0.093626	1.3009E−55	6.882E−17	0.0015629	3.7369E−07	0.00037112	3.5299E−14	
	Rank	3	8	6	9	10	2	4	5	7	1
F3	Mean	623.0433595	662.2285346	650.0185587	660.5528855	724.9135447	633.2590033	603.6815354	653.1966242	642.7965557	605.537832
	Std	15.66140108	5.874410799	7.345405629	5.482014561	11.28644701	13.06274308	2.418327332	8.936694797	7.003045619	5.621803075
	*p*-value	1.037E−04	6.796E−08	6.796E−08	6.796E−08	6.796E−08	3.416E−07	4.903E−01	6.796E−08	6.796E−08	
	T-p	0.00011642	1.42E−25	1.1469E−22	1.791E−28	1.3329E−32	6.3709E−11	0.1865	1.578E−24	1.03E−19	
	Rank	3	9	6	8	10	4	1	7	5	2
F4	Mean	888.2533067	1007.725436	891.7401872	943.9070725	1106.972433	892.0369531	860.729065	883.2562497	856.8945837	855.7576147
	Std	6.47842746	16.11290788	29.81954968	14.48535392	20.87436457	20.45080042	26.00615569	8.748990257	11.64460645	18.53309918
	*p*-value	2.062E−06	6.796E−08	3.705E−05	6.796E−08	6.796E−08	9.748E−06	6.949E−01	7.577E−06	7.557E−01	
	T-p	2.8582E−07	5.8033E−25	0.3365	1.0869E−18	6.2815E−30	3.5426E−06	0.85431	0.00050248	0.84321	
	Rank	5	9	6	8	10	7	3	4	2	1
F5	Mean	2273.261158	7002.985016	2014.682212	3246.482297	14,149.76766	2361.326958	1068.256361	2657.993617	1726.355255	1195.296702
	Std	684.7187447	1357.80889	237.2145198	236.4168559	2168.568502	226.5678246	166.67826	252.3357777	203.9597704	414.2645949
	*p*-value	1.104E−05	6.796E−08	5.874E−06	6.796E−08	6.796E−08	1.918E−07	6.359E−01	7.898E−08	1.997E−04	
	T-p	1.8187E−09	3.7713E−21	2.7513E−07	2.6749E−24	1.1337E−25	2.2516E−18	0.66436	3.4122E−23	4.9521E−09	
	Rank	5	9	4	8	10	6	1	7	3	2
F6	Mean	6205.807376	751,183,223.3	4,006,662.737	4,366,063,148	7,653,231,573	9249.178376	2,452,926.503	94,197.0447	4,793,747.806	5270.092938
	Std	5340.238554	286,302,362.1	7,392,193.816	2,244,628,819	2,712,022,759	7846.915964	5,796,887.435	45,125.89558	9,243,505.854	1257.414628
	*p*-value	2.616E−01	6.796E−08	6.796E−08	6.796E−08	6.796E−08	4.094E−01	2.341E−03	6.796E−08	8.597E−06	
	T-p	0.45534	8.2009E−12	0.38878	8.7357E−07	9.7743E−15	0.0018917	0.0095729	2.1898E−10	0.046577	
	Rank	2	8	6	9	10	3	5	4	7	1
F7	Mean	2089.592129	2271.91531	2140.279569	2318.263345	2543.416973	2104.450093	2064.500046	2151.059602	2107.437366	2042.839982
	Std	39.05661101	48.16049199	42.77406534	38.74995223	126.6730928	34.40202299	39.53293765	38.3737243	20.48748726	25.78606107
	*p*-value	9.278E−05	6.796E−08	2.960E−07	6.796E−08	6.796E−08	8.597E−06	1.782E−03	2.218E−07	1.376E−06	
	T-p	2.9555E−07	3.3136E−21	6.4519E−12	8.4535E−35	5.2263E−24	3.6903E−08	1.032E−05	4.2897E−15	4.0238E−14	
	Rank	3	8	6	9	10	4	2	7	5	1
F8	Mean	2283.48219	2369.128157	2305.432474	2249.272268	12,015.43173	2296.682406	2254.472027	2253.569213	2265.469154	2226.704865
	Std	70.55279957	62.09699179	88.902124	7.649252555	8917.104856	74.24695472	47.34617516	36.8382138	64.68797786	4.137240249
	*p*-value	2.690E−06	6.796E−08	1.413E−07	6.796E−08	6.796E−08	2.690E−06	4.155E−04	2.960E−07	2.356E−06	
	T-p	0.0090022	8.5727E−12	0.0078289	6.4937E−16	0.00017004	6.9008E−07	0.050568	0.00059575	0.0023227	
	Rank	6	9	8	2	10	7	4	3	5	1
F9	Mean	2480.802166	2736.849302	2510.031867	3475.319934	4017.464068	2480.840527	2506.397867	2491.935141	2570.980291	2480.781291
	Std	0.026847953	46.49242323	29.38117105	57.60767292	707.4877568	0.048572622	21.08740525	10.69071173	38.61430545	2.59307E−05
	*p*-value	6.796E−08	6.796E−08	6.796E−08	6.796E−08	6.796E−08	6.796E−08	6.796E−08	6.796E−08	6.796E−08	
	T-p	4.3633E−05	2.9457E−17	0.31491	1.4288E−40	2.8428E−12	4.3547E−06	7.4004E−05	2.3248E−07	2.6177E−12	
	Rank	2	8	6	9	10	3	5	4	7	1
F10	Mean	3511.959839	4992.221658	4691.457587	5530.167967	8369.520043	3582.355547	3344.564435	3760.334536	3431.529403	2653.69296
	Std	839.682141	1461.408448	1189.21698	1435.12401	429.1177911	696.5281943	666.8767227	774.5187084	789.3937417	436.276607
	*p*-value	8.292E−05	1.047E−06	1.576E−06	1.918E−07	6.796E−08	3.293E−05	1.444E−04	5.874E−06	4.166E−05	
	T-p	0.0013553	3.9727E−08	2.9656E−08	1.9522E−24	1.2016E−39	2.2333E−06	7.4658E−06	1.0622E−11	0.0055316	
	Rank	4	8	7	9	10	5	2	6	3	1
F11	Mean	2926.396138	6941.535566	3741.010578	8486.329351	14,492.73344	2930.934778	3333.749222	2955.771598	4621.662054	2900.081251
	Std	94.34590768	612.3533334	1347.21443	25.62362919	2092.5986	91.98415239	250.3778963	83.67962857	834.5871513	112.3701873
	*p*-value	5.115E−03	6.796E−08	6.796E−08	6.796E−08	6.796E−08	1.014E−03	9.127E−08	3.152E−02	6.796E−08	
	T-p	0.45581	2.3514E−26	0.20228	4.6683E−63	1.588E−24	0.70737	2.0536E−09	0.26717	6.1674E−09	
	Rank	2	8	6	9	10	3	5	4	7	1
F12	Mean	2991.628913	3126.413692	3822.163238	3522.035046	4234.63802	3004.66356	2966.912853	3100.938438	3310.47956	2952.554545
	Std	65.21843762	35.09782036	266.7189054	43.82476357	307.0873969	63.11295926	21.76545959	119.8779699	100.2739881	17.37457276
	*p*-value	1.349E−03	6.796E−08	6.796E−08	6.796E−08	6.796E−08	2.596E−05	4.320E−03	2.960E−07	6.796E−08	
	T-p	0.0006654	5.6551E−21	9.1348E−19	9.778E−40	5.179E−26	0.00084472	0.11794	1.1851E−05	2.2374E−18	
	Rank	3	6	9	8	10	4	2	5	7	1
Mean Rank		3.750	8.250	5.500	8.000	10.000	4.25	3.333	5.000	5.250	1.167
Result		3	9	7	8	10	4	2	5	6	1
+/=/−		0/1/11	0/0/12	0/0/12	0/0/12	0/0/12	0/0/12	2/1/9	0/0/12	0/1/11	−

**Table 4:** Initial parameters of the optimization algorithm.

Algorithm	Parameter Name	Reference Point
SaDE	Scaling factor (F)	0.5
	Crossover rate (CR)	0.9
	Probability (p)	0.5
GQPSO	U, ψ, t	[0,1]
	Contractile expansion factor (β)	0
	Gaussian parameter (σ)	0.16
GOA	Attractive force (f)	0.5
	Attractive Length Scale (l)	1.5
	g (gravitational constant)	9.8 m/s
WOA	B (spiral shape parameters)	[0,1]
	I	Rand[−1,1]
	P (probability of a predation mechanism)	Rand[0,1]
	a (convergence factor)	Random numbers obeying a normal distribution
AOA	Constant (C1,C2,C3,C4)	2, 6,1,2
IGWO	Control parameter (C)	[0,2]
ISSA	e	Constant
	Step Control Parameters (β)	N(0,1)

**Table 5:** Comparison results of ICOA and other algorithms (CEC2020 test set).

**Fun**	**Index**	**COA**	**SaDE**	**GQPSO**	**AOA**	**GOA**	**WOA**	**IGWO**	**ISSA**	**TGA**	**ICOA**
F1	Mean	3080.590138	205.6304801	9,625,110,388	213,202,816.4	4,379,490,880	302.2809194	18,471.77694	2600.286025	945,491,194.5	101.4098254
	Std	2180.04528	325.7148828	1,856,910,397	469,523,140.8	2,300,629,583	276.0878092	8809.910178	2930.092116	417,299,520.2	1.055445014
	*p*-value	6.796E−08	5.792E−01	6.796E−08	6.796E−08	6.796E−08	6.015E−07	6.796E−08	4.517E−07	6.796E−08	
	Rank	5	2	10	7	9	3	6	4	8	1
F2	Mean	1913.389008	1893.559565	3151.297789	1674.843367	2185.228444	1881.893178	1376.82082	1895.820672	2174.84804	1458.234238
	Std	350.947033	139.3642723	208.173789	299.890429	177.503404	284.7332401	309.3453585	454.2873522	286.2958063	151.7652699
	*p*-value	1.444E−04	6.674E−06	6.796E−08	1.988E−01	1.235E−07	2.341E−03	6.787E−02	3.605E−02	5.227E−07	
	Rank	7	5	10	3	9	4	1	6	8	2
F3	Mean	774.7307259	729.6251739	821.2219869	749.065088	767.3536999	741.4243149	720.5099737	748.2369501	806.9572282	721.0349346
	Std	26.26519819	4.856728826	11.94414125	14.12349489	15.62120522	12.0471497	9.222011657	20.64321962	16.53419446	3.460709391
	*p*-value	5.166E−06	1.075E−01	6.796E−08	1.415E−05	2.563E−07	5.091E−04	2.073E−02	1.199E−01	6.796E−08	
	Rank	8	3	10	6	7	4	1	5	9	2
F4	Mean	1901.580562	1901.957154	76,717.82357	1903.63955	10,187.43812	1904.119445	1902.275775	1901.332688	1932.668404	1900.810655
	Std	0.802246966	0.426050124	38,034.26218	2.320743122	9688.677624	2.11978368	0.406946809	0.602736435	25.78167582	0.259623522
	*p*-value	9.278E−05	6.796E−08	6.796E−08	4.539E−07	6.796E−08	3.939E−07	6.796E−08	4.601E−04	6.796E−08	
	Rank	3	4	10	6	9	7	5	2	8	1
F5	Mean	8256.462973	7408.827254	472,957.6182	4502.521987	434,060.0981	2106.790299	2945.722538	4610.72922	43,167.68559	1710.941224
	Std	7614.405859	24,896.86396	138,356.9039	2161.335567	165,625.822	211.3949486	1091.811168	2432.125686	21,813.42188	11.33206389
	*p*-value	6.796E−08	5.166E−06	6.796E−08	6.796E−08	6.796E−08	2.563E−07	6.796E−08	7.898E−08	6.796E−08	
	Rank	7	6	10	4	9	2	3	5	8	1
F6	Mean	1655.891051	1615.890949	2276.26678	1759.606751	1980.67376	1737.957277	1603.577489	1737.65658	1735.497451	1627.109196
	Std	72.55343198	37.67620158	141.5933706	62.76771617	125.8651205	84.48050077	3.534907476	96.23718895	82.33130371	53.47052931
	*p*-value	1.017E−01	2.616E−01	6.796E−08	2.222E−04	2.960E−07	3.648E−01	1.988E−01	4.601E−04	1.794E−04	
	Rank	4	2	10	8	9	7	1	6	5	3
F7	Mean	3059.110232	2208.463589	566,560.7392	6167.232251	227,056.1337	2213.372227	2499.991755	2450.206146	9566.176153	2101.420693
	Std	512.136508	428.6440918	385,508.4616	2838.195448	373,754.5143	104.6616754	174.3121823	243.6110536	6823.843586	3.747408303
	*p*-value	6.796E−08	7.579E−04	6.796E−08	6.796E−08	6.796E−08	2.563E−07	9.173E−08	1.657E−08	6.796E−08	
	Rank	6	2	10	7	9	3	5	4	8	1
F8	Mean	2301.756398	2301.703051	2898.593507	2276.194191	2571.806725	2304.837157	2299.517007	2302.441919	2348.360521	2295.869634
	Std	0.792001319	1.049953855	90.46905295	43.57611423	114.4493889	13.94386472	23.46321882	2.421320571	50.85137029	22.57289382
	*p*-value	3.048E−04	1.898E−01	6.796E−08	2.853E−01	6.798E−06	1.201E−06	1.201E−06	1.443E−04	1.610E−04	
	Rank	5	4	10	1	9	7	3	6	8	2
F9	Mean	2719.099613	2736.944804	2865.818387	2689.714583	2791.392109	2722.224321	2727.021655	2725.784094	2636.092881	2612.083805
	Std	75.20737403	57.829986	77.81898066	135.6287001	92.66929128	96.4206922	53.89724331	85.39273019	48.87360673	118.8353758
	*p*-value	3.293E−05	2.139E−03	5.277E−07	2.471E−04	5.428E−01	2.690E−04	2.184E−01	9.173E−08	9.173E−08	
	Rank	4	8	10	3	9	5	7	6	2	1
F10	Mean	2920.399654	2923.110414	3269.564556	2934.449531	3076.6118	2916.957328	2898.095842	2925.269496	2989.697136	2905.437014
	Std	64.21061213	23.36802629	52.21517864	26.84240033	106.3725155	64.29011463	0.331899138	23.06363759	24.90496444	17.26832487
	*p*-value	1.791E−04	3.636E−03	6.776E−08	2.219E−04	6.776E−08	3.636E−03	9.461E−01	3.696E−05	6.776E−08	
	Rank	4	5	10	7	9	3	1	6	8	2
Mean Rank		5.3	4.1	10	5.5	9	5.1	3.1	5.1	7.2	1.6
Result		6	3	10	7	9	5	2	5	8	1
+/=/−		1/0/9	1/3/6	0/0/10	1/1/8	0/0/10	0/0/10	3/1/6	0/1/9	0/0/10	-

**Table 6:** Comparison of results between ICOA and other algorithms (10-dimensional CEC 2017 test set).

**Fun**	**Index**	**COA**	**SaDE**	**GQPSO**	**AOA**	**GOA**	**WOA**	**IGWO**	**ISSA**	**TGA**	**ICOA**
F1	Best	148.6730345	4747.622261	1,466,633,185	3,822,463.598	334,306,252.5	100.2893825	2041.073121	125.572396	370,114,699.2	159.9555967
	Worst	9549.967839	46,364.35967	2,895,384,603	147,860,147	2,034,750,876	16,182.00445	34,064.5399	18,045.25743	1,313,880,441	1797.617184
	Mean	4116.594259	19,510.53251	2,033,179,558	54,641,045.48	967,098,811.1	2548.662962	7622.634467	3817.315048	773,490,841.5	510.4325359
	Std	3451.740321	12,395.32945	374,346,351.6	49,935,698.39	508,659,189.6	4117.276675	7762.961803	5461.116842	246,047,598.6	348.6744138
	*p*-value	0.0133205	6.79562E−08	6.79562E−08	6.79562E−08	6.79562E−08	0.261616	6.79562E−08	0.0090454	6.79562E−08	
	Rank	4	6	10	7	9	2	5	3	8	1
F3	Best	300.0115365	300.0262972	11,337.31372	302.8949062	10,684.80791	300	300.0191866	300	3212.316168	300
	Worst	372.363719	920.3804805	20,813.75722	2313.191007	18,890.01384	300	300.2383643	300.0000001	11,162.33462	300
	Mean	304.9282754	346.3590271	15,508.93347	650.9262713	14,528.15198	300	300.0642843	300	6492.62042	300
	Std	15.98248703	139.7252079	2554.692367	568.472826	2311.010429	1.74298E−12	0.052175512	2.72674E−08	2171.224631	4.25057E−11
	*p*-value	6.75738E−08	6.75738E−08	6.75738E−08	6.75738E−08	6.75738E−08	0.100444	6.75738E−08	1.91209E−05	6.75738E−08	
	Rank	5	6	10	7	9	1	4	3	8	2
F4	Best	400.0319889	406.3324266	494.8966315	403.3790854	435.0131351	400.0007614	400.7881249	400.0189761	416.3654147	400
	Worst	409.5988797	409.6791913	613.0620111	444.1383144	617.0037101	407.5123122	406.0013963	409.3511312	511.6735362	403.9865791
	Mean	404.6699325	407.3377881	541.9514887	419.8505524	510.276305	401.6576344	402.1708677	403.8830054	453.9491199	400.7973158
	Std	3.01023269	0.698428281	27.51270522	14.39207761	41.22755759	2.681130732	1.037346312	2.661658963	23.74991351	1.636057547
	*p*-value	1.06166E−07	7.876E−08	6.77647E−08	9.14744E−08	6.77647E−08	9.10523E−07	6.90001E−07	2.95221E−07	6.77647E−08	
	Rank	5	6	10	7	9	2	3	4	8	1
F5	Best	507.9603546	517.1084588	547.0449047	507.960565	518.4506157	502.9848772	500.9953028	513.9294167	544.4969693	501.9899181
	Worst	526.8638492	531.20374	565.9932599	525.4408086	542.4827219	519.899161	516.2317563	553.7275871	564.9711659	517.9092429
	Mean	517.9901144	524.5896705	554.8746013	516.6819111	530.6707215	513.3821867	505.2230268	526.8638368	555.976207	509.8363908
	Std	4.930060129	3.834882042	6.742883423	4.743381383	7.532670353	4.76044302	3.289994642	8.655773513	6.265983605	3.953514287
	*p*-value	2.92486E−05	6.79562E−08	6.79562E−08	5.89592E−05	6.79562E−08	2.59146E−05	4.6804E−05	1.19538E−06	6.79562E−08	
	Rank	5	6	9	4	8	3	1	7	10	2
F6	Best	600.0646378	600	629.9998944	601.3325972	610.9706087	600.7012471	600.045807	600	611.6621673	600.00015
	Worst	648.918318	600	660.8601099	623.7118128	636.6164527	621.3363286	600.0843116	625.6466821	626.2959508	600.0045008
	Mean	608.8001365	600	649.0458874	607.860604	622.6489137	607.2577357	600.0621377	605.1406886	618.7073681	600.001341
	Std	14.27519752	5.21631E−14	6.466445852	6.165384799	6.097467331	6.369953587	0.01085008	7.091281916	4.098979244	0.001080142
	*p*-value	7.89803E−08	1.94473E−08	6.79562E−08	6.79562E−08	6.79562E−08	6.79562E−08	6.79562E−08	0.00134858	6.79562E−08	
	Rank	7	1	10	6	9	5	3	4	8	2
F7	Best	712.013249	711.412164	818.3422902	727.1132342	742.2848267	726.6088087	712.577756	716.2732618	752.1640413	714.3827533
	Worst	855.8382714	725.1023536	857.3651285	806.1758234	832.9248346	797.1786426	737.0763445	822.6989394	825.4520316	726.3788175
	Mean	762.0943164	721.2208093	835.2569854	755.089176	776.097165	742.8577434	720.7600727	744.8829921	797.6770415	718.9003091
	Std	46.23078803	3.480685354	11.13038834	22.26800215	23.82012741	16.12983542	6.964089443	31.11810908	20.62497699	2.927239924
	*p*-value	0.00432018	0.00234127	6.79562E−08	6.79562E−08	6.79562E−08	6.79562E−08	0.989209	0.000179364	6.79562E−08	
	Rank	7	3	10	6	8	4	2	5	9	1
F8	Best	805.9698249	807.2457367	874.0694682	820.0699399	834.9417722	810.9445396	801.9954459	806.9647084	825.6936112	802.9875186
	Worst	876.6114734	819.664581	902.3440368	851.2843135	860.1755839	836.8134044	816.4533424	871.6366218	860.5124113	813.3907572
	Mean	831.880596	814.0204409	886.95372	837.4797929	848.1388623	826.664851	808.7544151	830.7943993	843.2812621	807.9906668
	Std	21.8691896	3.212999361	8.289923104	9.016481538	8.657846055	7.258476257	5.014726193	18.5960468	7.934483059	3.145065154
	*p*-value	1.65708E−07	6.91658E−07	6.79562E−08	6.79562E−08	6.79562E−08	2.21199E−07	0.0133205	6.79562E−08	6.79562E−08	
	Rank	6	3	10	7	9	4	2	5	8	1
F9	Best	900.0898247	900	1902.294749	902.040387	934.0248542	903.7265596	900.0020026	900	996.5772439	900
	Worst	1194.18378	900.000006	2684.76882	1032.761239	1601.124472	1119.018646	900.0217193	1768.582753	1465.8854	900
	Mean	933.9467429	900.0000005	2299.918782	927.4919847	1296.20627	955.1920242	900.0063842	1009.225415	1173.247669	900
	Std	76.80007338	1.36528E−06	213.5026121	35.89157465	205.0921803	54.73903135	0.004752395	252.8979347	103.4789807	4.16317E−12
	*p*-value	6.6344E−08	0.0249501	6.6344E−08	6.6344E−08	6.6344E−08	6.6344E−08	6.6344E−08	6.6344E−08	6.6344E−08	
	Rank	5	2	10	4	9	6	3	7	8	1
F10	Best	1340.205272	1702.723011	2759.206735	1625.225604	1781.590766	1118.625698	1003.938585	1559.422686	2072.158365	1024.77159
	Worst	2600.153734	2280.962891	3373.207944	2392.123462	2914.720417	2244.645683	2200.964033	2554.446062	2570.300006	1872.259067
	Mean	2003.512135	2042.819847	3053.524005	1908.552584	2241.274063	1698.940649	1314.665194	2062.679711	2310.331481	1466.530483
	Std	346.214506	135.3497372	180.4873836	238.866608	299.1371429	309.1977504	358.1750795	273.7979532	140.259575	228.2632009
	*p*-value	0.0467916	1.06457E−07	6.79562E−08	9.74798E−06	9.17277E−08	0.00396624	0.00711349	0.00655719	6.79562E−08	
	Rank	5	6	10	4	8	3	1	7	9	2
F11	Best	1113.679736	1109.982564	1235.455022	1119.210569	1159.267803	1103.994993	1102.047622	1113.929879	1137.933666	1100.99496
	Worst	1209.016831	1128.279027	2054.946919	1207.719924	1790.690271	1169.897166	1112.049674	1188.598762	1263.068502	1105.121276
	Mean	1149.640666	1118.410649	1590.222885	1143.849619	1294.255116	1129.311185	1107.441434	1151.423711	1206.470897	1102.621416
	Std	28.54712401	5.515854063	225.5279052	20.77982629	167.9049579	17.51230869	2.516341575	21.18974098	33.63882861	1.447853224
	*p*-value	6.79562E−08	6.79562E−08	6.79562E−08	6.79562E−08	6.79562E−08	6.79562E−08	1.65708E−07	6.79562E−08	6.79562E−08	
	Rank	6	3	10	5	9	4	2	7	8	1
F12	Best	49,086.80101	51,710.13324	84,401,058.7	312,238.847	3,216,470.292	2518.11056	5660.218148	30,000.81079	11,007,844.02	2138.110046
	Worst	4,925,172.527	662,879.8469	738,709,578.8	9,956,555.414	188,423,358.7	21,571.93069	87,688.82884	2,630,148.307	49,842,458.1	4318.510312
	Mean	1,242,192.291	248,406.3993	233,880,579.8	3,353,132.964	78,363,722.71	8393.654438	34,571.38395	810,606.4495	30,300,014.75	2897.07194
	Std	1,515,129.883	188,509.6202	160,770,530.5	3,104,917.499	61,884,597.91	4356.645386	24,143.10043	767,394.4802	11,563,636.79	604.365916
	*p*-value	6.79562E−08	6.79562E−08	6.79562E−08	6.79562E−08	6.79562E−08	0.000247061	6.79562E−08	6.79562E−08	6.79562E−08	
	Rank	6	4	10	7	9	2	3	5	8	1
F13	Best	1464.502063	1303.614389	234,592.877	7798.968137	3181.131074	1313.166907	1450.97058	1343.791528	2682.967698	1302.095923
	Worst	30,355.92992	2018.738554	39,033,982	16,571.90555	299,294.4821	1799.906751	3770.676689	26,299.72566	156,322.6181	1313.32242
	Mean	5051.762467	1347.566437	14,765,418.42	12,016.00972	27,623.06315	1444.067248	2032.518714	10,697.12595	57,245.24785	1307.709977
	Std	6461.487512	158.0496691	10,008,094.31	2371.315403	64,439.3798	152.1812383	564.1194136	7824.806354	51,753.89921	3.113739726
	*p*-value	6.79562E−08	0.0010141	6.79562E−08	6.79562E−08	6.79562E−08	1.43085E−07	6.79562E−08	6.79562E−08	6.79562E−08	
	Rank	5	2	10	7	8	3	4	6	9	1
F14	Best	1473.538584	1401.544496	3160.52319	1458.780152	1540.598141	1414.224591	1434.948321	1430.542775	1445.76419	1400.000465
	Worst	1769.199744	1423.691449	367,734.9812	1698.487089	17,355.75621	1462.788457	1478.557902	1615.160224	2083.948978	1421.091345
	Mean	1561.290972	1411.532603	32,677.61565	1508.067102	5232.871595	1432.486756	1447.580479	1486.330768	1612.813498	1403.319026
	Std	79.54448354	7.532606185	79,637.66882	55.24829574	3527.990105	12.99994955	11.50540784	43.96708702	170.8966513	4.84782392
	*p*-value	6.79562E−08	0.000103734	6.79562E−08	6.79562E−08	6.79562E−08	6.79562E−08	6.79562E−08	6.79562E−08	6.79562E−08	
	Rank	7	2	10	6	9	3	4	5	8	1
F15	Best	1642.426261	1500.977628	7215.229382	1576.417867	3075.410582	1502.267152	1509.227541	1504.349621	1615.454848	1500.135994
	Worst	4595.486555	1503.084721	36,872.23217	2425.177685	20,522.59548	1749.767496	1565.53437	1782.956834	5737.077925	1502.575169
	Mean	2288.07323	1501.588285	20,457.77691	1707.253547	12,045.02805	1548.691697	1527.478627	1606.68848	2814.293154	1500.969889
	Std	873.8758204	0.544793001	8393.159846	200.6312783	4949.442088	58.20643665	16.66020498	79.78714845	1257.992628	0.731431977
	*p*-value	6.79562E−08	0.0010141	6.79562E−08	6.79562E−08	6.79562E−08	6.79562E−08	6.79562E−08	6.79562E−08	6.79562E−08	
	Rank	7	2	10	6	9	4	3	5	8	1
F16	Best	1602.072533	1600.814769	1931.679748	1629.698131	1673.683166	1600.72474	1601.460034	1612.363747	1618.760687	1600.033067
	Worst	1879.539676	1638.895802	2381.833927	1983.177623	2130.934168	1975.775027	1613.080576	2151.827937	1841.650088	1838.422985
	Mean	1676.449187	1603.773439	2172.171474	1808.567854	1906.638735	1776.971062	1603.757232	1834.744052	1696.736922	1614.012491
	Std	89.50347045	8.312587623	107.7315397	132.5891305	129.6763427	156.5673765	2.777722188	144.9325342	68.82161905	52.9725289
	*p*-value	8.29242E−05	0.00305663	6.79562E−08	1.43085E−07	6.79562E−08	1.37606E−06	0.000920913	1.59972E−05	4.53897E−07	
	Rank	4	2	10	7	9	6	1	8	5	3
F17	Best	1718.157587	1703.039653	1785.493373	1728.851833	1736.833778	1723.965614	1718.229433	1714.290888	1735.87187	1702.443984
	Worst	1787.309424	1728.132939	1942.167889	1818.992137	1820.217455	1801.046287	1738.545685	1769.712397	1801.496542	1723.230288
	Mean	1736.117118	1719.051557	1849.253632	1776.701669	1770.35599	1755.039159	1732.30593	1738.298362	1763.901722	1713.32226
	Std	18.64870398	8.202620992	38.60703888	29.95869252	21.06926558	18.69967416	5.878406308	13.87109743	15.97546064	8.378076252
	*p*-value	9.74798E−06	0.00162526	6.79562E−08	6.79562E−08	6.79562E−08	4.53897E−07	6.67365E−06	5.87357E−06	6.79562E−08	
	Rank	4	2	10	9	8	6	3	5	7	1
F18	Best	2376.916174	1801.862066	38,0311.4025	3058.409228	4944.324569	1824.201708	2395.57849	1867.972942	9172.512763	1801.809688
	Worst	29,165.84454	14,073.78964	236,981,725.7	20,275.6516	90,437,745.23	2143.422925	15,224.32684	6515.278517	901,377.02	1823.819475
	Mean	12,335.73263	2560.003795	73,228,735.24	7266.778592	8442,913.2	1880.873433	5197.877398	3520.611854	156,798.0173	1818.361647
	Std	7381.60704	2774.913835	66,232,838.86	4182.198992	20,607,718.21	85.95089854	2933.666377	1479.373335	210,322.5584	5.290637159
	*p*-value	6.79562E−08	0.00037499	6.79562E−08	6.79562E−08	6.79562E−08	6.79562E−08	6.79562E−08	6.79562E−08	6.79562E−08	
	Rank	7	3	10	6	9	2	5	4	8	1
F19	Best	1941.427858	1900.014291	15,096.12186	1910.109168	2528.87291	1903.105408	1910.038111	1905.362636	1957.665028	1900.191068
	Worst	2237.896631	2062.806519	3,586,326.534	2288.843408	139,537.5399	1953.894671	1957.407252	3063.363137	23,836.46081	1903.769604
	Mean	2066.925017	1908.791089	1,034,285.832	1988.082547	26,112.51057	1916.026028	1920.837231	2038.711476	5706.609991	1901.56281
	Std	75.69770571	36.25686385	914,707.6459	87.63851349	33,168.082	14.63271918	10.79753957	247.5790024	5104.589963	0.814218167
	*p*-value	6.79562E−08	3.93881E−07	6.79562E−08	6.79562E−08	6.79562E−08	6.79562E−08	6.79562E−08	6.79562E−08	6.79562E−08	
	Rank	7	2	10	5	9	3	4	6	8	1
F20	Best	2001.014591	2000	2184.35236	2035.924246	2055.581504	2023.288704	2000.630387	2020.994959	2034.338932	2000.000003
	Worst	2140.616195	2020.000108	2383.675336	2154.476938	2224.511173	2211.542977	2029.871349	2286.179344	2086.015194	2002.614276
	Mean	2031.725628	2001.255965	2291.729588	2078.219251	2133.897235	2083.546958	2023.103498	2091.459777	2054.967087	2000.639073
	Std	38.03856914	4.426153907	51.89895192	30.98063902	61.98797225	58.8322243	8.839276077	94.61067455	13.9168936	0.629731914
	*p*-value	5.16578E−06	2.15196E−05	6.79562E−08	6.79562E−08	6.79562E−08	6.79562E−08	4.53897E−07	7.89803E−08	6.79562E−08	
	Rank	4	2	10	6	9	7	3	8	5	1
F21	Best	2200.000288	2201.442816	2257.208729	2200.725549	2244.660619	2200	2200.00712	2200	2208.885227	2200
	Worst	2328.345583	2329.616604	2415.128603	2342.048598	2365.898552	2350.931441	2315.873089	2351.228736	2254.60066	2315.521808
	Mean	2299.264412	2296.906268	2347.603893	2287.672342	2336.154801	2293.240593	2274.58558	2321.942684	2224.255702	2272.128253
	Std	42.76235732	50.38874297	54.97731017	62.5639353	35.4892308	56.59043356	50.20058707	31.65451179	12.28427422	54.36216179
	*p*-value	7.4064E−05	0.00256062	6.67365E−06	6.67365E−06	0.000920913	0.00363724	0.839232	0.0034593	0.597863	
	Rank	7	6	10	4	9	5	3	8	1	2
F22	Best	2211.396651	2300	2736.149617	2211.582351	2287.336263	2248.676943	2300.305958	2300.634359	2289.714737	2300.001265
	Worst	2304.215273	2302.95023	3116.628538	2399.351642	2733.751613	2315.332928	2307.323861	2305.566178	2429.336917	2301.877899
	Mean	2297.120251	2301.390748	2913.38705	2276.53314	2539.189047	2301.809952	2304.491472	2302.249202	2339.265125	2300.85498
	Std	20.19116633	0.988251463	108.1375634	52.14553769	106.2415888	15.29909838	2.063791027	1.289372878	38.78497137	0.481061092
	*p*-value	0.0411236	0.635945	6.79562E−08	0.285305	6.79562E−08	1.59972E−05	1.59972E−05	0.00037499	1.59972E−05	
	Rank	2	4	10	1	9	5	7	6	8	3
F23	Best	2606.683756	2609.44154	2717.956329	2630.623033	2658.384597	2609.173339	2600.026068	2613.230456	2637.866195	2605.504363
	Worst	2624.83626	2628.868563	2796.735723	2698.613684	2750.898355	2648.690924	2620.330743	2661.000135	2673.177312	2614.139535
	Mean	2615.138414	2618.13077	2755.687932	2654.815315	2692.002546	2629.443534	2607.779384	2628.012646	2658.09126	2610.531075
	Std	5.810837185	4.612523571	20.61684947	17.78184758	22.35059711	13.0051251	5.450602421	14.33214884	9.259948992	2.092261325
	*p*-value	2.59598E−05	1.04727E−06	6.79562E−08	7.89803E−08	6.79562E−08	9.17277E−08	0.00835483	2.21776E−07	6.79562E−08	
	Rank	3	4	10	7	9	6	1	5	8	2
F24	Best	2736.232907	2500	2717.001814	2503.925307	2565.94435	2500	2500.001603	2601.238787	2584.046056	2500
	Worst	2760.967177	2757.564707	3036.964506	2860.78774	2893.184787	2778.707725	2750.983691	2793.687614	2783.809026	2742.948767
	Mean	2745.531147	2739.786169	2863.480742	2722.002857	2778.892524	2717.077773	2722.294664	2743.265345	2634.974742	2643.449909
	Std	7.722312473	56.60918036	84.2173108	125.9998303	110.5584087	94.02668963	53.31977353	51.04891404	43.08010434	120.1871294
	*p*-value	5.87357E−06	1.59972E−05	1.37606E−06	0.000222203	2.56295E−07	0.000160867	0.00178238	2.92486E−05	0.023551	
	Rank	8	6	10	4	9	3	5	7	1	2
F25	Best	2897.779919	2897.742869	3159.158628	2897.931796	3002.703591	2897.742869	2897.744612	2897.742869	2962.220337	2897.742869
	Worst	2950.616304	2946.173865	3372.805505	3013.036853	3448.390397	2960.138602	2943.552922	2949.452968	3041.853281	2943.442847
	Mean	2929.324667	2926.226372	3262.716944	2936.149484	3147.34603	2923.296537	2900.304735	2919.776322	2991.83964	2906.968546
	Std	22.89858816	23.13923149	50.66094224	29.30352266	133.04327	25.29040909	10.18656304	23.73700915	21.16118212	18.69368178
	*p*-value	9.03062E−07	0.000505985	6.70985E−08	5.11864E−06	6.70985E−08	0.000303011	0.0557225	3.9337E−06	6.70985E−08	
	Rank	6	5	10	7	9	4	1	3	8	2
F26	Best	2600.191002	2800	3892.409608	2607.296395	3398.746817	2600	2600.002129	2800	2981.151544	2800
	Worst	4091.033317	3946.639464	4552.205809	4007.049841	4480.062983	3320.859028	3776.632371	3094.856296	3213.945423	2900.000003
	Mean	3073.165897	3011.737335	4221.721711	2963.626914	3864.678286	2960.585316	2928.835482	2928.627505	3109.610743	2880.000001
	Std	340.8278619	302.7313026	173.3020535	324.6732024	364.7951636	218.7965612	210.4948225	93.76089304	63.62768929	41.03913375
	*p*-value	6.79562E−08	0.499991	6.79562E−08	0.000835717	6.79562E−08	0.033536	6.79562E−08	0.006031	6.79562E−08	
	Rank	7	6	10	5	9	4	3	2	8	1
F27	Best	3088.978017	3088.978013	3170.135975	3093.006368	3112.245217	3089.51799	3089.010826	3089.308077	3099.926184	3088.978013
	Worst	3134.804696	3115.69597	3445.418406	3200.00201	3264.839069	3133.273142	3091.111508	3190.342281	3106.561916	3093.434321
	Mean	3098.311749	3092.48629	3305.70977	3125.080092	3208.795264	3102.644445	3089.458702	3111.125726	3103.836089	3089.761299
	Std	9.839404722	5.858560544	62.48650579	39.68742195	38.75346512	8.881716786	0.419478153	31.92833212	1.802337106	1.212620095
	*p*-value	2.67821E−06	0.000303408	6.75738E−08	1.19538E−06	1.42319E−07	1.36981E−06	0.524909	1.36981E−06	1.19538E−06	
	Rank	4	3	10	8	9	5	1	7	6	2
F28	Best	3100.003662	3100	3679.425868	3278.743157	3216.853772	3100	3100.031164	3100	3212.117701	3100
	Worst	3731.812926	3411.821834	3900.368061	3750.410599	3821.477124	3411.821808	3411.821808	3736.179973	3415.915702	3411.821808
	Mean	3312.386349	3262.785437	3815.819029	3316.778468	3612.108355	3238.458897	3189.321239	3311.586625	3292.308424	3208.348486
	Std	164.0830227	115.4701764	64.51116625	102.8066089	183.5683596	157.1689589	140.1209232	173.9509623	51.35725851	142.9493947
	*p*-value	0.00193426	0.012067	6.6063E−08	0.140047	1.76322E−06	0.118099	0.473065	0.805552	0.635627	
	Rank	7	4	10	8	9	3	1	6	5	2
F29	Best	3146.975965	3148.89887	3352.317094	3172.397231	3183.39145	3162.883648	3131.509681	3154.99773	3177.656624	3128.083943
	Worst	3336.531776	3224.477388	3654.747345	3310.902854	3428.037144	3326.159227	3171.98856	3374.347575	3269.568677	3147.167077
	Mean	3220.610654	3186.266874	3491.34956	3232.178731	3292.500753	3233.801119	3150.555883	3234.01356	3230.037076	3133.342165
	Std	66.27362354	19.0421135	85.93200434	34.60413195	61.25164891	44.53851896	11.67492156	57.29806787	30.30232837	4.760727689
	*p*-value	9.17277E−08	6.79562E−08	6.79562E−08	6.79562E−08	6.79562E−08	6.79562E−08	0.000160981	7.89803E−08	6.79562E−08	
	Rank	4	3	10	6	9	7	2	8	5	1
F30	Best	3976.846139	3908.469505	5,545,090.331	3275.112266	24,485.18651	3420.782263	3721.251739	4354.413685	128,224.8679	3443.647265
	Worst	1,384,854.465	1,251,762.743	32,821,146.7	732,342.488	40707507	1,251,762.743	8250,23.8339	1,251,762.743	2,055,685.572	1,251,762.743
	Mean	469,355.0617	170,001.384	18,121,356.67	161,197.8493	12,076,824.32	106,806.0944	87,603.44456	236,546.66	842,956.9005	106,779.5696
	Std	608,883.7639	385,324.6157	6,301,155.986	186,849.8132	13,396,013.56	325,441.6895	252,107.0941	406,876.0247	362,331.6332	325,450.5459
	*p*-value	2.56295E−07	1.04727E−06	6.79562E−08	0.000129405	9.17277E−08	0.011429	1.37606E−06	5.22689E−07	1.05847E−07	
	Rank	7	5	10	4	9	3	1	6	8	2
Mean Rank		5.551	3.758	10.000	5.379	8.827	3.965	2.863	5.413	7.096	1.517
Result		7	3	10	5	9	4	2	6	8	1
+/=/−		0/0/29	0/2/27	0/0/29	1/1/27	0/0/29	1/2/26	3/2/24	0/1/28	1/1/27	-

**Table 7:** Results of reducer design problems.

Algorithms	Variables							Optimum Value
	*ic*_1_	*ic*_2_	*ic*_3_	*ic*_4_	*ic*_5_	*ic*_6_	*ic*_7_	
COA	3.500144301	0.700013637	17	7.3	7.8	3.350234579	5.286747711	2996.512548
ICOA	3.5	0.7	17	7.300000001	7.8	3.350214666	5.28668323	2996.348165
GWO	3.500578809	0.7	17	7.372778983	7.8	3.351509936	5.288268428	2998.556168
HHO	3.500006329	0.7	17	7.435689307	7.8	3.350470379	5.294064515	3002.312787
DO	3.500021374	0.7	17	7.302661238	7.800049636	3.350251844	5.286696101	2996.39877
WFO	2.605192109	0.700360394	17.02620326	7.594130815	8.116458587	2.902330636	5.000658902	100,002,377.9
GOA	3.500019156	0.7	17	7.3	7.8	3.350313811	5.287116607	2996.656585
SSA	2.6	0.7	17	7.3	7.8	2.9	5	100,002,530.8
FFA	3.563925817	0.7	17	7.3	7.8	3.505693735	5.286829428	3062.924719
AOA	3.6	0.7	17	8.3	7.8	3.478050297	5.309190914	3093.161996

**Table 8:** Statistical results of reducer Problemsproblems.

Algorithms	Best	Worst	Mean	Std
COA	2996.512548	5440.50617	3119.577601	546.2929035
ICOA	2996.348165	5628.074172	3139.487761	588.0200818
GWO	2998.556168	3014.760517	3004.128398	3.68652866
HHO	3002.312787	5572.407625	3655.28318	1111.043272
DO	2996.39877	3006.021464	2999.219925	2.793965817
WFO	100,002,377.9	100,002,470.5	100,002,421.3	27.49125769
GOA	2996.656585	3000.477606	2997.890371	1.187587807
SSA	100,002,530.8	100,003,226.2	100,002,939.3	167.8427193
FFA	3062.924719	11,118,255.94	5,449,561.858	4,226,598.291
AOA	3093.161996	3228.722518	3161.790426	52.68475681

**Table 9:** Hydrostatic thrust bearing design problem results.

Algorithms	Variables				Optimum Value
	*r*	*r*_0_	*μ*	*q*	
COA	6.665774353	6.680884473	7.26308 × 10^−6^	1	4665.174504
ICOA	8.15369663	8.153788022	9.96607× 10^−6^	1.023513439	958.0737549
GWO	6.679918959	6.683393826	9.60624× 10^−6^	2.067179509	3418.088762
AO	7.987719182	6.54381018	7.83822× 10^−6^	15.87088783	62,719.0719
SO	9.090368429	9.091099374	9.03321× 10^−6^	1	1814.372174
DO	7.079945039	7.08152603	9.18667× 10^−6^	1.007373651	2013.67956
WFO	6.03724118	6.110820753	9.35983× 10^−6^	6.036913245	13,500.33491
GOA	7.9321792	7.94973761	6.09553× 10^−6^	1	6351.059315
FFA	13.57299586	13.57404632	9.80632× 10^−6^	6.477130763	4267.49196
GRO	7.794522083	7.872572662	5.58822× 10^−6^	1.936312513	2414.211291
AOA	8.98628022	8.987954146	1.01511× 10^−6^	16	10,111.4088

**Table 10:** Statistical results of hydrostatic thrust bearing design problem results.

Algorithms	Best	Worst	Mean	Std
COA	4665.174504	20,682.75954	8266.909451	3746.995955
ICOA	958.0737549	22,952.13598	8457.336846	7263.332382
GWO	3418.088762	8213.622118	4884.43496	1183.893831
AO	62,719.0719	458,528.2129	210,568.1447	10,5726.5923
SO	1814.372174	55,114.30415	52,361.88304	11,897.66929
DO	2013.67956	8161.476092	3554.351297	1807.90875
WFO	13,500.33491	69,337.32777	37,717.87814	17,240.74021
GOA	6351.059315	43,182.56002	14,284.76699	8888.626988
FFA	4267.49196	13,092.37675	7708.85239	2429.408312
GRO	2414.211291	6107.173362	4193.944811	1129.766826
AOA	10,111.4088	33,120.087	14,552.68101	5066.244501

**Table 11:** Results of welded beam design problem.

Algorithms	Variables				Optimum Value
	*coa*_1_	*coa*_2_	*coa*_3_	*coa*_4_	
COA	0.205729747	3.253818479	9.03654619	0.205734385	1.69536477301484
ICOA	0.205729878	3.253115802	9.036623911	0.20572964	1.69524705320638
GWO	0.207136169	3.271908158	9.007111301	0.207370174	1.70713835001213
HHO	0.20224206	3.327654143	9.411296372	0.204699696	1.75634507460288
SO	0.199669651	3.52817999	9.014651388	0.206861551	1.72792710447185
DO	0.205733672	3.253068635	9.036565154	0.205732524	1.69526036151233
WFO	0.180799875	4.232650243	8.91411226	0.214390166	1.82921020034696
GOA	0.328329978	2.295504799	7.191643387	0.328402222	2.1249262944582
SSA	0.1	0.1	0.1	0.1	2.25348137991264
ISSA	0.176382124	3.844685095	9.167517271	0.211759511	1.79876399875485
FFA	0.261895948	2.669207594	8.206734753	0.289033286	2.10450411846326
AOA	0.188910224	3.674031117	10	0.202829975	1.86950299444208

**Table 12:** Statistical results of welded beam design problems.

Algorithms	Best	Worst	Mean	Std
COA	1.69536477301484	1.704661863	1.696077909	0.002032594
ICOA	1.69524705320638	1.695279935	1.695249402	7.2712× 10^−6^
GWO	1.70713835001213	1.92234429	1.83040074	0.047717425
HHO	1.75634507460288	2.19512924	1.878052523	0.109369283
SO	1.72792710447185	2.340851079	1.963653238	0.161335053
DO	1.69526036151233	1.708893175	1.700430465	0.00478447
WFO	1.82921020034696	2.909820771	2.297240026	0.352133884
GOA	2.1249262944582	3.920844071	2.726666637	0.550699826
SSA	2.25348137991264	616,699.1066	62,436.4806	149,720.9795
ISSA	1.79876399875485	8.127990023	2.640399736	1.355838167
FFA	2.10450411846326	3.177283012	2.667251605	0.318784137
AOA	1.86950299444208	2.654295467	2.244211056	0.281180602

**Table 13:** Results of robot gripper arm design problem.

Algorithms	Variables							Optimum Value
	*ic*_1_	*ic*_2_	*ic*_3_	*ic*_4_	*ic*_5_	*ic*_6_	*ic*_7_	
COA	99.98571581	38.18002065	199.9772657	0	10.16050765	100	1.479232863	7.2865327450E−17
ICOA	100.0000044	38.19655187	199.9999998	0	16.75042424	100	1.564501204	7.2740693811E−17
SCA	96.43609948	33.95248713	186.8158442	0	38.3396936	100	1.738940081	1.1383886011E−16
AO	108.4779393	10	161.742106	0	150	100	3.14	1.2555430736E−15
BWO	98.75999016	36.28125241	200	0	28.3460658	100	1.556346256	8.9127469379E−17
DO	99.99998971	38.19656335	200	0	126.8168469	100	2.097808015	7.2740793402E−17
WFO	142.5464321	130.5210616	182.2920037	8.150600978	126.6622149	163.4416348	2.556844972	5.2614674772E+00
GOA	98.42713966	36.32268301	129.7971558	0	27.98004263	100	1.606259461	1.3372373669E−16
SSA	10	10	100	0	10	100	1	3.4694372699E+102
RSA	99.60199902	75.18022507	147.2977184	8.353478481	138.7177764	150.0916274	3.14	1.0268188818E+01
FFA	100.5723118	30.97482821	100	0	10	100	1	3.5568574350E−16
GRO	144.7323872	113.5823517	190.4171935	29.49193114	148.850327	134.6732338	2.860226854	3.0270547513E+00
AOA	81.76879862	18.92275265	200	0	119.0401543	100	3.14	2.6163653784E−16

**Table 14:** Statistical results of robot gripper arm design problems.

Algorithms	Best	Worst	Mean	Std
COA	7.2865327450E−17	6.782631033	3.179681189	1.720743265
ICOA	7.2740693811E−17	3.508021841	0.503689008	1.231070738
SCA	1.1383886011E−16	2.92765E−16	1.73937E−16	5.56391E−17
AO	1.2555430736E−15	8.578634281	3.71718948	3.555871938
BWO	8.9127469379E−17	3.93736E−16	1.80241E−16	7.65703E−17
DO	7.2740793402E−17	2.984670765	0.557550382	1.145282181
WFO	5.2614674772E+00	93.93936293	14.84181454	19.17351114
GOA	1.3372373669E−16	10.43372632	5.497079008	4.694065699
SSA	3.4694372699E+102	6.7583E+105	1.5136E+105	1.8813E+105
RSA	1.0268188818E+01	1.3142E+105	1.1547E+104	2.9275E+104
FFA	3.5568574350E−16	5.351968761	1.26434572	2.031519024
GRO	3.0270547513E+00	4.740811605	3.840954694	0.463312144
AOA	2.6163653784E−16	6.793260789	2.313355989	2.479617989

**Table 15:** Results of cantilever beam design problem.

Algorithms	Variables					Optimum Value
	*ic*_1_	*ic*_2_	*ic*_3_	*ic*_4_	*ic*_5_	
COA	5.973239172	5.27141733	4.46232299	3.476567711	2.13734259	13.3169948668511
ICOA	5.973220012	5.271406257	4.462358417	3.47656667	2.137352002	13.3169948659626
SCA	6.197047737	4.896502134	4.587031006	3.711254531	1.94626838	13.4483502463377
AO	5.952877732	5.27980211	4.472859938	3.469528696	2.149892098	13.3172651303013
BWO	6.093914535	5.245174582	4.454005615	3.425590107	2.10260378	13.3219019616545
WFO	5.31695503	7.090411451	4.212123976	3.88342926	2.137997013	14.0917065721423
GOA	5.818535006	5.327608816	4.529575276	3.468355812	2.1806418	13.3254317182751
FFA	5.714835131	6.177210979	4.826957342	3.13185277	1.919368391	13.5983591073533
AOA	6.148452552	4.677518444	4.75385561	3.907300242	3.115583487	14.0679269131395

**Table 16:** Statistical results of cantilever beam design problems.

Algorithms	Best	Worst	Mean	Std
COA	13.3169948668511	13.3169954152945	13.3169949326321	1.34E−d07
ICOA	13.3169948659626	13.3169948743986	13.3169948663854	1.88611E−09
SCA	13.4483502463377	13.9348970502045	13.6340801048167	0.14898056
AO	13.3172651303013	13.3293166175376	13.321791966169	0.002885066
BWO	13.3219019616545	13.3962719533998	13.3461728159391	0.019786476
WFO	14.0917065721423	29.032258369552	19.0207058242824	4.048134
GOA	13.3254317182751	13.6962531075075	13.4207488075685	0.099786135
SSA	42.5490746330585	107.187171632897	77.1815851084039	16.7616407
FFA	13.5983591073533	17.0318475424042	14.9242652183795	0.970489372
AOA	14.0679269131395	37.6119650045082	19.9525131528094	6.760253941

**Table 17:** Results of heat exchanger design issues.

Algorithms	Variables								Optimum Value
	*ic*_1_	*ic*_2_	*ic*_3_	*ic*_4_	*ic*_5_	*ic*_6_	*ic*_7_	*ic*_8_	
COA	462.5427607	1013.963305	5903.791795	154.3961403	264.1452697	244.3227926	289.7539322	364.0891882	7380.297861
ICOA	648.1441113	1407.016002	5158.063247	173.9996594	293.6775363	226.0003082	280.3220856	393.6775246	7213.223361
GWO	162.8618729	1566.202632	5781.639069	107.0005946	269.0613907	270.4296038	236.4759442	369.0317921	7510.703573
HHO	2428.081726	1066.88741	5250.125875	199.5199501	289.9983826	198.7567003	305.5955845	389.9967552	8745.095011
SO	184.4612151	2100.503684	5177.788126	116.4323451	292.888505	282.6675462	221.4407806	392.888496	7462.753025
DO	866.6996969	1000.013304	5547.182004	180.6688392	278.1145075	200.891534	302.4952316	378.1138789	7413.895005
WFO	941.2057817	6999.249432	6530.665654	75.65402374	338.3660656	228.1608045	135.4661082	403.8414835	14,471.12087
GOA	465.7973521	2851.365071	4591.27227	133.8214808	317.6517498	203.863732	215.9026524	417.0136888	7908.434694
BWO	822.4187358	2149.37456	5229.952691	170.5531089	312.1534096	221.9056041	258.209572	403.7764069	8201.745986
ISSA	1289.401116	1099.059957	5000.262925	212.0593125	299.9904139	187.9379361	312.0666078	399.9902002	7388.723998
AO	102.9000008	1789.576757	8049.564424	27.7126494	179.5417586	18.01720773	138.725557	279.2501878	9942.041182
GRO	175.1487385	1456.21826	5792.948411	114.8347834	268.2822482	284.8061069	246.5522545	368.2821795	7274.950879
AOA	7491.185496	1326.515164	10000	98.17921924	198.5000802	257.1230991	226.1767422	279.214993	18,817.70066

**Table 18:** Statistical results of heat exchanger design problems.

Algorithms	Best	Worst	Mean	Std
COA	7380.297861	8964.851977	8051.946612	407.6809122
ICOA	7213.223361	7215.811361	7213.489702	0.520710395
GWO	7510.703573	17,167.02265	8424.547089	1677.0323
HHO	8745.095011	113,030.6782	25,639.5225	23,418.86501
SO	7462.753025	12,888.0598	8849.282895	1128.8162
DO	7413.895005	10,220.21106	8331.891441	596.9471728
WFO	14,471.12087	110,916.386	49,893.10324	25,381.69323
GOA	7908.434694	38,248.44647	17,525.92797	9165.578149
BWO	8201.745986	23,947.17552	11,544.59647	3519.229927
ISSA	7388.723998	40,162.5	11,478.79299	9054.672031
AO	9942.041182	138,486.549	29,114.07484	31,314.68264
GRO	7274.950879	7862.407058	7465.570478	132.9925336
AOA	18,817.70066	91,992.0306	38,459.94464	16,725.11458

**Table 19:** The optimization results of each algorithm on F1–F20.

Function	Goal	COA	ICOA	GWO	HHO	SO	DO	WFO	GOA	BWO	ISSA	AO	GRO	AOA	SaDE
F1	0	7.95803E−16	0	3.06555E−09	0	0	1.39944E−15	3.13344E−06	7.37883E−16	1.02931E−05	0	1.62567E−06	0	1.22759E−05	0
F2	0	0	0	0	0	0	0	0.000963419	0	0	0	0	0	0	0
F3	0.3979	0.397887358	0.397887358	0.397887392	0.397887358	0.397887358	0.397887358	0.397892042	0.397887358	0.411146577	0.397887358	0.397894023	0.397887358	0.398178477	0.397887358
F4	0	0.050756215	0.041541135	0.044175043	0.009094237	0.007225346	0.010529299	0.371742117	0.05	0.316630171	0.000118097	0.279474725	0.001368297	0.055302367	0.000470248
F5	0	0	0	0	7.085E−240	0	7.91521E−32	8.88831E−06	0	0	0	1.3419E−309	0	0	1.6168E−196
F6	0	1.79489E−07	2.5162E−20	1.94102E−05	4.04155E−08	1.60281E−06	4.29879E−05	1.928139815	0	5.3038E−05	1.96417E−08	3.4404E−07	3.17417E−11	0.00744707	7.26818E−28
F7	−2.0626	−2.062611871	−2.062611871	−2.062611871	−2.062611871	−2.062611871	−2.062611871	−2.062611589	−2.062611871	−2.062611871	−2.062611871	−2.062611871	−2.062611871	−2.062611871	−2.062611871
F8	0.998	0.998003838	0.998003838	0.998003838	0.998003838	0.998003838	0.998003838	0.998003839	0.998003838	0.998003838	0.998003838	0.998003838	0.998003838	0.998003838	0.998003838
F9	−1	−1	−1	−1	−1	−1	−1	−0.998323161	−1	−1	−1	−1	−1	−1	−1
F10	−1	−1	−1	−0.999999994	−0.999999999	−1	−1	−0.596052289	−1	−1	−1	−0.99999998	−1	−0.999991609	−1
F11	−959.641	−959.6406627	−959.6406627	−959.6406627	−959.6406627	−959.6406627	−959.6406627	−954.2726351	−959.640144	−959.6406626	−959.6406627	−959.6406625	−959.6406627	−959.6406605	−959.6406627
F12	3	3	3	3.000000144	3	3	3.000000001	3.372155045	3	3.031408621	3	3.001781149	3	3	3
F13	−3.8628	−3.862779787	−3.862779787	−3.862719813	−3.862774994	−3.862779787	−3.862779785	−3.855931775	−3.862779784	−3.862514692	−3.862779787	−3.857798555	−3.862779787	−3.859632062	−3.862779787
F14	−3.1355	−3.134494141	−3.134494141	−3.134488544	−3.133280487	−3.134494141	−3.134494139	−2.988602842	−3.134460663	−3.127934785	−3.134494141	−3.129923381	−3.134494138	−3.125722955	−3.134494141
F15	−3.3224	−3.322367983	−3.322367263	−3.322283466	−3.194634299	−3.322367968	−3.322367746	−2.470374497	−3.312487035	−3.27368502	−3.322368011	−3.248899166	−3.322367366	−3.144075779	−3.322367996
F16	−19.2085	−19.20850257	−19.20850257	−19.20849716	−19.20850257	−19.20850257	−19.20850257	−18.77858166	−19.20850257	−19.20813946	−19.20850257	−19.20832906	−19.20850257	−19.20850226	−19.20850257
F17	−4.1558	−4.155786006	−4.155809292	−4.155804085	−4.155809292	−4.155809292	−4.155809291	−4.084770207	−4.154811612	−4.149230676	−4.155809292	−4.152046957	−4.155809292	−4.097316987	−4.155809292
F18	−1.9133	−1.913222955	−1.913222955	−1.913222804	−1.913222955	−1.913222955	−1.913222955	−1.911128474	−1.913222955	−1.913091261	−1.913222955	−1.91322141	−1.913222955	−1.913199577	−1.913222955
F19	0	7.3841E−258	0	1.56218E−40	8.87572E−43	1.65686E−37	1.27491E−19	0.000519169	2.90305E−78	9.95372E−64	0	4.4688E−43	3.94985E−80	0	2.36907E−19
F20	−1.8013	−1.80130341	−1.80130341	−1.801297686	−1.801303405	−1.80130341	−1.80130341	−1.772270204	−1.80130341	−1.80016541	−1.80130341	−1.801294557	−1.80130341	−1.797607951	−1.80130341

**Table 20:** The optimal results of various algorithms on F21–F30 (d = 30).

Function	Goal	COA	ICOA	GWO	HHO	SO	DO	WFO	GOA	BWO	ISSA	AO	GRO	AOA	SaDE
F21	0	8.88178E−16	8.88178E−16	7.99361E−15	8.88178E−16	4.44089E−15	5.25639E−06	14.82431626	8.88178E−16	8.88178E−16	8.88178E−16	8.88178E−16	4.44089E−15	8.88178E−16	5.4184E−08
F22	0	0.666666667	0.666666667	0.666666885	0.170967902	0.24946633	0.666666806	67,938.28157	0.226030912	0.215938567	0.000847213	0.249229283	0.666666668	0.666666667	0.666666667
F23	0	0	0	0.001136708	0	2.9976E−15	0.371966384	356.2659402	0	0	0	0	0	0.053025212	5.8483077
F24	0	0.627597667	4.11113E−06	0.635294288	2.08407E−11	2.32165E−05	4.95983E−07	25.03785766	1.49976E−32	8.1182E−31	4.37709E−16	1.82308E−07	0.180009632	2.331747611	5.58128E−15
F25	0	4.43864E−11	1.32288E−17	1.53939E−08	9.05537E−10	1.00418E−15	9.48455E−12	0.406890511	1.34978E−31	8.24335E−05	8.69894E−29	6.50439E−06	1.05736E−16	6.09368E−06	6.10458E−17
F26	0	0	0	8.08090767	0	0.00553596	20.59437998	357.3590589	0	0	0	0	0	0	201.3498799
F27	0	−1.019172729	−1.019174434	−1.019174183	−1.019174434	−1.019174433	−1.019174434	−1.009587538	−1.019174434	−1.019172619	−1.019174415	−1.019173965	−1.019174434	−1.019145841	−1.019174045
F28	0	6.166E−214	0	0.002612524	2.27384E−34	1.81897E−16	2.940895611	137,590.7152	7.58861E−66	3.53755E−56	0	1.06282E−39	9.31175E−22	2.45574E−70	3067.605705
F29	0	2494.172842	0.000956134	4907.923677	0.000381827	0.00079236	2470.105618	7000.733646	6666.419217	0.000381827	3009.554149	1.555104273	1419.370864	5527.936861	3775.690232
F30	−1174.97997	−1076.027927	−1146.711529	−1041.345823	−1174.984938	−1174.984873	−1160.847916	−739.3406631	−1031.975357	−1174.984971	−1174.984971	−1127.859557	−1160.554286	−620.2374434	−1174.984971

**Table 21:** The optimal results of various algorithms on F21–F30 (d = 100).

Function	Goal	COA	ICOA	GWO	HHO	SO	DO	WFO	GOA	BWO	ISSA	AO	GRO	AOA	SaDE
F21	0	8.88178E−16	8.88178E−16	7.54952E−14	8.88178E−16	4.44089E−15	0.004012032	16.8370513	8.88178E−16	8.88178E−16	8.88178E−16	8.88178E−16	4.44089E−15	8.88178E−16	0.104610124
F22	0	0.666666668	0.666666668	0.666666922	0.166882511	0.250311677	0.666666865	20,744.42729	0.243130652	0.183117307	0.001076197	0.248929377	0.66666667	0.666666667	0.666666667
F23	0	0	0	0	0	0	0.001528428	509.9229864	0	0	0	0	0	56.98378847	0.162929738
F24	0	5.714578526	0.049283027	5.634467802	7.02166E−09	3.76805E−05	0.637289645	224.2325767	1.49976E−32	7.98322E−30	0.000363838	4.59827E−08	3.967385301	8.511994717	0.253489629
F25	0	8.5895E−19	5.22431E−22	1.1093E−08	2.03448E−30	1.34978E−31	1.11652E−16	0.007167564	1.34978E−31	1.66919E−12	1.34978E−31	4.03921E−07	1.34978E−31	1.18039E−06	1.34978E−31
F26	0	0	0	0	0	0	40.34445269	1050.004385	0	0	0	0	0	0	785.9269144
F27	0	−1.019174434	−1.019174434	−1.019174434	−1.019174434	−1.019174434	−1.019174411	−1.018817632	−1.019174434	−1.019174434	−1.019174434	−1.019174413	−1.019174434	−1.019174265	−1.019174434
F28	0	0	0	2.84714E−32	2.3606E−220	3.0845E−177	0.04186974	817,546.1503	0	0	0	1.48E−306	1.4657E−184	1.2653E−37	3.595936325
F29	0	13,944.08211	2.148955178	22,039.57026	0.001280256	0.008231581	15,722.96032	32,973.90717	27,382.26647	0.001272757	13,035.49418	84.67724841	15,863.73876	28,502.43819	26,394.17618
F30	−3916.5999	−3382.608802	−3902.700855	−2661.655296	−3916.616567	−3916.614685	−3576.73468	−1954.098272	−3649.766234	−3916.61657	−3916.616151	−3301.284001	−3371.233377	−1544.590776	−3774.833127

**Table 22:** The optimal results of various algorithms on F21–F30 (d = 500).

Function	Goal	COA	ICOA	GWO	HHO	SO	DO	WFO	GOA	BWO	ISSA	AO	GRO	AOA	SaDE
F21	0	8.88178E−16	8.88178E−16	1.03927E−08	8.88178E−16	4.44089E−15	2.806958274	17.50598652	8.88178E−16	8.88178E−16	8.88178E−16	8.88178E−16	4.44089E−15	0.007473109	12.74325313
F22	0	0.666678897	0.666946059	0.666667183	0.249998003	0.99050359	9974.365056	70,024,486.31	0.998161345	0.19560557	0.295594907	0.250346788	13,942.20928	0.795333472	7,503,030.487
F23	0	0	0	1.79856E−14	0	0	2.932269362	2525.542517	0	0	0	0	0	5323.253883	500.0040974
F24	0	5.23782E−15	4.05874E−29	1.57557E−08	1.63724E−13	1.34978E−31	1.00955E−14	0.016566874	1.34978E−31	1.13062E−09	1.34978E−31	9.24672E−06	1.34978E−31	0.288859195	1.34978E−31
F25	0	5.24E−15	4.06E−29	1.58E−08	1.64E−13	1.35E−31	1.01E−14	0.0166	1.35E−31	1.13E−09	1.35E−31	0.00000925	1.35E−31	0.289	1.35E−31
F26	0	0	0	2.27374E−11	0	0	803.1113094	5849.28267	0	0	0	0	0	0	5738.63403
F27	0	−1.019174433	−1.019174434	−1.019174428	−1.019174434	−1.019174434	−1.019174407	−1.018491532	−1.019174434	−1.019174434	−1.019174428	−1.019173986	−1.019174434	−1.019173968	−1.019174434
F28	0	0	0	5.64931E−12	9.716E−210	6.1291E−160	15,981.28701	32,843,919.29	0	0	0	1.8327E−304	7.9406E−143	13.42545748	5,239,874.427
F29	0	113,693.8435	93,787.47483	131,229.2469	0.031559183	0.343522069	115,349.5417	191,390.6858	130,409.7633	0.006363784	86,034.14495	140,266.6566	147,667.0188	181,790.5584	177,728.0627
F30	−1.96E+04	−12,115.31382	−12,484.51034	−9086.742048	−19,583.08263	−19,583.0815	−13,947.91964	−8638.626515	−18,359.375	−19,583.08285	−19,567.77419	−18,507.25335	−11,705.00585	−4215.269631	−11,566.49805

**Table 23:** The optimal results of various algorithms on F21–F30 (d = 1000).

Function	Goal	COA	ICOA	GWO	HHO	SO	DO	WFO	GOA	BWO	ISSA	AO	GRO	AOA	SaDE
F21	0	8.88178E−16	8.88178E−16	9.54888E−07	8.88178E−16	4.44089E−15	5.028236979	17.67826592	8.88178E−16	8.88178E−16	8.88178E−16	8.88178E−16	4.44089E−15	0.008929686	17.03175179
F22	0	0.666678246	0.666777037	0.666697033	0.249999501	0.999758559	1,888,035.074	161,860,213.3	0.25	0.250000299	0.360328364	0.25022607	13,695,576.94	0.96388784	132,903,477.4
F23	0	0	0	6.99055E−11	0	0	71.75096865	5545.859087	0	0	0	0	0	27,764.51389	3692.772093
F24	0	86.56393159	31.32303767	84.21660026	4.39319E−06	0.000285289	2808.088279	2525.306239	1.49976E−32	3.29719E−28	0.022940021	0.000370069	86.21481974	90.40236865	1883.153516
F25	0	7.37533E−15	1.07836E−25	1.79031E−08	4.37939E−14	1.34978E−31	4.19062E−15	0.087014099	1.34978E−31	4.93075E−10	1.34978E−31	2.59717E−05	1.34978E−31	2.82718E−06	1.34978E−31
F26	0	0	0	1.086833165	0	0	3062.128514	10,882.47635	0	0	0	0	0	2.01959E−05	12,107.0719
F27	0	−1.019174434	−1.019174434	−1.019174406	−1.019174434	−1.019174434	−1.019174398	−1.017444522	−1.01917441	−1.019174434	−1.019174432	−1.019169813	−1.019174434	−1.019173958	−1.019174434
F28	0	0	0	2.2473E−07	1.8194E−200	5.9176E−154	1,159,759.229	123,096,879.1	0	0	0	5.7335E−302	5.3254E−131	84.13474057	76,716,528.52
F29	0	264,763.6763	46,884.42466	308,366.6059	0.622705718	1.104115044	2,688,68.4353	387,129.2203	293,688.6613	0.012727568	211,723.711	330,786.998	331,334.9251	379,589.4864	373,780.5163
F30	−39,165.999	−20,936.71154	−25,019.29056	−14,894.51127	−39,166.15666	−39,165.84764	−23,009.37501	−18,144.68341	−31,035.61711	−39,166.1657	−38,848.70446	−32,474.21484	−19,797.64546	−6980.135197	−21,420.73319

**Table 24:** Statistical results of solving NP 1.

Algorithms\Indicators	Best	Mean	Std	Rank
COA	3,898,584.167	3,898,584.167	0	5
SaDE	3,967,179.751	3,967,179.751	4.9085E−10	8
AO	4,304,761.075	4,304,761.075	9.817E−10	9
AOA	3,926,034.152	3,926,034.152	4.9085E−10	7
DO	3,839,698.352	3,839,698.352	0	4
HHO	3,818,448.364	3,818,448.364	4.9085E−10	3
GWO	3,635,403.804	3,635,403.804	0	2
GOA	3,907,841.503	3,907,841.503	0	6
ICOA	3,581,472.837	3,581,472.837	4.9085E−10	1

**Table 25:** Statistical results for solving the TSP.

Algorithms\Indicators	Best	Mean	Std	Rank
COA	4.03528555	4.03528555	0	4
SaDE	4.080075419	4.080075419	0	5
AO	4.224619984	4.224619984	9.36222E−16	7
AOA	4.832003947	4.832003947	0	9
DO	4.808047137	4.808047137	9.36222E−16	8
HHO	4.085211443	4.085211443	9.36222E−16	6
GWO	4.027825711	4.027825711	9.36222E−16	3
GOA	4.01468012	4.01468012	9.36222E−16	2
ICOA	4.014148636	4.014148636	**0**	1

## Data Availability

All data generated or analyzed during this study are included in this published article.

## Statement

Current research is limited to the intelligent optimization field, which is beneficial primary use engineering optimization and does not pose a threat to public health or national security. Authors acknowledge the dual use potential of the research involving crayfish optimization algorithm and confirm that all necessary precautions have been taken to prevent potential misuse. As an ethical responsibility, authors strictly adhere to relevant national and international laws about DURC. Authors advocate for responsible deployment, ethical considerations, regulatory compliance, and transparent reporting to mitigate misuse risks and foster beneficial outcomes.

## References

[1] Garip Z., Karayel D., Çimen M.E. (2022). A study on path planning optimization of mobile robots based on hybrid algorithm. Concurr. Comput. Pract. Exp..

[2] Wansasueb K., Panmanee S., Panagant N., Pholdee N., Bureerat S., Yildiz A.R. (2022). Hybridised differential evolution and equilibrium optimiser with learning parameters for mechanical and aircraft wing design. Knowl.-Based Syst..

[3] Yuan M., Li Y., Zhang L., Pei F. (2021). Research on intelligent workshop resource scheduling method based on improved NSGA-II algorithm. Robot. Comput.-Integr. Manuf..

[4] Zhan Z.H., Shi L., Tan K.C., Zhang J. (2022). A survey on evolutionary computation for complex continuous optimization. Artif. Intell. Rev..

[5] Merrikh-Bayat F. (2015). The runner-root algorithm: A metaheuristic for solving unimodal and multimodal optimization problems inspired by runners and roots of plants in nature. Appl. Soft Comput..

[6] Mzili T., Rif M.E., Mzili I., Dhiman G. (2022). A novel discrete rat swarm optimization (DRSO) algorithm for solving the traveling salesman problem. Decis. Mak. Appl. Manag. Eng..

[7] Jia H., Sun K., Li Y., Cao N. (2022). Improved marine predators algorithm for feature selection and SVM optimization. KSII Trans. Internet Inf. Syst. (TIIS).

[8] Mzili I., Mzili T., Rif M.E. (2023). Efcient routing optimization with discrete penguins search algorithm for MTSP. Decis Mak. Appl. Manag. Eng..

[9] Liu Q., Li N., Jia H., Qi Q., Abualigah L. (2022). Modifed remora optimization algorithm for global optimization and multilevel thresholding image segmentation. Mathematics.

[10] Das M., Roy A., Maity S., Kar S., Sengupta S. (2022). Solving fuzzy dynamic ship routing and scheduling problem through new genetic algorithm. Decis Mak. Appl. Manag. Eng..

[11] Jia H., Zhang W., Zheng R., Wang S., Leng X., Cao N. (2022). Ensemble mutation slime mould algorithm with restart mechanism for feature selection. Int. J. Intell. Syst..

[12] Qi H., Zhang G., Jia H., Xing Z. (2021). A hybrid equilibrium optimizer algorithm for multi-level image segmentation. Math. Biosci. Eng..

[13] Kennedy J., Eberhart R. Particle swarm optimization. Proceedings of the ICNN’95-International Conference on Neural Networks.

[14] Karaboga D., Basturk B. (2007). A powerful and efcient algorithm for numerical function optimization: Artifcial bee colony (ABC) algorithm. J Glob. Optim..

[15] Dorigo M., Birattari M., Stutzle T. (2006). Ant colony optimization. IEEE Comput. Intell. Mag..

[16] Gandomi A.H., Yang X.S., Alavi A.H. (2013). Cuckoo search algorithm: A metaheuristic approach to solve structural optimization problems. Eng. Comput..

[17] Hashim F.A., Hussien A.G. (2022). Snake optimizer: A novel meta-heuristic optimization algorithm. Knowl. Based Syst..

[18] Braik M., Hammouri A., Atwan J., Al-Betar M.A., Awadallah M.A. (2022). White shark optimizer: A novel bio-inspired meta-heuristic algorithm for global optimization problems. Knowl. Based Syst..

[19] Heidari A.A., Mirjalili S., Faris H., Aljarah I., Mafarja M., Chen H. (2019). Harris hawks optimization: Algorithm and applications. Futur. Gener. Comp. Syst..

[20] Wang L., Cao Q., Zhang Z., Mirjalili S., Zhao W. (2022). Artificial rabbits optimization: A new bio-inspired meta-heuristic algorithm for solving engineering optimization problems. Eng. Appl. Artif. Intell..

[21] Zhao W., Wang L., Mirjalili S. (2022). Artificial hummingbird algorithm: A new bio-inspired optimizer with its engineering applications. Comput. Methods Appl. Mech. Eng..

[22] Rashedi E., Nezamabadi-Pour H., Saryazdi S. (2009). GSA: A gravitational search algorithm. Inf. Sci..

[23] Kirkpatrick S., Gelatt C.D., Vecchi M.P. (1983). Optimization by simulated annealing. Science.

[24] Rajeev S., Krishnamoorthy C.S. (1992). Discrete optimization of structures using genetic algorithms. J. Struct. Eng..

[25] David B.F. (1998). Artificial Intelligence through Simulated Evolution. Evol. Comput. Foss. Rec. IEEE.

[26] Storn R., Price K. (1997). Differential evolution-A simple and efficient heuristic for global optimization over continuous spaces. J. Glob. Optim..

[27] Jaderyan M., Khotanlou H. (2016). Virulence optimization algorithm. Appl. Soft Comput..

[28] Wen C., Jia H., Wu D., Rao H., Li S., Liu Q., Abualigah L. (2022). Modifed remora optimization algorithm with multistrategies for global optimization problem. Mathematics.

[29] Geem Z.W., Kim J.H., Loganathan G.V. (2001). A new heuristic optimization algorithm: Harmony search. Simulation.

[30] Rao R.V., Savsani V.J., Vakharia D.P. (2012). Teaching–learning-based optimization: An optimization method for continuous non-linear large scale problems. Inf. Sci..

[31] Satapathy S., Naik A. (2016). Social group optimization (SGO): A new population evolutionary optimization technique. Complex. Intell. Syst..

[32] Zhang Y., Jin Z. (2016). Group teaching optimization algorithm: A novel metaheuristic method for solving global optimization problems. Expert Syst. Appl..

[33] Cheng S., Qin Q., Chen J., Shi Y. (2016). Brain storm optimization algorithm: A review. Artif. Intell. Rev..

[34] Xing B., Gao W.J., Xing B., Gao W.J., Kacprzyk J., Jain L.C. (2014). Imperialist competitive algorithm. Innovative Computational Intelligence: A Rough Guide to 134 Clever Algorithms.

[35] Abualigah L., Elaziz M.A., Khasawneh A.M., Alshinwan M., Ibrahim R.A., Alqaness M.A., Mirjalili S., Sumari P., Gandomi A.H. (2022). Meta-heuristic optimization algorithms for solving real-world mechanical engineering design problems: A comprehensive survey, applications, comparative analysis, and results. Neural Comput. Appl..

[36] Zandavi S.M., Chung V.Y.Y., Anaissi A. (2019). Stochastic dual simplex algorithm: A novel heuristic optimization algorithm. IEEE Trans. Cybern..

[37] Dong W., Zhou M. (2016). A supervised learning and control method to improve particle swarm optimization algorithms. IEEE Trans. Syst. Man Cybern. Syst..

[38] Črepinšek M., Liu S.H., Mernik M. (2013). Exploration and exploitation in evolutionary algorithms: A survey. ACM Comput. Surv..

[39] Ezugwu A.E., Shukla A.K., Nath R., Akinyelu A.A., Agushaka J.O., Chiroma H., Muhuri P.K. (2021). Metaheuristics: A comprehensive overview and classification along with bibliometric analysis. Artif. Intell. Rev..

[40] Wolpert D.H., Macready W.G. (1997). No free lunch theorems for optimization. IEEE Trans. Evol. Comput..

[41] Jia H., Rao H., Wen C., Mirjalili S. (2023). Crayfish optimization algorithm. Artif. Intell. Rev..

[42] Xu Y.P., Tan J.W., Zhu D.J., Ouyang P., Taheri B. (2021). Model identification of the proton exchange membrane fuel cells by extreme learning machine and a developed version of arithmetic optimization algorithm. Energy Rep..

[43] Gandomi A.H., Yang X.S., Talatahari S., Alavi A.H. (2013). Firefly algorithm with chaos. Commun. Nonlinear Sci. Numer. Simul..

[44] Fister I., Perc M., Kamal S.M., Fister I. (2015). A review of chaos-based firefly algorithms: Perspectives and research challenges. Appl. Math. Comput..

[45] Lu X.L., He G. (2021). QPSO algorithm based on Lévy flight and its application in fuzzy portfolio. Appl. Soft Comput..

[46] Deepa R., Venkataraman R. (2021). Enhancing whale optimization algorithm with levy flight for coverage optimization in wireless sensor networks. Comput. Electr. Eng..

[47] Zhang Z. (2018). A flower pollination algorithm based on t-distribution elite retention mechanism. J. Anhui Univ. Sci. Technol. (Nat. Sci. Ed.).

[48] Trojovska E., Dehghani M., Trojovský P. (2022). Fennec Fox Optimization: A New Nature-Inspired Optimization Algorithm. IEEE Access.

[49] Shehadeh H. (2022). Chernobyl Disaster Optimizer (CDO): A Novel Metaheuristic Method for Global Optimization. Neural Comput. Appl..

[50] Mirjalili S., Mirjalili S.M., Lewis A. (2014). Grey Wolf Optimizer. Adv. Eng. Softw..

[51] Trojovská E., Dehghani M., Trojovský P. (2022). Zebra Optimization Algorithm: A New Bio-Inspired Optimization Algorithm for Solving Optimization Algorithm. IEEE Access.

[52] Xue J., Shen B. (2020). A novel swarm intelligence optimization approach: Sparrow search algorithm. Syst. Sci. Control Eng. Open Access J..

[53] Mzili T., Mzili I., Riffi M.E. (2023). Artificial rat optimization with decision-making: A bio-inspired metaheuristic algorithm for solving the traveling salesman problem. Decis. Mak. Appl. Manag. Eng..

[54] Mzili T., Mzili I., Riffi M.E., Kurdi M., Ali A.H., Pamucar D., Abualigah L. (2024). Enhancing COVID-19 vaccination and medication distribution routing strategies in rural regions of Morocco: A comparative metaheuristics analysis. Inform. Med. Unlocked.

[55] Sansawas S., Roongpipat T., Ruangtanusak S., Chaikhet J., Worasucheep C., Wattanapornprom W. Gaussian Quantum-Behaved Particle Swarm with Learning Automata-Adaptive Attractor and Local Search. Proceedings of the 19th International Conference on Electrical Engineering/Electronics, Computer, Telecommunications and Information Technology (ECTI-CON).

[56] Qin A.K., Suganthan P.N. Self-adaptive differential evolution algorithm for numerical optimization. Proceedings of the IEEE Congress on Evolutionary Computation.

[57] Saremi S., Mirjalili S., Lewis A. (2017). Grasshopper Optimisation Algorithm: Theory and application. Adv. Eng. Softw..

[58] Mirjalili S., Lewis A. (2016). The whale optimization algorithm. Adv. Eng. Softw..

[59] Abualigah L., Diabat A., Mirjalili S., Elaziz M.A., Gandomi A.H. (2021). The Arithmetic Optimization Algorithm. Comput. Methods Appl. Mech. Eng..

[60] Nadimi-Shahraki M.H., Taghian S., Mirjalili S. (2021). An improved grey wolf optimizer for solving engineering problems. Expert Syst. Appl..

[61] Song W., Liu S., Wang X., Wu W. An Improved Sparrow Search Algorithm. Proceedings of the 2020 IEEE Intl Conf on Parallel & Distributed Processing with Applications. Big Data & Cloud Computing, Sustainable Computing & Communications, Social Computing & Networking (ISPA/BDCloud/SocialCom/SustainCom).

[62] Cheraghalipour A., Hajiaghaei-Keshteli M., Paydar M.M. (2018). Tree Growth Algorithm (TGA): A novel approach for solving optimization problems. Eng. Appl. Artif. Intell..

[63] Zhao S., Zhang T., Ma S., Chen M. (2022). Dandelion optimizer: A nature-inspiredmetaheuristic algorithm for engineering applications. Eng. Appl. Artif. Intell..

[64] Luo K. (2021). Water flow optimizer: A nature-inspired evolutionary algorithm for global optimization. IEEE Trans. Cybern..

[65] Santos I.F. (2018). Controllable sliding bearings and controllable lubrication principles—An overview. Lubricants.

[66] Ragsdell K.M., Phillips D.T. (1976). Optimal design of a class of welded structures using geometric programming. J. Eng. Ind..

[67] Osyczka A., Krenich S., Karas K. Optimum design of robot grippers using genetic algorithms. Proceedings of the Third World Congress of Structural and Multidisciplinary Optimization, (WCSMO).

[68] Mirjalili S. (2016). SCA: A Sine Cosine Algorithm for solving optimization problems. Knowl.-Based Syst..

[69] Abualigah L., Yousri D., Abd Elaziz M., Ewees A.A., Al-Qaness M.A., Gandomi A.H. (2021). Aquila Optimizer: A novel meta-heuristic optimization algorithm. Comput. Ind. Eng..

[70] Abualigah L., Abd Elaziz M., Sumari P., Geem Z.W., Gandomi A.H. (2022). Reptile Search Algorithm (RSA): A nature-inspired meta-heuristic optimizer. Expert Syst. Appl..

[71] Zhong C., Li G., Meng Z. (2022). Beluga whale optimization: A novel nature-inspired metaheuristic algorithm. Knowl.-Based Syst..

[72] Zolfi K. (2023). Gold rush optimizer: A new population-based metaheuristic algorithm. Oper. Res. Decis..

[73] Thanedar P.B., Vanderplaats G.N. (1995). Survey of Discrete Variable Optimization for Structural Design. J. Struct. Eng..

[74] Floudas C.A., Ciric A.R. (1989). Strategies for overcoming uncertainties in heat exchanger network synthesis. Comput. Chem. Eng..

[75] Yang Z. (2023). AFO Solving Real-World Problems.

[76] Houssein E.H., Zaki G.N., Diab A.A.Z., Younis E.M.G. (2021). An efficient Manta Ray Foraging Optimization algorithm for parameter extraction of three-diode photovoltaic model. Comput Electr. Eng..

[77] Houssein E.H., Hussain K., Abualigah L., Elaziz M.A., Alomoush W., Dhiman G., Djenouri Y., Cuevas E. (2021). An improved opposition-based marine predators algorithm for global optimization and multilevel thresholding image segmentation. Knowl.-Based Syst..

